# Pathogenicity and zoonotic potential of a novel pdm09-lineage H1N2 swine influenza A virus spatiotemporally linked to transboundary pig trade

**DOI:** 10.1371/journal.ppat.1014779

**Published:** 2026-11-03

**Authors:** Seung-Chai Kim, Hwan-Ju Kim, Bitgoeul Kim, Gyeong-Seo Park, Byeong-Joo Seo, Chonghan Kim, Chang-Gi Jeong, Jae-Ku Oem, Sung-Hyun Moon, Se-Kyung Jung, Yeonsu Oh, Ho-Seong Cho, Yu Kyung Kim, Chung-Young Lee, Won-Il Kim

**Affiliations:** 1 College of Veterinary Medicine, Jeonbuk National University, Iksan, Republic of Korea; 2 Department of Microbiology, School of Medicine, Kyungpook National University, Daegu, Republic of Korea; 3 WOOGENE B&G Co., LTD., Seoul, Republic of Korea; 4 College of Veterinary Medicine and Institute of Veterinary Science, Kangwon National University, Chuncheon, Republic of Korea; 5 Department of Clinical Pathology, School of Medicine, Kyungpook National University, Daegu, Republic of Korea; 6 Untreatable Infectious Disease Institute, Kyungpook National University, Daegu, Republic of Korea; Friedrich-Loeffler-Institute: Friedrich-Loeffler-Institut Bundesforschungsinstitut fur Tiergesundheit, GERMANY

## Abstract

International swine trade can disseminate influenza A viruses across borders, creating opportunities for reassortment with endemic swine viruses and the emergence of novel strains with zoonotic potential. Here, we characterize a novel H1N2 reassortant, A/swine/Korea/1030-3/2023 (1030-3), detected in South Korea and spatiotemporally associated with European swine lineages and international pig movement. 1030-3 harbors seven gene segments (PB2, PB1, PA, HA, NP, M and NS) closely related to a recent Danish zoonotic H1N1 virus of the 2009 pandemic (pdm09) lineage, and an N2 segment derived from an endemic Korean HxN2. *In vitro* assays demonstrated that 1030-3 preferentially bound human-type α2,6-linked sialic acid receptors and replicated efficiently in polarized human airway epithelial cells, although these properties were also observed in the endemic Korean H1N2 comparator 1027-1. In mice, 1030-3 caused moderate clinical signs and respiratory replication comparable to an early pdm09 reference strain NL09. In guinea pigs, 1030-3 transmitted to a subset of direct-contact animals but showed no detectable aerosol transmission under the conditions tested. In pigs, the natural host, 1030-3 replicated efficiently and transmitted to all direct-contact animals, with high respiratory shedding and a greater cumulative shedding burden in contact pigs than NL09. Contemporary human sera showed reduced neutralizing activity against 1030-3 relative to both human seasonal H1N1 and 1027-1, while antiserum against a recent H1N1 vaccine antigen also neutralized 1030-3 less efficiently. These findings identify a European-associated H1N2 swine influenza reassortant detected in South Korea that combines a distinct pdm09-derived genomic and antigenic background with efficient replication and transmission in pigs, α2,6-linked receptor preference, and reduced neutralization by human sera, underscoring the need for trade-aware surveillance of swIAVs and continued monitoring at the swine-human interface.

## Author summary

Swine influenza A viruses are important animal pathogens and recurring sources of viruses with pandemic potential. International movement of pigs can introduce influenza viruses into new regions, where they may reassort with endemic strains and generate variants whose biological properties cannot be predicted from genetic surveillance alone. In this study, we identified a novel H1N2 swine influenza virus in South Korea that contains a pdm09-derived backbone closely related to European swine viruses, including a recent Danish zoonotic strain, and an N2 segment derived from endemic Korean swine influenza viruses. This virus replicated and transmitted efficiently in pigs and showed preferential binding to human-type receptors and robust growth in human airway epithelial cells. While these phenotypes are not unique to 1030-3, the virus combines them with a distinct pdm09-derived genomic background and reduced neutralization by contemporary human sera compared with endemic Korean swine H1N2 and seasonal H1N1 viruses. Antiserum against a recent H1N1 vaccine antigen also neutralized 1030-3 less efficiently than human pdm09 strains. Together, our findings highlight the importance of monitoring swIAV evolution in the context of international livestock movement and reassortment and support trade-aware surveillance of viruses with antigenic and biological features relevant to the swine–human interface.

## Introduction

Swine influenza A viruses (swIAVs) circulate endemically worldwide and are a major cause of respiratory disease in pigs [1]. Pigs serve as mixing vessels for influenza A viruses (IAVs), facilitating genetic reassortment and the emergence of novel influenza strains [1,2]. The emergence of the novel swine-origin 2009 H1N1 pandemic (pdm09) underscored the global health threat, with the first influenza pandemic of the 21st century [3,4]. This virus spread rapidly worldwide, resulting in significant morbidity and mortality, particularly among at-risk populations such as pregnant women, the elderly, and individuals with preexisting health issues [4,5]. While public health interventions, including vaccination, successfully controlled the immediate spread of the virus, pdm09 has continued to circulate as a seasonal virus [6–8]. Consequently, tracking the post-pandemic evolution of pdm09-derived viruses in swine is essential for identifying reassortants that may reacquire zoonotic potential.

Unlike human seasonal influenza viruses, which typically exhibit a ladder-like phylogeny characterized by successive replacement of antigenic variants driven by host immune pressure and vaccination, swIAVs frequently persist as multiple, region-specific, co-circulating lineages maintained by partial cross-immunity and heterogeneous herd immunity [9]. This diversity largely reflects international trade and recurrent bidirectional transmission between swine and humans, followed by antigenic drift and shift within swine populations [1]. Pig movements through domestic transport and international trade disseminate viral lineages across regions and continents, providing opportunities for the establishment of novel swIAV genotypes and reassortants [3]. A notable example is the emergence in China of the Eurasian avian-like swine (EAS) genotype 4 (G4) reassortant, derived from pdm09 and Eurasian avian-like lineages [10]. Indeed, the remarkable genetic diversity of swIAVs in Chinese, South Korean, Vietnamese, and other Asian herds has been driven largely by intercontinental trade and movement of pigs from Europe and North America [11]. Thus, international pig movement can act not only as a pathway for viral dissemination but also as an ecological bridge connecting otherwise geographically separated swIAV gene pools.

The emergence of novel swIAV variants has been accompanied by human infections, as seen in H1N1v and H1N2v cases derived from the 1B human seasonal lineage in the United Kingdom and the 1C EAS lineage in the Netherlands during the 2020s [12,13]. In addition, sustained human-to-swine transmission and adaptation of the pdm09 lineage in swine further shape the zoonotic risk landscape [14]. Notably, pdm09-derived swine variants, including reassortants with endemic swIAVs, have caused multiple human infections in Denmark in 2021 [15,16], illustrating that swine-adapted descendants of pdm09 can re-enter humans after diversification in pigs. These observations underscore the importance of active surveillance and risk assessment of novel swIAVs to enable early detection and the implementation of effective countermeasures.

Recent active surveillance during 2021–2022 in South Korea revealed the circulation of multiple region-specific swIAV clades, including distinct pdm09-derived swIAV variants [17]. This co-circulation is driving further diversification of swIAVs in South Korea. In this study, we report the detection of a European-associated, pdm09-derived H1N2 swIAV in South Korea in 2023. Genomic analysis showed that this virus is closely related to a Danish zoonotic pdm09-derived swine H1N1 virus but carries a neuraminidase segment from endemic Korean swIAVs, yielding a novel reassortant genotype. We therefore combined genomic epidemiology, phylogeographic inference, in vitro phenotyping, mammalian transmission models, swine infection experiments, and human serology to investigate its evolutionary context, replication and transmission phenotypes, and features relevant to zoonotic-risk assessment.

## Results

### Detection of a European-associated pdm09-derived H1N2 swIAV in South Korea

During nationwide active surveillance of swine influenza A virus in 2023, nasal swabs and pooled oral fluids were collected from pigs with respiratory signs on 40 farms across South Korea. Initial HA screening identified A/swine/Korea/1030-3/2023 (1030-3), an H1N2 virus recovered from the nasal swab of a newly introduced gilt, as a divergent isolate because its HA shared <90% nucleotide identity with previously reported Korean swIAVs. Complete coding sequences for all eight segments were obtained by multisegment RT-PCR, Illumina iSeq sequencing, and de novo assembly. The HA sequence of 1030-3 was then analyzed together with a curated global dataset of H1Nx swIAVs and pdm09-related H1 viruses (Fig 1A).

**Fig 1:**
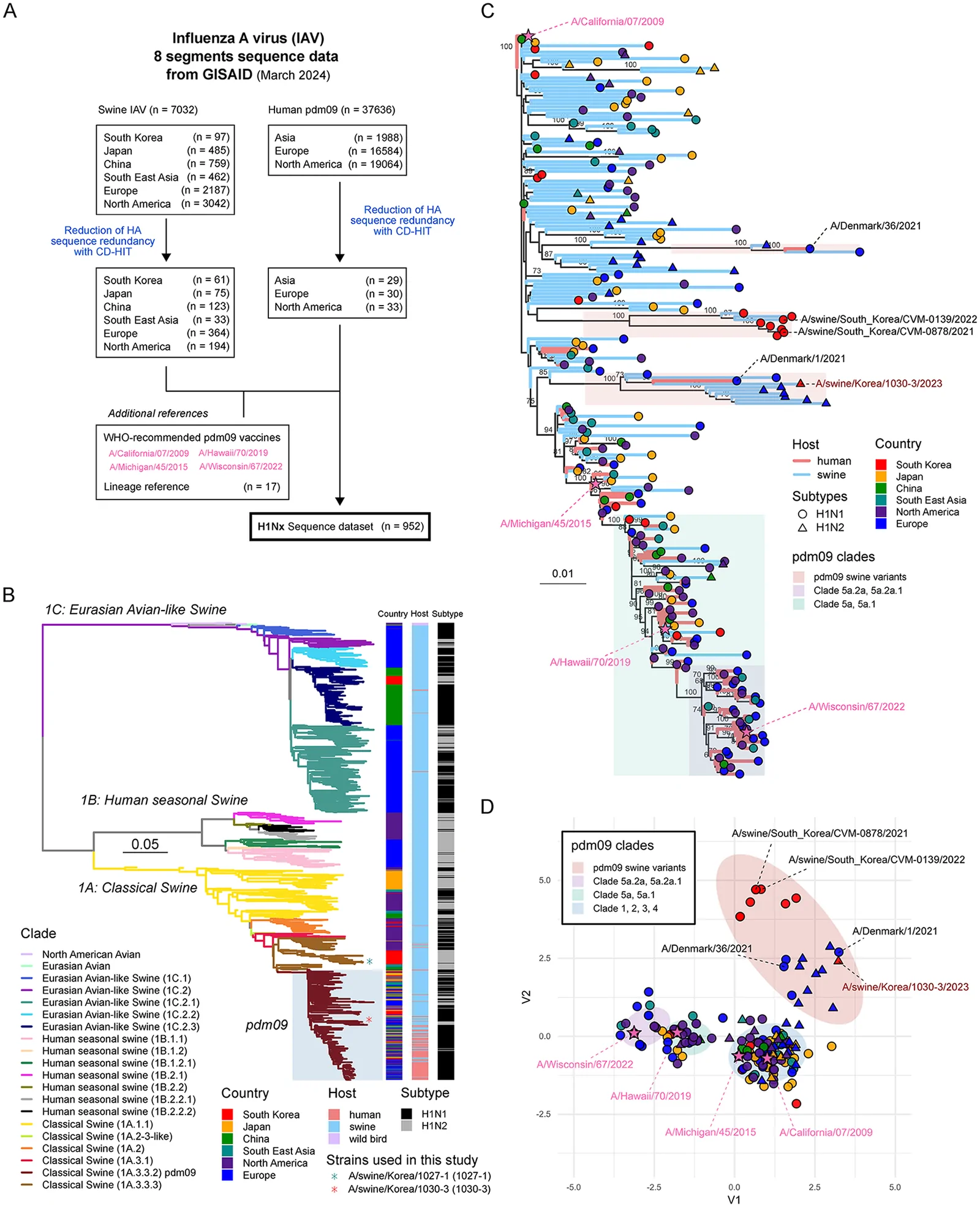
Global phylogenetic structure and antigenic diversification of H1Nx swIAVs. **(A)** Overview of datasets analyzed in this study, including reference H1Nx IAV sequences retrieved from GISAID (n = 7032; filtered to 850 representative HA sequences after CD-HIT clustering) and field samples collected from 40 pig farms in South Korea during 2023 surveillance (12 swIAV isolates subjected to whole-genome sequencing). **(B)** Maximum-likelihood phylogeny of 952 HA (H1) sequences showing three major swIAV lineages: 1A (Classical swine, including the pdm09 clade), 1B (Human seasonal), and 1C (Eurasian avian-like swine). Branches are colored by lineage, and metadata bars indicate country, host, and subtype. **(C)** Subtree of the pdm09 (1A.3.3.2) clade highlighting Korean swIAV isolates from this study (red) that cluster with European pdm09 swine variants (e.g., A/Denmark/1/2021). Node support values (>70) are shown. **(D)** Sequence-based antigenic cartography of pdm09 HA epitopes showing that Korean pdm09 swine variants cluster antigenically with European pdm09 swine variants and form a distinct group separate from human pdm09 viruses.

The maximum-likelihood tree resolved three major H1Nx swIAV lineages: classical swine (CS, 1A), human seasonal swine (1B), and Eurasian avian-like swine (EAS, 1C), each comprising multiple region-specific clades (Fig 1B). The pdm09 lineage (1A.3.3.2) contained both human and swine viruses. Within the pdm09 subtree, 1030-3 clustered with recent European pdm09 swine variants, including the Danish human infection strain A/Denmark/1/2021 [16] (Fig 1C). Sequence-based antigenic cartography of H1 protein further showed that 1030-3 grouped with European pdm09 swine viruses, forming a distinct cluster separate from both early and recent human pdm09 strains (Fig 1D). Antigenic distances (AU) from 1030-3 to A/Denmark/1/2021, A/California/07/2009, and A/Wisconsin/67/2022 were 1.1, 3.8, and 6.9, respectively, underscoring its close antigenic relatedness to European swine variants. Collectively, these analyses place 1030-3 within a European-associated pdm09 swine virus cluster that is antigenically distinct from both early and recent human pdm09 viruses.

### Phylogeographic association and segmental reassortment of European-origin pdm09 swine variant

Time-scaled phylogenetic analysis placed 1030-3 within the European pdm09 swine variant lineage (Fig 2A and 2B). Genotype constellation analysis showed that 1030-3 retained a European pdm09 swine variant backbone closely related to A/Denmark/1/2021 but carried an NA segment derived from the endemic Korean CS 1A.3.3.3 clade, identifying 1030-3 as a novel H1N2 reassortant (Fig 2B and S1 Fig).

**Fig 2:**
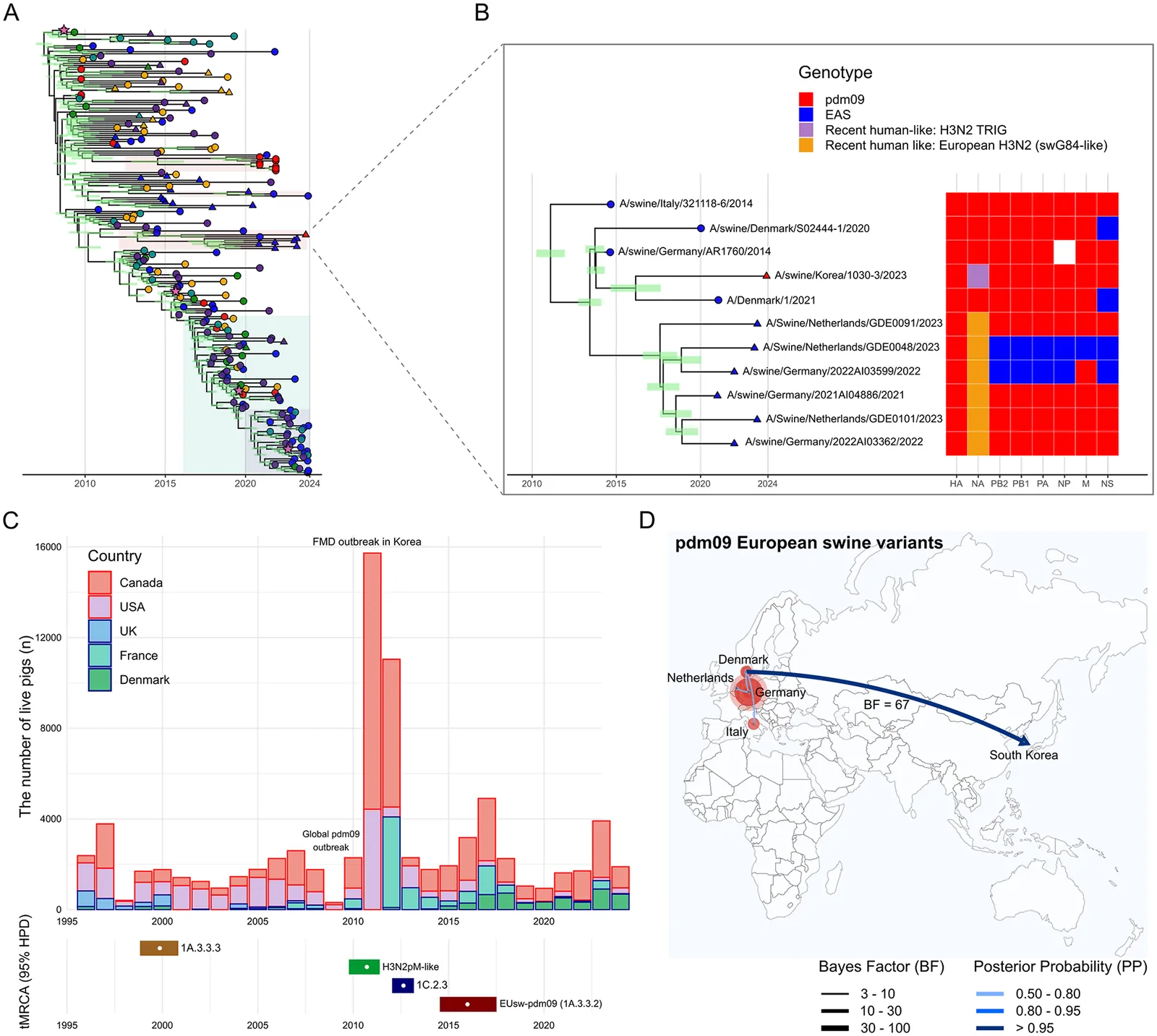
Phylogeographic association and reassortment of European-origin pdm09 swine variants. **(A)** Time-scaled maximum clade credibility (MCC) tree of pdm09 HA sequences showing the placement of Korean isolate A/swine/Korea/1030-3/2023 within European pdm09 swine variants. **(B)** Subtree of European-origin pdm09 swine variants with a genotype constellation heatmap. A/swine/Korea/1030-3/2023 retained the European pdm09 swine variant backbone but incorporated an NA segment from endemic Korean swIAVs (CS 1A.3.3.3 clade). **(C)** Annual records of live-pig imports into Korea (1996–2024), with mean tMRCA estimates and 95% highest posterior density (HPD) intervals for selected swIAV lineages shown below the bar graph. **(D)** Phylogeographic reconstruction of European pdm09 swine variants showing statistically supported diffusion pathways from Denmark and other European countries into Korea (Bayes factor = 67; posterior probability > 0.95).

Live-pig import records showed repeated introductions of pigs into Korea from the United Kingdom, France, Denmark, Canada, and the United States, with increased imports from Denmark after 2015 (Fig 2C, upper panel; S1 Table). We also plotted tMRCA estimates and 95% highest posterior density (HPD) intervals for major swIAV lineages below the importation records (Fig 2C, lower panel). The estimated tMRCA was 1999.9 for the Korean CS 1A.3.3.3 lineage (95% HPD, 1998.8–2000.3), 2010.7 for the H3N2pM-like lineage (95% HPD, 2009.8–2011.4), 2012.6 for the 1C.2.3 lineage (95% HPD, 2012.0–2013.8), and 2016.0 for the European pdm09 swine variant lineage containing 1030-3 (95% HPD, 2014.5–2017.5). Discrete phylogeographic inference identified a statistically supported diffusion pathway from Denmark and neighboring European countries into Korea (Bayes factor = 67; posterior probability > 0.95) (Fig 2D). Collectively, these data support the placement of 1030-3 within a European-associated pdm09 swine lineage and indicate that it acquired an NA segment from an endemic Korean swIAV.

### *In vitro* characterization

We compared the *in vitro* phenotype of 1030-3 with the early pdm09 reference strain NL09 and the endemic Korean H1N2 swIAV 1027-1 by assessing replication in polarized human bronchial epithelial cells, receptor binding, HA fusion pH, and antiviral susceptibility. In Calu-3 cells cultured at an air-liquid interface, all three viruses replicated efficiently, reaching high titers by 48–72 hpi (Fig 3A). In solid-phase binding assays, all three viruses preferentially bound α2,6-linked sialic acid (6′SLN), with minimal binding to α2,3-linked sialic acid (3′SLN) (Fig 3B). The HA fusion thresholds were pH 5.3 for NL09, pH 5.6 for 1027-1, and pH 5.7 for 1030-3 (Fig 3C). Thus, the receptor-binding and fusion characteristics of 1030-3 were broadly similar to those of the endemic Korean H1N2 comparator, although its fusion threshold was slightly higher. 1030-3 remained susceptible to baloxavir and oseltamivir, with IC_50_ values consistent with drug-susceptible influenza strains (Fig 3D). Overall, 1030-3 replicated efficiently in polarized Calu-3 cells and preferentially bound α2,6-linked sialic acids, although these properties were also observed in the endemic Korean H1N2 comparator 1027-1.

**Fig 3:**
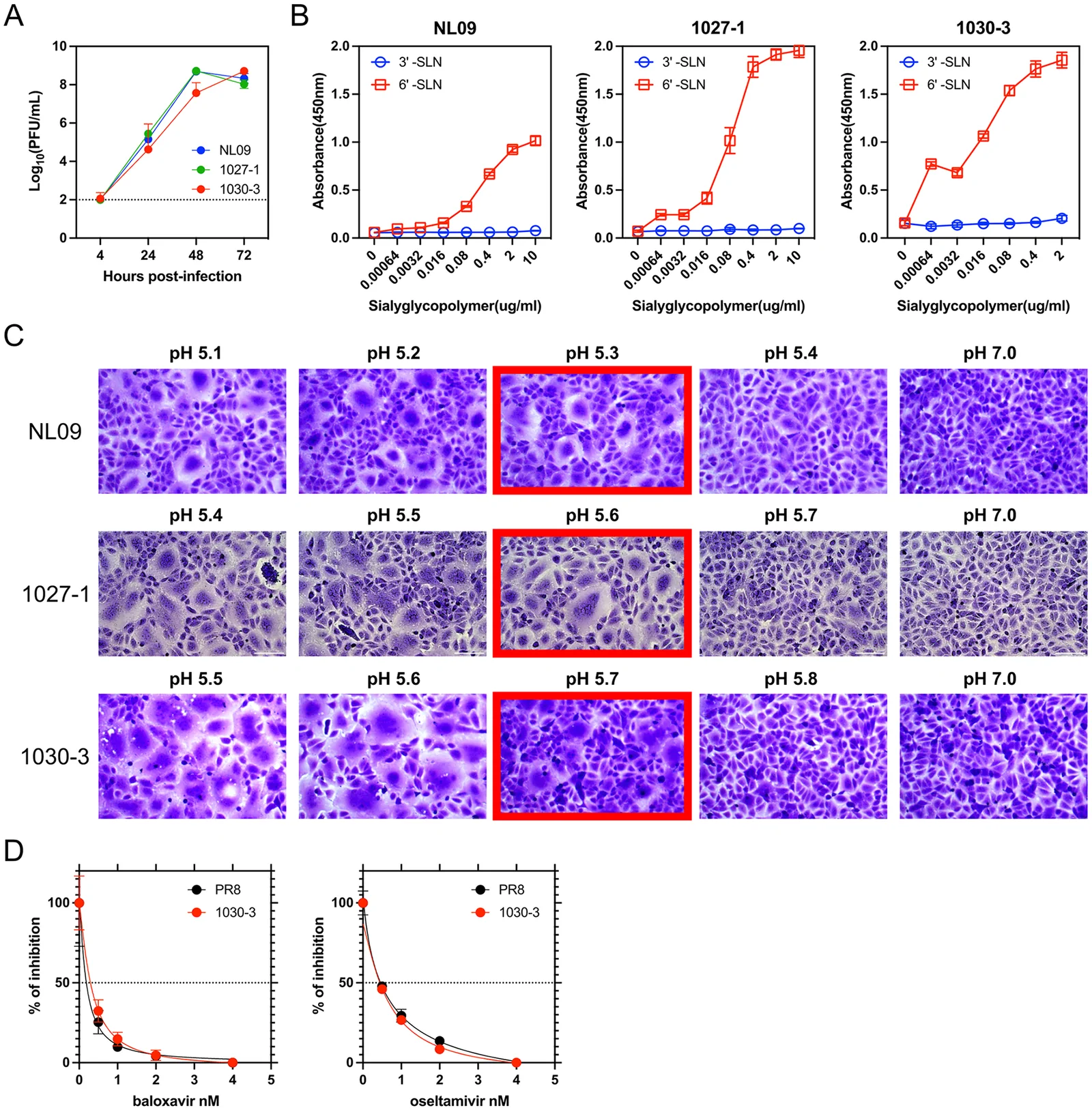
*In vitro* characteristics of the swine H1N2 isolate A/swine/Korea/1030-3/2023 (1030-3). **(A)** Viral replication kinetics in Calu-3 cells. Apical washes were collected at 0, 24, 48, and 72 hpi and titrated by plaque assay. **(B)** Solid-phase binding assays were performed using α2,3-linked (3′SLN) and α2,6-linked (6′SLN) sialylglycopolymers. **(C)** Viral fusion pH thresholds were determined by a syncytium formation assay in Vero cells after 15 min of exposure to pH-adjusted PBS (pH 4.8-7.0). Red boxes indicate the highest pH at which syncytium formation was observed. **(D)** Antiviral susceptibility was evaluated using a plaque reduction test for baloxavir and a neuraminidase inhibition assay for oseltamivir.

### Pathogenicity in mice and transmission in guinea pigs

BALB/c mice were intranasally infected with 1 × 10^5^ PFU of 1030-3 or NL09 and monitored for 14 dpi (Fig 4A). Neither pdm09 virus caused mortality, which is consistent with previous findings [18,19]. Both NL09-infected and 1030-3 mice showed moderate weight loss, reaching a maximum of 7.1% and 4.8%, respectively (Fig 4B). Infectious virus was detected in the lungs and tracheas of both infected groups at 3 and 5 dpi, although 1030-3 generally produced lower tissue titers than NL09, particularly at 5 dpi (Fig 4C).

**Fig 4:**
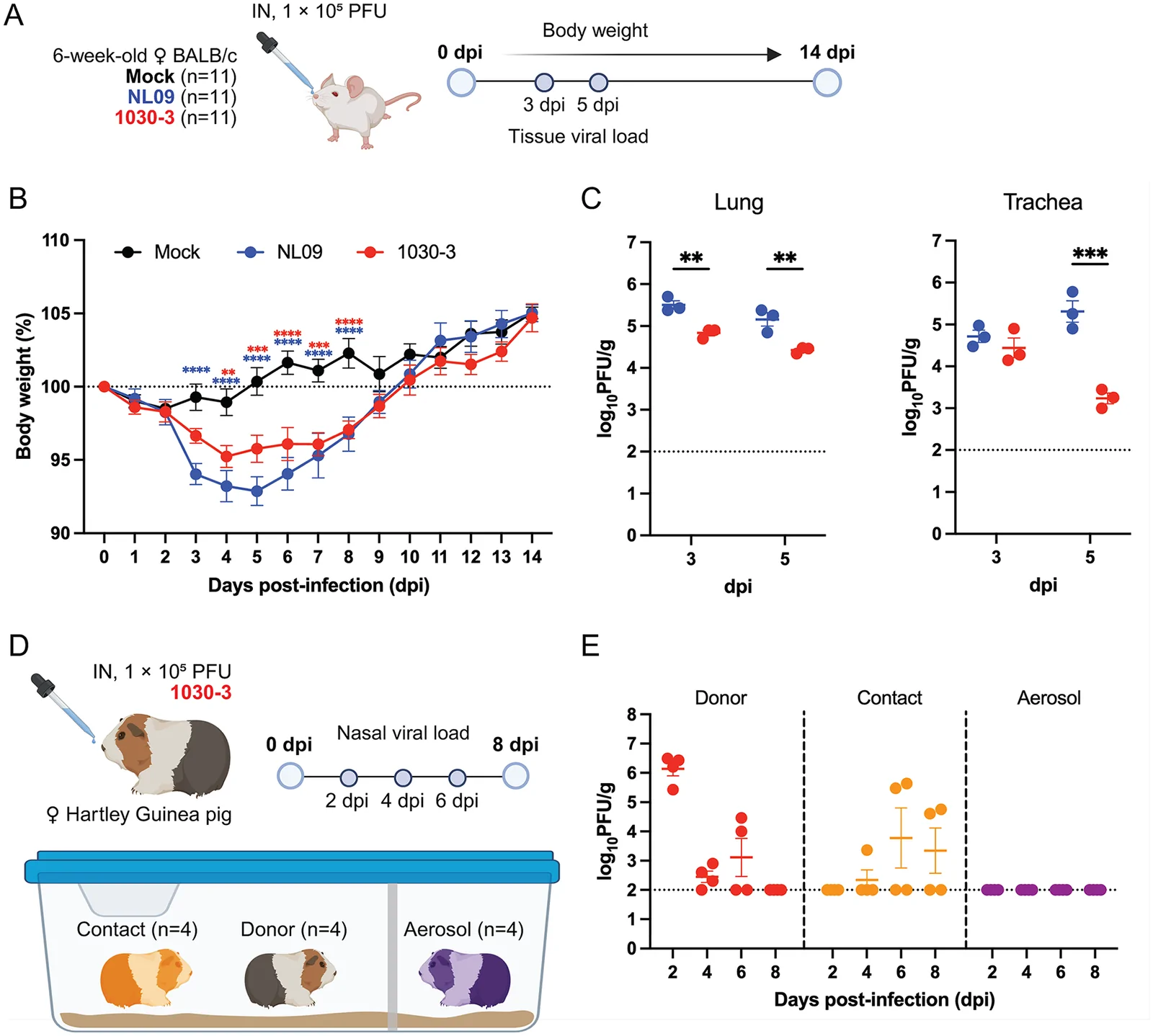
Pathogenicity of A/swine/Korea/1030-3/2023 (1030-3) in mice and transmission in guinea pigs. **(A)** Experimental design for mouse infection. Six-week-old female BALB/c mice were intranasally inoculated with PBS (Mock), NL09, or 1030-3 at 1 × 10^5^ PFU. Body weight was monitored until 14 dpi, and tissues were collected at 3 and 5 dpi for viral titration. **(B)** Body weight changes in infected mice. Data are presented as mean ± SEM. Colored asterisks indicate significant differences between each infected group and the mock group. **(C)** Infectious viral titers in lung and tracheal homogenates at 3 and 5 dpi were measured by plaque assay. The dashed line indicates the limit of detection. **(D)** Experimental design for guinea pig transmission. Donor Hartley guinea pigs were intranasally inoculated with 1 × 10^5^ PFU of 1030-3, and naïve guinea pigs were introduced for direct-contact or aerosol-exposure transmission assessment. **(E)** Infectious viral titers in nasal wash samples from donor, direct-contact, and aerosol-exposed guinea pigs. Each dot represents an individual animal, and bars indicate mean ± SEM. Numbers below the x-axis indicate the number of IAV-positive animals among total animals tested at each time point. The dashed line indicates the limit of detection. *p < 0.05, **p < 0.01, ***p < 0.001. Created in BioRender. Kim, **S.** (2026) https://BioRender.com/elw618b.

We next assessed 1030-3 transmission in guinea pigs by direct-contact and aerosol-exposure routes (Fig 4D). All donor animals, intranasally inoculated with 1 × 10^5^ PFU of 1030-3, shed infectious virus at 2 dpi, with viral shedding declining thereafter. Infectious virus was detected in 2 of 4 direct-contact animals at 2 and 4 dpi. In contrast, no infectious virus was detected in aerosol-exposed animals at any time point (Fig 4E). Overall, 1030-3 replicated in the murine respiratory tract and transmitted to a subset of direct-contact guinea pigs, but no aerosol transmission was detected under the conditions tested.

### Pathogenicity and transmission in swine

We next evaluated the pathogenicity and transmissibility of 1030-3 in 4-week-old piglets, using NL09 as a pdm09 reference strain and the endemic Korean H1N2 swIAV strain 1027-1 as an additional comparator (Fig 5A). All three viruses produced detectable and sustained nasal shedding in directly inoculated pigs. During the acute phase of infection, pigs inoculated with 1027-1 or 1030-3 generally showed higher nasal viral shedding than those inoculated with NL09, resulting in greater cumulative shedding burdens over 0–5 dpi (Fig 5B). Average daily weight gain was reduced in all virus-infected groups relative to mock-infected pigs, with greater reductions in the 1027-1- and 1030-3-infected groups than in the NL09-infected group (Fig 5C).

**Fig 5:**
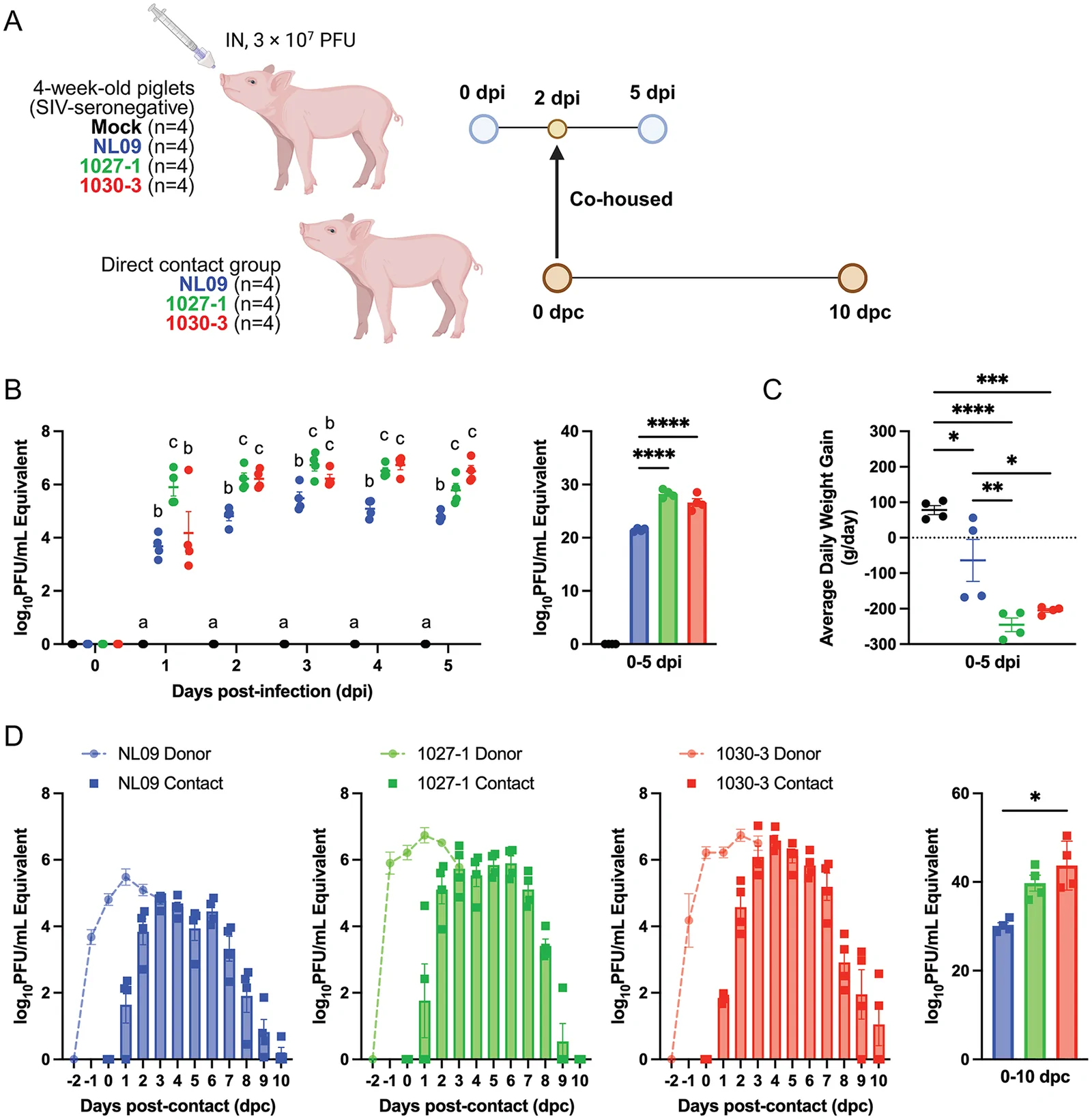
Pathogenicity and direct-contact transmission of A/swine/Korea/1030-3/2023 (1030-3) in pigs. **(A)** Experimental design for pig infection and direct-contact transmission. Four-week-old SIV-seronegative piglets were intranasally inoculated with PBS (Mock), NL09, 1027-1, or 1030-3 at 3 × 10⁷ PFU. Direct-contact pigs were introduced at 2 dpi, corresponding to 0 days post-contact (dpc). Directly inoculated pigs were monitored until 5 dpi, and contact pigs were monitored for nasal shedding until 10 dpc. **(B)** Nasal viral shedding kinetics in directly inoculated pigs from 0 to 5 dpi and cumulative shedding burden, calculated as area under the curve (AUC). **(C)** Average daily weight gain in directly inoculated pigs during 0–5 dpi. **(D)** Nasal viral shedding kinetics in donor and direct-contact pigs and cumulative shedding burden in contact pigs during 0–10 dpc. Donor shedding is plotted relative to the day of contact introduction, with −2 dpc corresponding to donor inoculation. Numbers below the x-axis indicate the number of IAV-positive contact pigs among total contact pigs at each time point. Data are presented as mean ± SEM with individual animals shown. Different lowercase letters indicate significant differences among virus groups at the same time point (*p* < 0.05). *p < 0.05, **p < 0.01, ***p < 0.001, ****p < 0.0001. Created in BioRender. Kim, **S.** (2026) https://BioRender.com/elw618b.

All three viruses transmitted to direct-contact pigs. At 1 dpc, infectious virus was detected in 3 of 4 NL09 contact pigs, 2 of 4 1027-1 contact pigs, and all 4 1030-3 contact pigs. By 2 dpc, all contact pigs in each virus group were positive, indicating efficient direct-contact transmission of all three viruses. Viral shedding subsequently persisted through 10 dpc in the NL09 and 1030-3 groups, whereas no 1027-1 contact pigs were positive at 10 dpc. Contact pigs exposed to 1030-3 showed the highest cumulative nasal shedding burden over 0–10 dpc, which was significantly greater than that observed in the NL09 group and numerically higher than that in the 1027-1 group (Fig 5D). Overall, 1030-3 replicated and transmitted efficiently in pigs and produced sustained viral shedding following contact exposure, although its relative phenotype varied depending on the measured outcome.

### Pathology and local immune responses in respiratory tracts of directly infected piglets

We next examined respiratory pathology, tissue viral burden, and bronchoalveolar lavage (BAL) immune responses in directly infected piglets at 5 dpi. All virus-infected groups developed macroscopic lung lesions, although the severity differed among strains. Pigs infected with 1027-1 showed the highest numerical lesion scores, whereas 1030-3 also produced significantly greater lesions than mock infection (Fig 6A). Viral RNA was detected throughout the respiratory tract in all virus-infected groups. In BALF, viral burden was significantly higher in 1030-3-infected pigs than in NL09-infected pigs, while 1027-1 also produced high viral loads in the lower respiratory tract (Fig 6B).

**Fig 6:**
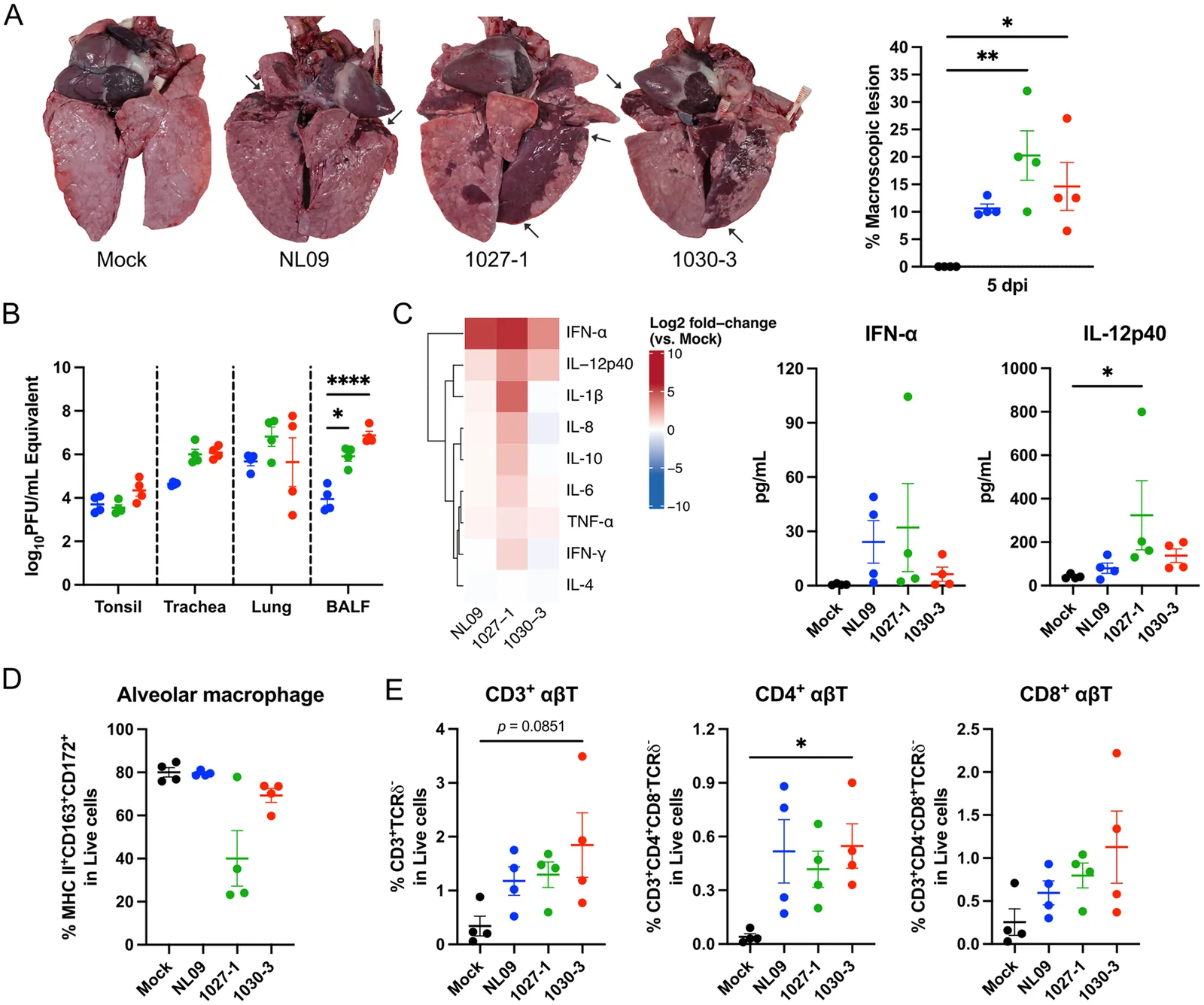
Respiratory pathology, viral burden, and local immune responses in directly infected pigs. **(A)** Representative gross lung images and macroscopic lung lesion scores at 5 dpi. Arrows indicate representative consolidated lung lesions. **(B)** Viral loads in tonsil, trachea, lung, and bronchoalveolar lavage fluid (BALF) at 5 dpi. Viral loads are expressed as log_10_ PFU equivalents per gram of tissue or per mL of BALF. **(C)** Lung cytokine responses at 5 dpi. The heatmap shows log_2_ fold changes relative to mock-infected pigs. Dot plots show IFN-α and IL-12p40 concentrations in lung homogenates. **(D)** Frequencies of alveolar macrophages in BAL cells. Alveolar macrophages were defined as MHC II^+^CD163^+^CD172^+^ cells among live cells. **(E)** Frequencies of αβ T-cell subsets in BAL cells, including CD3 ⁺ TCRδ ⁻ , CD3 ⁺ CD4 ⁺ TCRδ ⁻ , and CD3 ⁺ CD4 ⁻ CD8 ⁺ TCRδ⁻ cells. Data are presented as mean ± SEM with individual animals shown. *p < 0.05, **p < 0.01, ***p < 0.001.

Lung cytokine profiling revealed strain-associated differences at 5 dpi (Fig 6C). Among the individual cytokines examined, IL-12p40 concentrations were significantly increased in 1027-1-infected pigs compared with mock-infected pigs, whereas IFN-α levels showed substantial inter-animal variation and did not differ significantly among groups. Other cytokine responses were generally modest or variable across strains (S2 Fig).

Flow cytometric analysis of BAL cells also showed strain-associated differences at 5 dpi. The frequency of alveolar macrophages was numerically reduced in 1027-1- and 1030-3-infected pigs, with the largest decrease observed in the 1027-1 group, although the differences were not statistically significant (Fig 6D; S3 Fig). In the lymphoid compartment, total αβ T-cell frequencies showed an increasing trend across infected groups (p = 0.0851). The frequency of CD4^+^ αβ T cells was significantly increased in 1030-3-infected pigs relative to mock-infected pigs, whereas CD8^+^ αβ T-cell frequencies showed numerical but non-significant increases (Fig 6E).

Collectively, these findings indicate strain-associated differences in respiratory pathology and the local immune environment at 5 dpi, but do not identify a single pathological or immunological feature uniquely associated with the replication or transmission phenotype of 1030-3.

### Reduced neutralization of 1030-3 by human sera

To assess pre-existing antibody responses against 1030-3, we performed hemagglutination inhibition (HI) assays using human serum samples with documented seasonal vaccination and/or recent influenza infection history (n = 13; S2 Table). The recent human pdm09 H1N1 vaccine strain Hawaii2019 and 1027-1 were included as comparators. HI titers varied substantially among individuals. Compared with Hawaii2019, HI titers were significantly lower against 1027-1 (p = 0.0286), whereas the difference between Hawaii2019 and 1030-3 did not reach statistical significance (Fig 7A). These results indicate heterogeneous HA-directed antibody recognition of 1030-3 within the serum panel examined.

**Fig 7:**
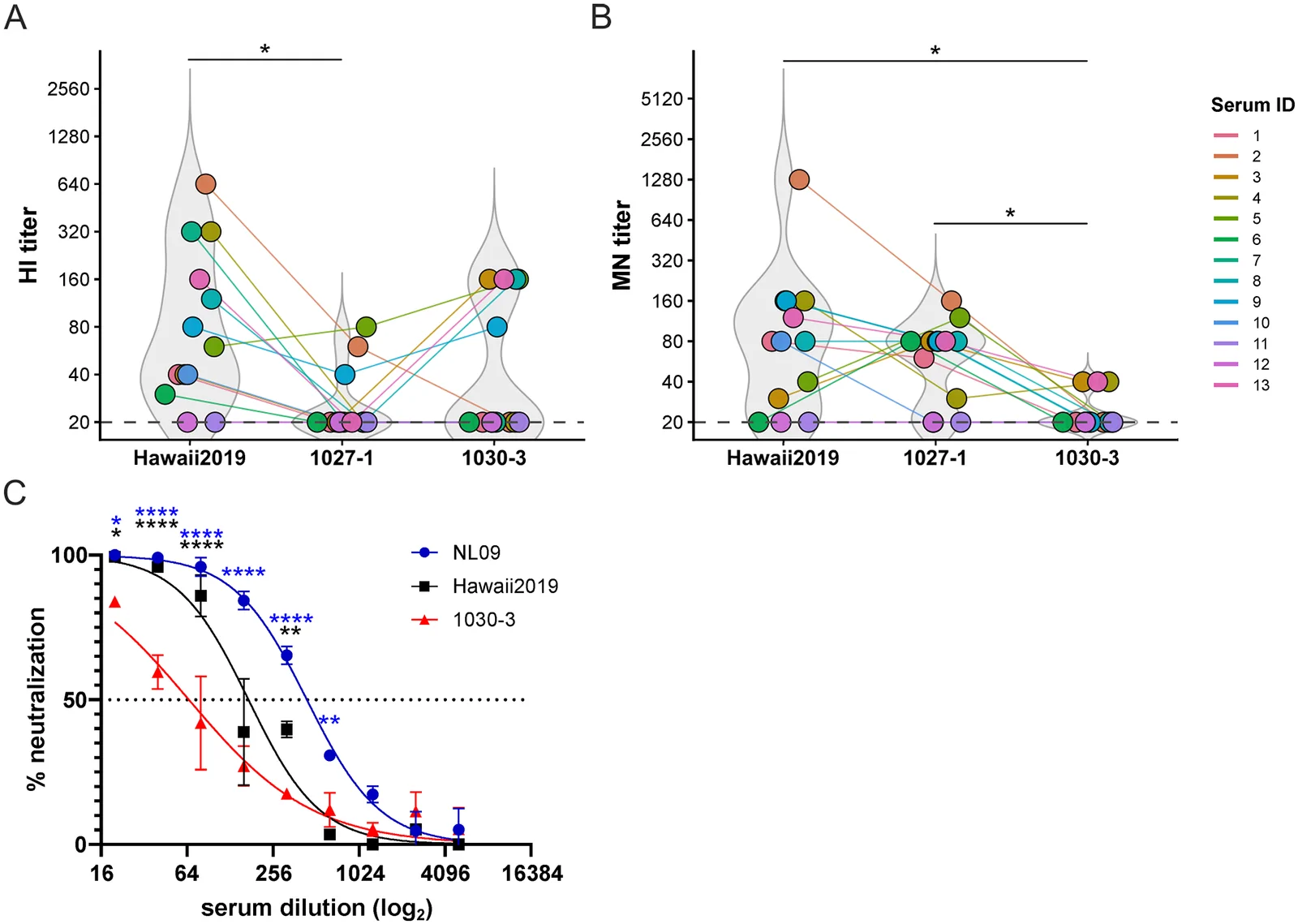
Reduced antibody recognition of A/swine/Korea/1030-3/2023 (1030-3) by human sera and H1N1 vaccine antiserum. **(A)** Hemagglutination inhibition (HI) titers of human sera (n = 13) against A/Hawaii/70/2019 (Hawaii2019), A/swine/Korea/1027-1/2023 (1027-1), and 1030-3. Lines connect titers obtained from the same individual serum sample. The dashed line indicates the assay limit of detection (HI titer = 20). **(B)** Microneutralization (MN) titers of the same human serum panel against Hawaii2019, 1027-1, and 1030-3. Lines connect titers obtained from the same individual serum sample. The dashed line indicates the assay limit of detection (MN titer = 20). Pairwise comparisons for HI and MN titers were performed using paired Wilcoxon signed-rank tests with Holm adjustment for multiple comparisons. **(C)** Neutralization of NL09, Hawaii2019, and 1030-3 by reference antiserum against A/Victoria/4897/2022-like H1N1 HA, measured by plaque-reduction neutralization test. Percent neutralization was calculated relative to virus-only controls. Data are presented as mean ± SEM of duplicate measurements. The dotted horizontal line indicates 50% neutralization. Blue and black asterisks indicate comparisons of 1030-3 with NL09 and Hawaii2019, respectively. Statistical significance was determined by two-way ANOVA followed by Dunnett’s multiple-comparison test. *p < 0.05, **p < 0.01, ***p < 0.001, ****p < 0.0001.

Microneutralization assays further characterized the serological profile of 1030-3. MN titers against 1030-3 were significantly lower than those against both Hawaii2019 and the endemic Korean H1N2 comparator 1027-1 (both adjusted p = 0.0224; Fig 7B). Most sera showed neutralizing titers at or near the assay detection limit against 1030-3, whereas broader neutralizing activity was observed against Hawaii2019 and, to a lesser extent, 1027-1. Thus, despite the heterogeneous HI responses, the MN assay demonstrated reduced neutralizing activity against 1030-3 relative to both comparator viruses.

To further evaluate antibody recognition elicited by a recent human H1N1 vaccine antigen, reference antiserum against A/Victoria/4897/2022-like H1N1 HA was tested against 1030-3, NL09, and Hawaii2019 by plaque-reduction neutralization assay. The antiserum showed lower neutralizing activity against 1030-3 than against either NL09 or Hawaii2019 across the tested dilutions (Fig 7C). Together with the human serum MN data, these findings further support reduced neutralizing antibody recognition of 1030-3 relative to human pdm09 H1N1 viruses.

## Discussion

This study describes the detection and characterization of a novel H1N2 swIAV reassortant, A/swine/Korea/1030-3/2023, identified during nationwide surveillance in South Korea in 2023. Genomic analysis showed that 1030-3 harbors a pdm09-derived backbone closely related to European pdm09 swine variants, including the Danish zoonotic strain A/Denmark/1/2021 [16], whereas its N2 segment belongs to the endemic Korean CS 1A.3.3.3 lineage (Fig 2B and S1 Fig). This genomic constellation distinguishes 1030-3 from the closely related European H1N1 viruses and is consistent with reassortment between a European-associated pdm09 swine-virus backbone and the endemic Korean swIAV gene pool. These findings extend previous evidence that Korean swIAV diversity has been shaped by repeated lineage introductions, human-to-swine transmission of pdm09 viruses, and reassortment among co-circulating swine lineages [17,20].

The time-scaled phylogeny, discrete phylogeographic reconstruction, tMRCA estimates, and live-pig import records provide convergent evidence linking 1030-3 to a European-associated pdm09 swine lineage and are consistent with introduction in the context of international pig movement (Fig 2). However, these analyses do not establish a direct transmission route from Denmark to the source farm. Farm-level records documenting the origin and movement history of the gilt from which 1030-3 was isolated were unavailable, and alternative introduction pathways, including undetected circulation through intermediate countries, cannot be excluded. Recent surveillance studies of H1 swIAVs in China have predominantly identified G4 Eurasian avian-like H1N1 viruses and diverse reassortant H1 viruses, with no reported H1 viruses closely related to the European-associated pdm09 swine lineage identified in the present study [21–23]. Nevertheless, the absence of such viruses from available surveillance datasets does not exclude their undetected circulation in China or other Asian countries, given differences in surveillance intensity and incomplete regional sequence availability. Moreover, because comparable nationwide surveillance data after 2023 were not available for the present analysis, we cannot determine whether this genotype became established in South Korean swine, represented a transient detection, or subsequently underwent additional reassortment. Continued nationwide genomic surveillance will therefore be required to determine the persistence and longer-term evolutionary trajectory of related viruses, while additional movement records and regional sequence data would be needed to further clarify the introduction pathway. Collectively, our findings highlight the importance of international livestock movement as a potential pathway connecting geographically separated swIAV gene pools and facilitating reassortment, while also illustrating the difficulty of reconstructing the precise route of individual introductions from genomic and epidemiological data alone [3,11].

The public health relevance of such reassortants depends on multiple viral and host factors. Risk-assessment frameworks for emerging influenza A viruses emphasize that no single phenotype is sufficient to define pandemic risk; rather, genetic relatedness, receptor specificity, replication competence in mammalian cells, transmissibility, antigenic relatedness to human viruses, population immunity, and antiviral susceptibility must be considered together [24,25]. In polarized Calu-3 cells cultured at an air–liquid interface, a model that preserves key physiological features such as tight junction integrity, mucus secretion, and human-type receptor expression patterns [26–28], 1030-3 replicated efficiently, demonstrating competence for replication in a human bronchial epithelial cell model (Fig 3). The endemic Korean H1N2 swIAV 1027-1 also replicated efficiently in this model, indicating that robust Calu-3 replication is not unique to 1030-3. Nevertheless, the presence of this phenotype in a newly detected European-associated pdm09-derived reassortant remains relevant when considered together with its distinct genomic constellation and efficient transmission in pigs.

Both 1030-3 and 1027-1 preferentially bound α2,6-linked sialic acids, confirming that this receptor preference is shared among swine-adapted viruses rather than being unique to 1030-3 (Fig 3B) [23]. The HA fusion thresholds of 1030-3 and 1027-1 were pH 5.7 and pH 5.6, respectively, compared with pH 5.3 for NL09 (Fig 3C), indicating lower HA acid stability for both swIAVs than for the early pdm09 reference virus. Although the contribution of this difference to transmission cannot be determined from the present experiments, the combined phenotype of efficient replication in human airway epithelial cells, α2,6-linked receptor preference, and reduced neutralization by contemporary human sera supports continued evaluation of 1030-3 at the swine–human interface.

In the mouse model, 1030-3 caused moderate body weight loss and replication in the respiratory tract, but it did not show enhanced pathogenicity compared with the early pdm09 reference strain NL09 (Fig 4A-4C). Tissue viral titers were generally lower in 1030-3-infected mice, particularly at 5 dpi, suggesting that 1030-3 remains replication competent in a mammalian respiratory tract but is not more virulent than NL09 in this model. Human seasonal A(H1N1)pdm09 viruses isolated between 2016 and 2019 have been reported to show reduced pathogenicity in mice relative to prototypic pandemic strain A/California/04/2009 [19]. Although direct evolutionary comparisons are limited, the detectable replication and weight-loss phenotype of 1030-3 is consistent with its divergence along a swine-associated, rather than contemporary human seasonal, evolutionary pathway. This interpretation is also supported by sequence-based antigenic cartography, which placed 1030-3 within the European pdm09 swine-virus cluster and apart from early and recent human pdm09 strains (Fig 1D), consistent with a distinct swine-associated evolutionary pathway [14,17].

In the guinea pigs, 1030-3 transmitted under direct-contact conditions but showed no detectable aerosol transmission (Fig 4D and 4E). Its HA fusion threshold was higher than that of NL09, indicating lower acid stability, a property that has been associated with reduced airborne transmissibility in other influenza virus systems [29]. However, the contribution of HA acid stability to the transmission phenotype of 1030-3 cannot be determined from the present study, and additional viral and host determinants are likely involved. Moreover, because an endemic Korean swIAV comparator was not included in the guinea pig experiment, these data do not establish whether the transmission phenotype of 1030-3 differs from that of other swine-adapted viruses.

In pigs, the natural host, both Korean endemic H1N2 swIAV 1027-1 and European-associated pdm09 1030-3 replicated and transmitted efficiently, producing substantial nasal shedding in directly inoculated animals and sustained shedding following contact exposure (Fig 5). These phenotypes are particularly important because sustained replication and transmission in pigs provide opportunities for further reassortment and adaptive evolution [14]. Infectious virus was detected in all 1030-3 contact pigs by 1 dpc, and contact pigs exposed to 1030-3 showed the highest cumulative shedding burden over the observation period. This burden was significantly greater than that observed in the NL09 group and numerically greater than that in the 1027-1 group. Such efficient replication and transmission in pigs may increase opportunities for onward spread, reassortment, and additional adaptation, although the persistence of this genotype in South Korean swine after 2023 remains unknown.

Comparative pathology and immune analyses revealed outcome-dependent differences among the three viruses at 5 dpi (Fig 6). Although 1030-3 produced high viral burdens throughout the respiratory tract, particularly in BALF, it did not induce uniformly greater pathology than 1027-1, which showed numerically greater macroscopic lung lesion scores. Cytokine and BAL-cell profiles also differed among strains, but these findings should be interpreted as descriptive and exploratory. IL-12p40 concentrations were significantly increased in 1027-1-infected pigs, whereas 1030-3 infection was associated with a significant increase in CD4 ⁺ αβ T-cell frequency. Other changes, including differences in alveolar macrophages, total αβ T cells, and CD8 ⁺ αβ T cells, were numerical trends rather than statistically supported effects. Because these measurements were obtained at a single time point from relatively small groups and were not accompanied by functional validation, they do not establish an immunological mechanism underlying the replication or transmission phenotype of 1030-3.

Several amino acid substitutions in 1030-3 remain of interest as candidate molecular determinants (S4 Fig). The PA substitutions N321K and I330V have been associated with enhanced polymerase activity and pathogenicity in swine influenza reassortants [30], whereas NS1 I123V substitution has been linked to restored NS1-mediated host-shutoff activity in pdm09 viruses [31]. However, their effects are likely to depend on the broader genetic background, and reverse-genetics studies will be required to determine whether these substitutions contribute to the phenotype of 1030-3.

The serological data further support the antigenic distinction of 1030-3 from human pdm09 H1N1 viruses (Fig 7). HI responses varied substantially among individual sera, and HI titers against 1030-3 were not significantly different from those against Hawaii2019, indicating heterogeneous HA-directed antibody recognition within the serum panel examined. In contrast, MN assays revealed a clearer difference in functional antibody activity: neutralizing titers against 1030-3 were significantly lower than those against both Hawaii2019 and the endemic Korean H1N2 swIAV 1027-1. This distinction is particularly notable because several in vitro and swine phenotypes were shared between 1030-3 and 1027-1, whereas their susceptibility to neutralization by the same human sera differed. Consistently, antiserum raised against a recent H1N1 vaccine antigen showed lower neutralizing activity against 1030-3 than against either early or contemporary human pdm09 H1N1 strains. Together, these findings are consistent with the sequence-based antigenic separation of 1030-3 from human pdm09 viruses (Fig 1D) [14], and suggest reduced neutralization of 1030-3 by the sera examined. However, the human serum panel examined does not support population-level conclusions regarding immunity, and broader serological studies will be needed to define the extent of cross-reactive immunity against this reassortant. In contrast, 1030-3 remained susceptible in vitro to baloxavir and oseltamivir (Fig 3D), indicating no detectable phenotypic resistance to these antiviral drugs under the conditions tested.

Collectively, our findings identify 1030-3 as a trade-associated H1N2 swIAV reassortant that combines a European-associated pdm09-derived backbone with an N2 segment from the endemic Korean swIAV gene pool. Its significance does not derive from a single uniquely enhanced phenotype. Rather, 1030-3 brings together a novel genomic constellation, efficient replication and direct-contact transmission in pigs, α2,6-linked receptor preference, replication in a human bronchial epithelial cell model, and reduced neutralization by contemporary human sera relative to both a contemporary human H1N1 virus and an endemic Korean H1N2 swIAV comparator. 1030-3 did not show enhanced pathogenicity relative to NL09 in mice and showed no detectable aerosol transmission in guinea pigs under the conditions tested. Thus, the combined data identify several features relevant to zoonotic-risk assessment but do not establish efficient human transmission or high pandemic potential. The efficient replication and transmission of 1030-3 in pigs nevertheless provide opportunities for onward spread, reassortment, and additional adaptation if related viruses persist in swine populations. Continued surveillance will therefore be important to determine whether this genotype becomes established, disappears, or generates additional reassortants. More broadly, this study supports an integrated, movement-aware approach to swIAV surveillance that combines animal-movement data, genomic sequencing, comparative phenotyping, and serological assessment to identify reassortants that warrant further investigation at the swine–human interface.

## Materials and methods

### Ethics statement

All mouse and guinea pig experiments were performed in an animal biosafety level 2 facility and were approved by the Institutional Animal Care and Use Committee (IACUC; WG-IACUC-2025-006). The pig experiment was conducted at the Korea Research Institute for Veterinary Biologics (KRIVB). All procedures were reviewed and approved by the IACUC (KVD-2024-010) and were conducted in accordance with the committee’s guidelines and regulations. Human serum samples were obtained from Kyungpook National University Hospital (KNUH) as anonymized clinical specimens with approval from the Institutional Review Board (KNUH IRB 2026-03-003).

### Sample collection, virus isolation and sequencing

Active surveillance for swIAV was conducted at 40 pig farms in South Korea among animals showing respiratory signs, as previously described [17]. Between October and December 2023, 40 nasal swabs and 5 pooled oral fluid samples were collected per farm across age groups, yielding 1600 nasal swabs and 200 pooled oral fluid samples. Viral RNA extraction, PCR detection, and swIAV isolation followed WOAH guidelines [32] and established protocols [17]. Isolates were confirmed by PCR with primer sets targeting the M, H1, H3, N1, and N2 genes [33]. Viral RNA was extracted, and all eight influenza segments were amplified by multisegment RT-PCR [34]. After purification, complete genomes were sequenced on the Illumina iSeq platform and de novo assembled with CLC Genomics Workbench, as described previously [17].

### Sequence dataset management

To investigate the epidemiology of H1Nx swIAV and human pdm09 viruses, we retrieved 44,668 HA sequences from GISAID (https://gisaid.org) in March 2024, including swIAV (n = 7032) and human pdm09 viruses (n = 37,636). Redundancy was reduced using CD-HIT [35] with region- and host-specific identity thresholds (S3 Table). An additional 21 sequences representing WHO-recommended pdm09 vaccine strains (n = 4) and lineage reference strains (n = 17) were included. The final dataset (n = 940) comprised sequences from South Korea (n = 61), Japan (n = 82), China (n = 133), Southeast Asia (n = 41), Europe (n = 394), and North America (n = 229). A full list of isolates, including GISAID IDs, subtypes, and HA clade assignments, is provided in S4 Table.

### Phylogenetic analysis

Full-length nucleotide sequences of all eight segments (PB2, PB1, PA, HA, NP, NA, M, NS) were aligned with MAFFT and analysed by maximum-likelihood (ML) phylogeny. Maximum-likelihood trees were reconstructed with RAxML-NG under a GTR + Γ substitution model with 1000 bootstrap replicates. Trees were visualized and annotated with the *ggtree* package in R, and genotypes were assigned according to lineage constellations.

To further investigate the European-origin pdm09 swine variant, H1 sequences from the 1A Classical swine lineage were analyzed in BEAST v1.10.4 under a GTR + Γ substitution model and an uncorrelated lognormal relaxed clock. Four independent MCMC chains (100 million generations each, sampled every 10,000 steps) were combined after a 10% burn-in. Convergence was assessed in Tracer v1.7.1 (ESS > 200). MCC trees were summarized in TreeAnnotator and visualized with ggtree.

### Sequence-based antigenic cartography

Antigenic distances among pdm09 HA genes were estimated using a sequence-based antigenic cartography approach [36]. Amino acid differences within the five HA epitopes (Sa, Sb, Ca1, Ca2, Cb) were quantified as epitope distances (EDs) and averaged to generate an antigenic distance (AD) matrix. Antigenic maps were constructed using multidimensional scaling, and clusters were inferred by hierarchical clustering. ADs were expressed in antigenic units (AU), with values <8.0 AU indicating potential antibody cross-recognition [36].

### Pig importation data, tMRCA, and phylogeographic inference

To provide context for the phylogenetic findings, official records of annual live pig imports into Korea during 1996–2024 were obtained from the Korean Animal and Plant Quarantine Agency (https://www.qia.go.kr/). Country-specific import data from major exporting nations are presented in the S1 Table.

To compare the timing of swIAV lineage emergence with live-pig importation patterns, mean times to the most recent common ancestor (tMRCAs) and 95% highest posterior density (HPD) intervals were extracted from time-scaled MCC trees for selected swIAV clades relevant to the Korean gene pool and the 1030-3 reassortant as previously described [17]. These included endemic Korean swIAV lineages implicated in reassortment or prior diversification, including CS 1A.3.3.3 [37,38], H3N2pM-like [39], and 1C.2.3 lineages [17,20], as well as the European pdm09 swine variant lineage (EUsw-pdm09, 1A.3.3.2) containing A/swine/Korea/1030-3/2023.

Spatial diffusion dynamics of European-origin pdm09 swine variants were inferred in BEAST v1.10.4 under a discrete-trait BSSVS framework, with country or region specified as discrete traits. MCC trees were summarized in TreeAnnotator, and phylogeographic reconstructions were performed with SPREAD3 v0.9.7. Viral diffusion patterns were visualized on geographic maps, with only strongly supported transitions (Bayes factor > 10) highlighted.

### Virus preparation and titration

A/swine/Korea/1030-3/2023 (H1N2, 1030-3) and A/swine/Korea/1027-1/2023 (H1N2, 1027-1) were propagated in embryonated chicken eggs, aliquoted, and stored at −80°C. The pdm09 reference strain A/Netherlands/602/2009 (H1N1, NL09) was generated by reverse genetics, as previously described [40] and subsequently amplified in MDCK cells. A/Hawaii/70/2019 (H1N1, Hawaii2019) was obtained from National Culture Collection for pathogens (NCCP), and propagated in embryonated chicken eggs, aliquoted, and stored at −80°C. Viral titers were determined by plaque assay in MDCK cells.

### Solid-phase binding assay

Viral receptor-binding specificity was assessed using a fetuin-based solid-phase binding assay, as previously described [41,42]. Biotinylated sialylglycopolymers representing α2,3-linked (3′SLN) and α2,6-linked (6′SLN) receptors were used to determine binding profiles. Bound glycans were detected with streptavidin–HRP and TMB substrate, and absorbance was measured at 450 nm.

### Replication kinetics in an air-liquid interface (ALI) model

Replication kinetics of the 1030-3, 1027-1, and NL09 viruses were evaluated in polarized Calu-3 cells cultured under ALI conditions, as described previously [26]. Briefly, Calu-3 cells were seeded at 5 × 10^5^ cells/well onto 12 mm Transwell inserts with 0.4 µm pore polyester membranes and cultured until confluent. After the apical medium was removed, cells were further differentiated under ALI conditions using basolateral medium supplemented with 2% FBS. Prior to infection, Calu-3 cells with transepithelial electrical resistance (TEER) > 600 Ω were inoculated at a multiplicity of infection (MOI) of 0.001. Apical washes were collected at multiple time points post-infection by adding 100 μL of PBS to the apical chamber and incubating for 30 min at 37°C. Samples were harvested, stored at −80 °C, and viral titers were determined by plaque assay.

### Syncytia formation assay

Syncytia induction was assessed in Vero cells infected at a high MOI under conditions adapted from established methods [43]. After 24 h of incubation at 37°C, infected cells were treated with 5 μg/mL TPCK-treated trypsin in DMEM and exposed to pH-adjusted PBS for 15 min at 37°C. Cells were then fixed with methanol and stained with 0.02% crystal violet. The fusion threshold was defined as the highest pH at which syncytium formation was consistently observed across replicate wells.

### Antiviral susceptibility

The plaque reduction neutralization test (PRNT) for baloxavir was performed as previously described [44]. MDCK cells were seeded in 12-well plates at a density of 5 × 10^5^ cells/well. Virus stocks were diluted to yield 100 PFU/well and incubated for 1 h at 37°C. The infected cells were then overlaid with medium containing low-melting agar and various concentrations of baloxavir. Plates were incubated for 72 h at 37°C, fixed with 4% formaldehyde, and stained with crystal violet. Neuraminidase inhibition (NI) by oseltamivir was measured with the NA-Fluor Influenza Neuraminidase Assay Kit (Invitrogen), following the manufacturer’s protocol. IC_50_ values were calculated from reduction curves for both assays.

### Pathogenicity in mice

Thirty-three 6-week-old female BALB/c mice were divided into three groups (mock, NL09, and 1030-3) and inoculated intranasally with 1 × 10^5^ PFU of the respective virus. Body weight was monitored daily for 14 days post-inoculation (dpi). Lungs and tracheas were harvested at 3 and 5 dpi for viral titration by plaque assay.

### Transmission in guinea pig

Female Hartley strain guinea pigs weighing 300–350 g were used for transmission studies. Animals were intranasally inoculated with 1 × 10^5^ PFU of virus in a total volume of 300 μL. At 24 h post-inoculation, naïve guinea pigs were introduced either into the same cage as inoculated animals (contact transmission) or housed in adjacent cages allowing airflow without direct contact (aerosol transmission), following established protocols [45]. Nasal wash samples were collected at 2, 4, 6, and 8 dpi using PBS as the collection fluid. Samples were aliquoted and stored at −80°C until further analysis. Viral titers in nasal wash samples were determined by plaque assay on MDCK cells.

### Pig experiment

Animal experiments were conducted in 28 four-week-old piglets obtained from swIAV- and PRRSV/PCV2-free herds. Sixteen piglets were divided into four inoculation groups (Mock, NL09, 1027-1, and 1030-3) and challenged intranasally with 3 × 10^7^ PFU of virus. To assess transmission, the remaining 12 contact piglets were separately introduced to each inoculation group (n = 4) at 2 dpi. Four inoculated pigs and four contact pigs were co-housed as a group within each virus treatment. Animals were not maintained as fixed 1:1 donor-contact pairs. Inoculated pigs were euthanized at 5 dpi and contact pigs at 14 days post-contact (dpc). Clinical monitoring, body weight measurement, viral load quantification by RT-qPCR, bronchoalveolar lavage (BAL) collection, BAL cell flow cytometry, and lung cytokine assays were performed according to established protocols [46,47]. Viral loads measured by RT-qPCR were converted from Ct values to PFU-equivalent titers using virus-specific standard curves generated from serial dilutions of NL09, 1027-1 and 1030-3 stocks with known infectious titers determined by plaque assay (S5 Fig). Macroscopic lung lesions were scored using the Halbur scoring system [48]. Briefly, the percentage of lung surface consolidation was visually assessed for each lobe from ventral and dorsal views. Individual lobe scores were then weighted according to the proportional contribution of each lobe to total lung volume and summed to generate a final score (maximum = 100).

### Human serum collection

Anonymized human sera were collected as clinical specimens (n = 13) with informed consent and approval from the Institutional Review Board of Kyungpook National University Hospital (KNUH IRB 2026-03-003). Sera were grouped according to seasonal vaccination status and documented influenza infection history: unvaccinated with recent confirmed infection (n = 4), vaccinated with recent confirmed infection (n = 4), vaccinated with no recorded infection (n = 4), and unvaccinated with no infection history (n = 1). Available donor metadata, including birth year, vaccination status and date, and documented influenza infection history and date, are provided in S2 Table.

### Hemagglutination inhibition assay

Human serum samples were treated with receptor-destroying enzyme (RDE II; Denka Seiken) and heat-inactivated at 56°C for 30 min before testing. Hemagglutination inhibition (HI) assays were performed against WHO-recommended pdm09 vaccine strain A/Hawaii/70/2019 (Hawaii2019), 1027-1, and 1030-3. Sera were serially two-fold diluted beginning at 1:20, mixed with 4 hemagglutination units (HAU) of each virus, and incubated for 1 h at 4°C. Guinea pig red blood cells (gRBCs; 1%) were then added, followed by incubation for 1 h at 4°C, after which hemagglutination was assessed. HI titers were defined as the reciprocal of the highest serum dilution that completely inhibited hemagglutination. All assays were performed in duplicate.

### Microneutralization assay

Microneutralization assays were performed using the same human serum panel against Hawaii2019, 1027-1, and 1030-3. RDE-treated and heat-inactivated sera were serially two-fold diluted from 1:20 and incubated with each virus at an MOI of 0.03 for 1 h at room temperature. MDCK cells were then added in infection medium containing 1 µg/mL TPCK-treated trypsin and incubated at 37°C in 5% CO_2_. Following incubation, culture supernatants were mixed with 1% guinea pig red blood cells, and residual virus replication was assessed by hemagglutination. The microneutralization (MN) titer was defined as the reciprocal of the highest serum dilution achieving ≥50% inhibition of virus-induced hemagglutination. All assays were performed in duplicate.

### Standard H1N1 antiserum plaque-reduction neutralization assay

Standard H1N1 antiserum against A/Victoria/4897/2022-like (H1N1) HA (NIBSC) was pretreated, heat-inactivated, and serially diluted as described above. Diluted antiserum was incubated with 100 PFU of 1030-3, NL09, or Hawaii2019 for 1 h at 37°C, and virus–serum mixtures were inoculated onto MDCK cell monolayers. After 1 h adsorption, cells were overlaid with 1% agarose in MEM, and plaques were counted 72–96 h post-infection. Percent neutralization was calculated relative to virus-only controls, and dose-response curves were used to calculate PRNT_50_/IC_50_ values. All assays were performed in duplicate.

## Supporting information

S1 Fig Maximum-likelihood (ML) phylogenetic tree of non-HA segments. (A) PB2, (B) PB1, (C) PA, (D) NP, (E) NA-N1, (F) NA-N2, (G) M, (H) NS.
(PDF)

S2 Fig Lung cytokine responses in directly infected pigs. (PDF)

S3 Fig Bronchoalveolar lavage immune cell populations in directly infected pigs. Gating strategies for (A) myeloid cell and (B) lymphoid cell populations. (C) Frequencies of myeloid cell populations in BAL cells at 5 dpi, including live myeloid cells, MHC II⁺ cells, alveolar macrophages, monocyte-derived dendritic cells (moDC), cDC1, and cDC2. Alveolar macrophages, moDC, cDC1, and cDC2 were defined as MHC II ⁺ CD163 ⁺ CD172α ⁺ , MHC II ⁺ CD163^low^CD172α ⁺ , MHC II ⁺ CD163 ⁻ CD172α ⁻ , and MHC II ⁺ CD163 ⁻ CD172α⁺ cells, respectively. (D) Frequencies of lymphocyte populations in BAL cells, including potential lymphocytes, CD3 ⁺ γδ T cells, CD8 ⁺ γδ T cells, CD8 ⁻ γδ T cells, CD3 ⁺ αβ T cells, CD4 ⁺ αβ T cells, and CD8 ⁺ αβ T cells. Frequencies are expressed among single cells. Each dot represents an individual animal, and bars indicate mean ± SEM.
(PDF)

S4 Fig Amino acid alignments. (A) PA and (B) NS1 proteins from A/Netherlands/602/2009 (NL09), A/swine/Korea/1027-1 (1027-1), and A/swine/Korea/1030-3/2023 (1030-3). Dots indicate amino acids identical to those of NL09. Selected substitutions are highlighted in red, with PA N321K, PA I330V, and NS1 I123V annotated as molecular features potentially associated with the phenotypic characteristics of 1030-3.
(PDF)

S5 Fig Standard curves for conversion of RT-qPCR Ct values to PFU-equivalent viral loads. Standard curves generated from serial dilutions of virus stocks with known infectious titers for (A) NL09, (B) 1027-1, and (C) 1030-3. Linear regression equations and R^2^ values are shown in each panel.
(PDF)

S1 Table Country-specific pig importation data from major exporting nations during 1996–2024. (PDF)

S2 Table Clinical and vaccination metadata of human serum samples. (PDF)

S3 Table Summary of HA sequence datasets before and after CD-HIT clustering by host and geographic origin. (PDF)

S4 Table Complete list of H1 influenza A virus isolates with GISAID accessions, HA clade assignments, and metadata. (PDF)

S1 Data Source data underlying all figures and statistical analyses. (XLSX)
