# Supplementary material for: Pathogenicity and zoonotic potential of a novel pdm09-lineage H1N2 swine influenza A virus spatiotemporally linked to transboundary pig trade
Source: PLoS Pathog. 2026 Nov 3;22(11):e1014779. doi: 10.1371/journal.ppat.1014779 (PMC13743032; doi:10.1371/journal.ppat.1014779)
Supplement: Supplementary file 1 [file ppat.1014779.s001.pdf]

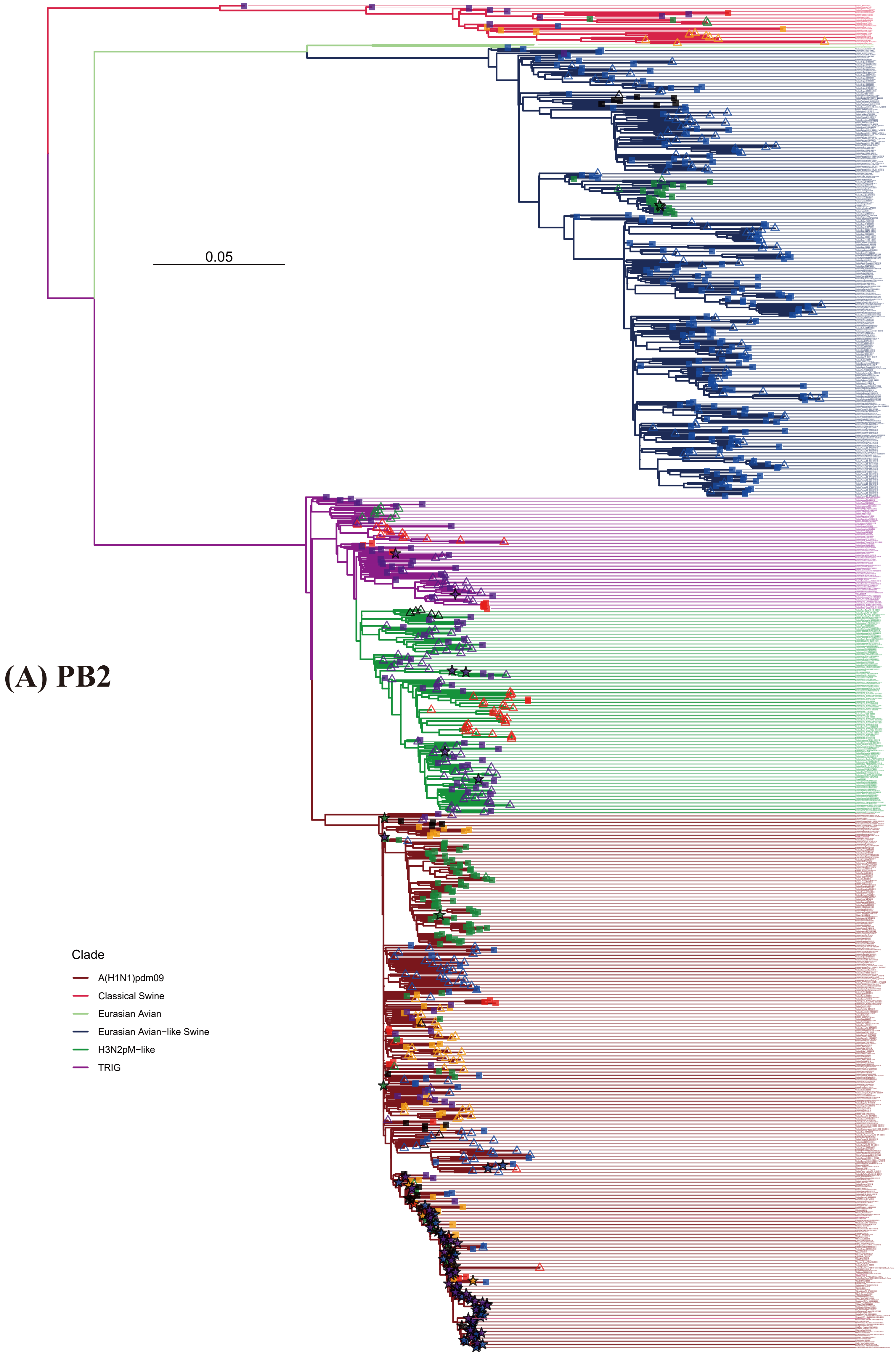

(A) PB2
0.05
Clade
A(H1N1)pdm09
Classical Swine
Eurasian Avian
Eurasian Avian−like Swine
H3N2pM−like
TRIG

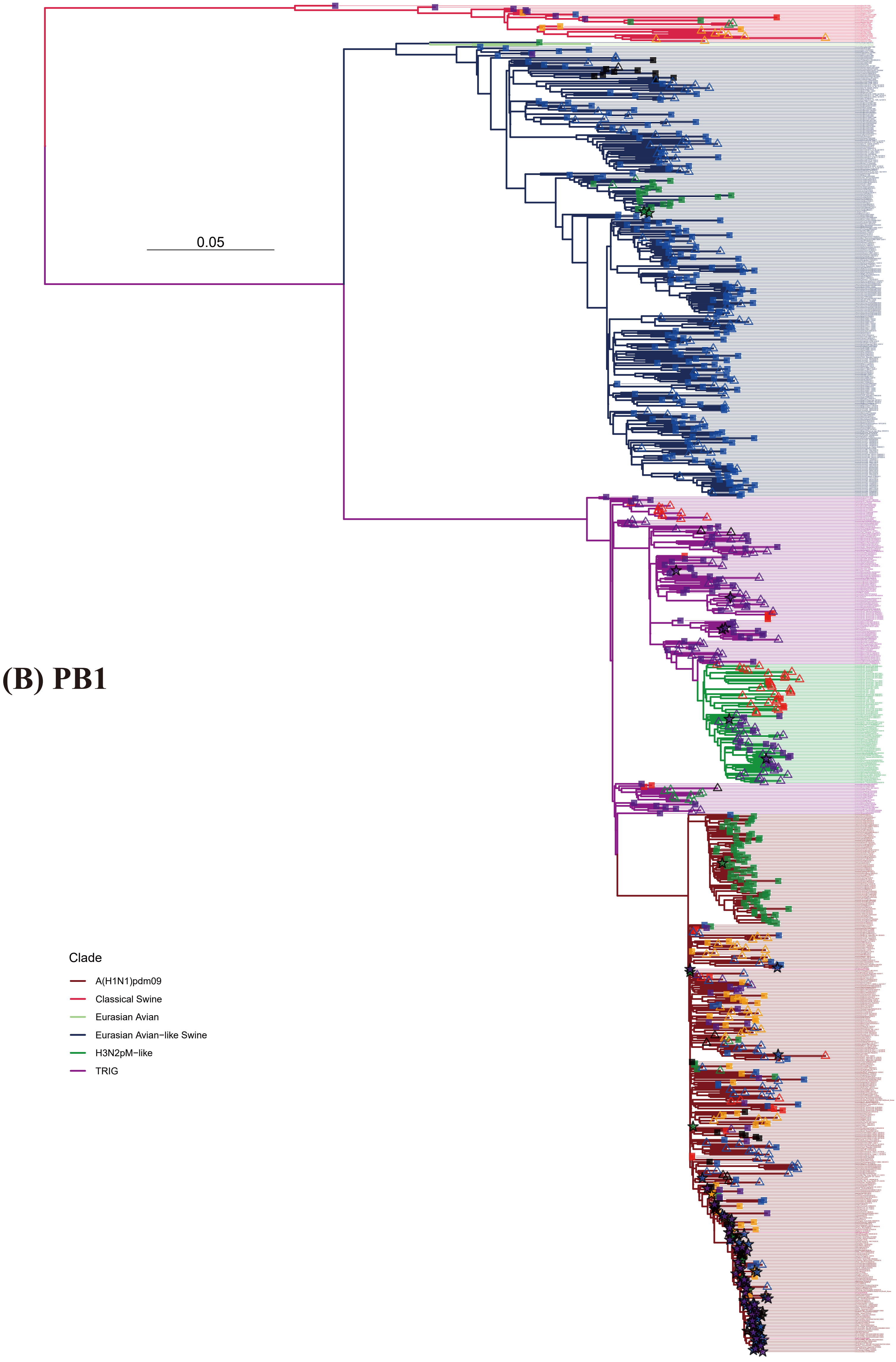
0.05
(B) PB1
Clade
A(H1N1)pdm09
Classical Swine
Eurasian Avian
Eurasian Avian−like Swine
H3N2pM−like
TRIG

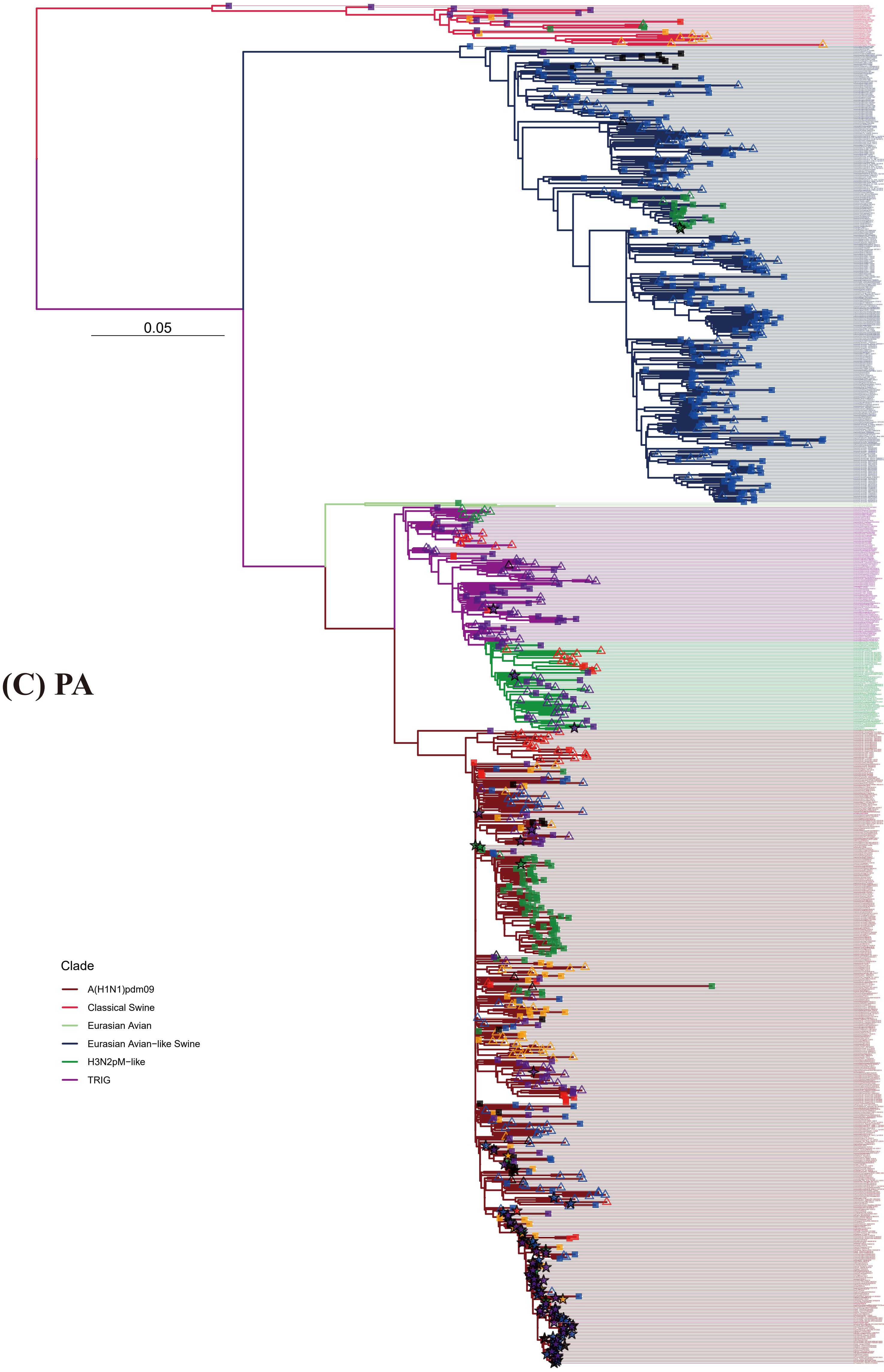

0.05
(C) PA
Clade
A(H1N1)pdm09
Classical Swine
Eurasian Avian
Eurasian Avian−like Swine
H3N2pM−like
TRIG

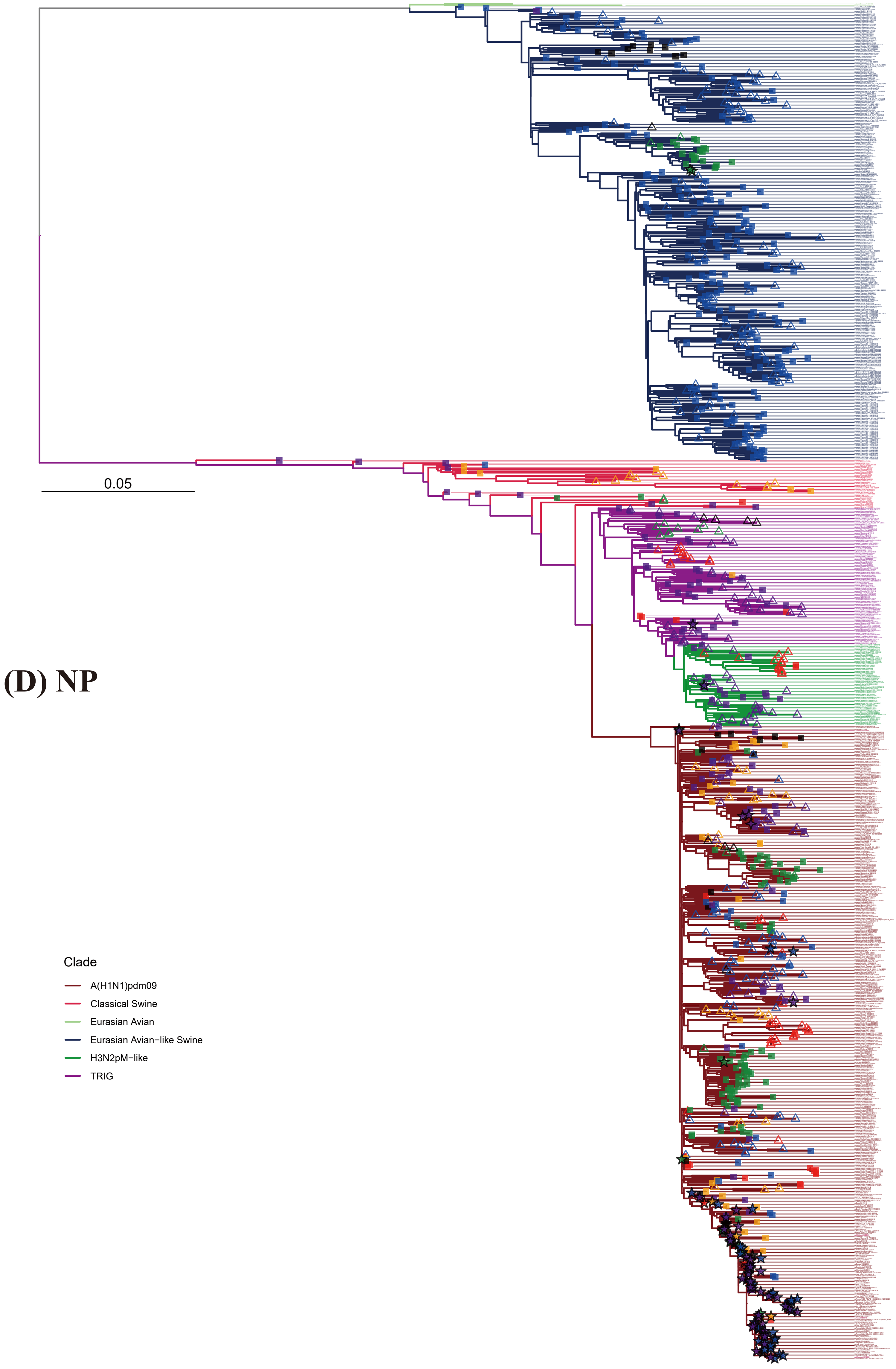
0.05
(D) NP
Clade
A(H1N1)pdm09
Classical Swine
Eurasian Avian
Eurasian Avian−like Swine
H3N2pM−like
TRIG

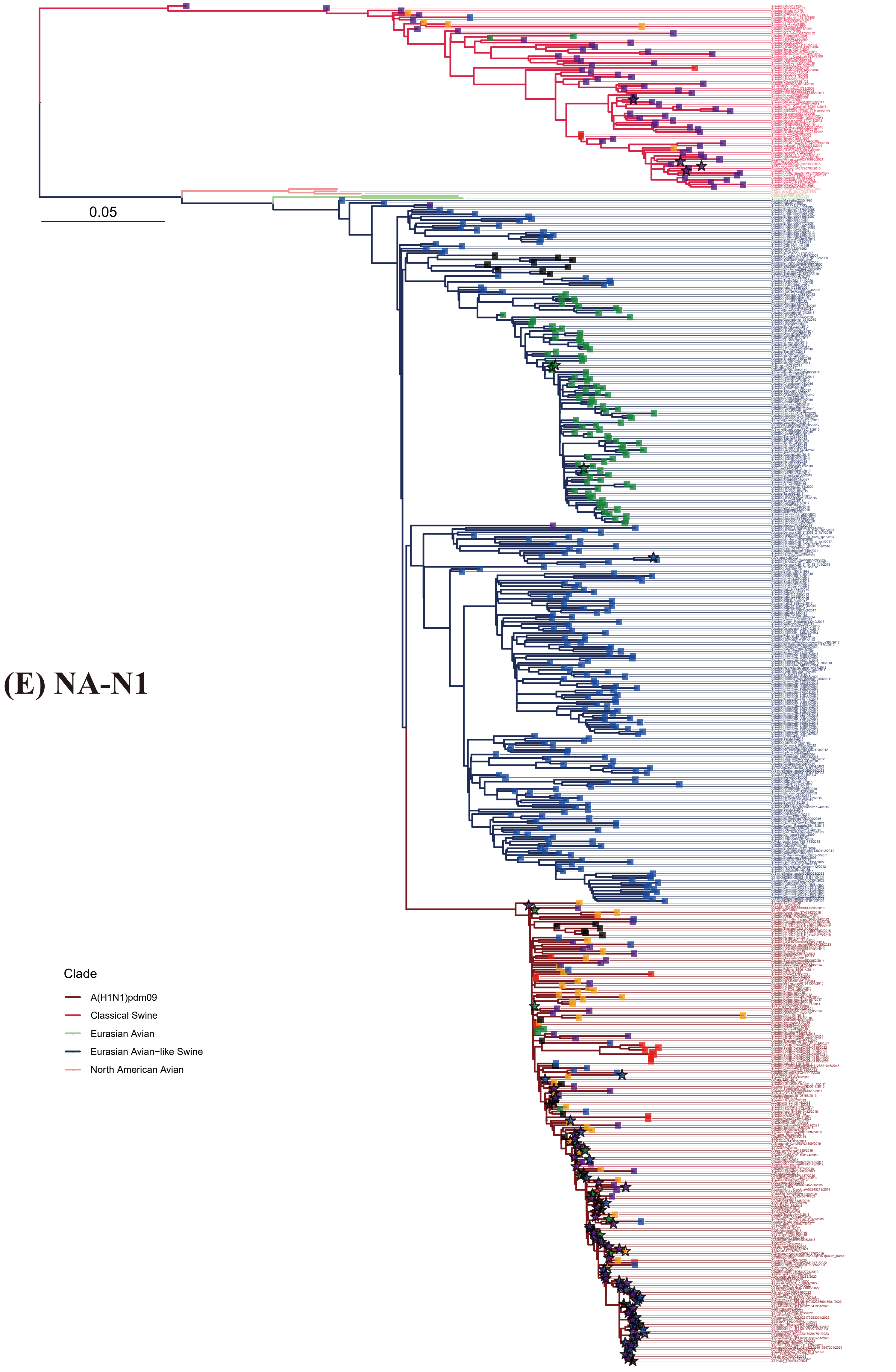
0.05
(E) NA-N1
Clade
A(H1N1)pdm09
Classical Swine
Eurasian Avian
Eurasian Avian−like Swine
North American Avian

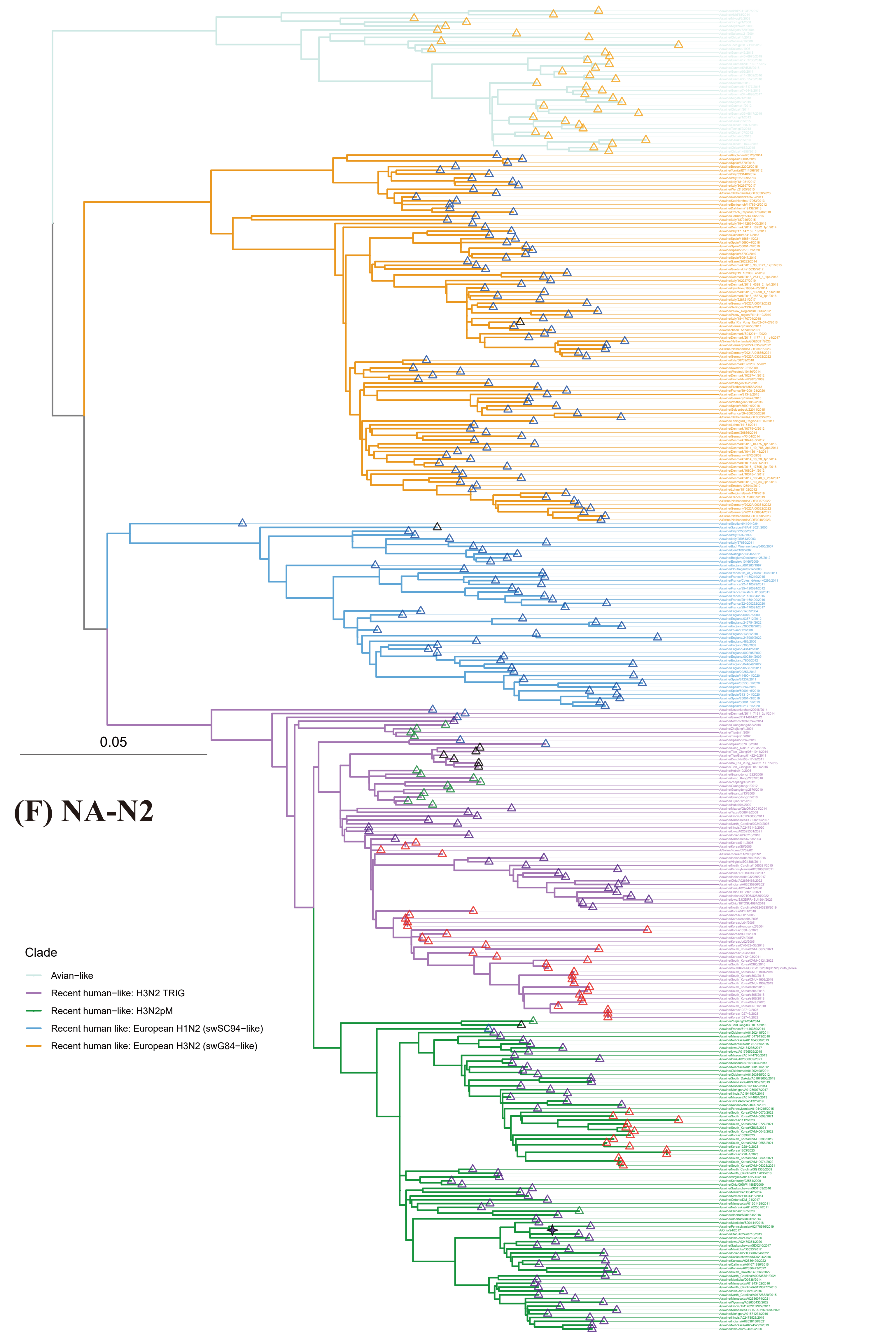
0.05
(F) NA-N2
Clade
Avian−like
Recent human−like: H3N2 TRIG
Recent human−like: H3N2pM
Recent human like: European H1N2 (swSC94−like)
Recent human like: European H3N2 (swG84−like)

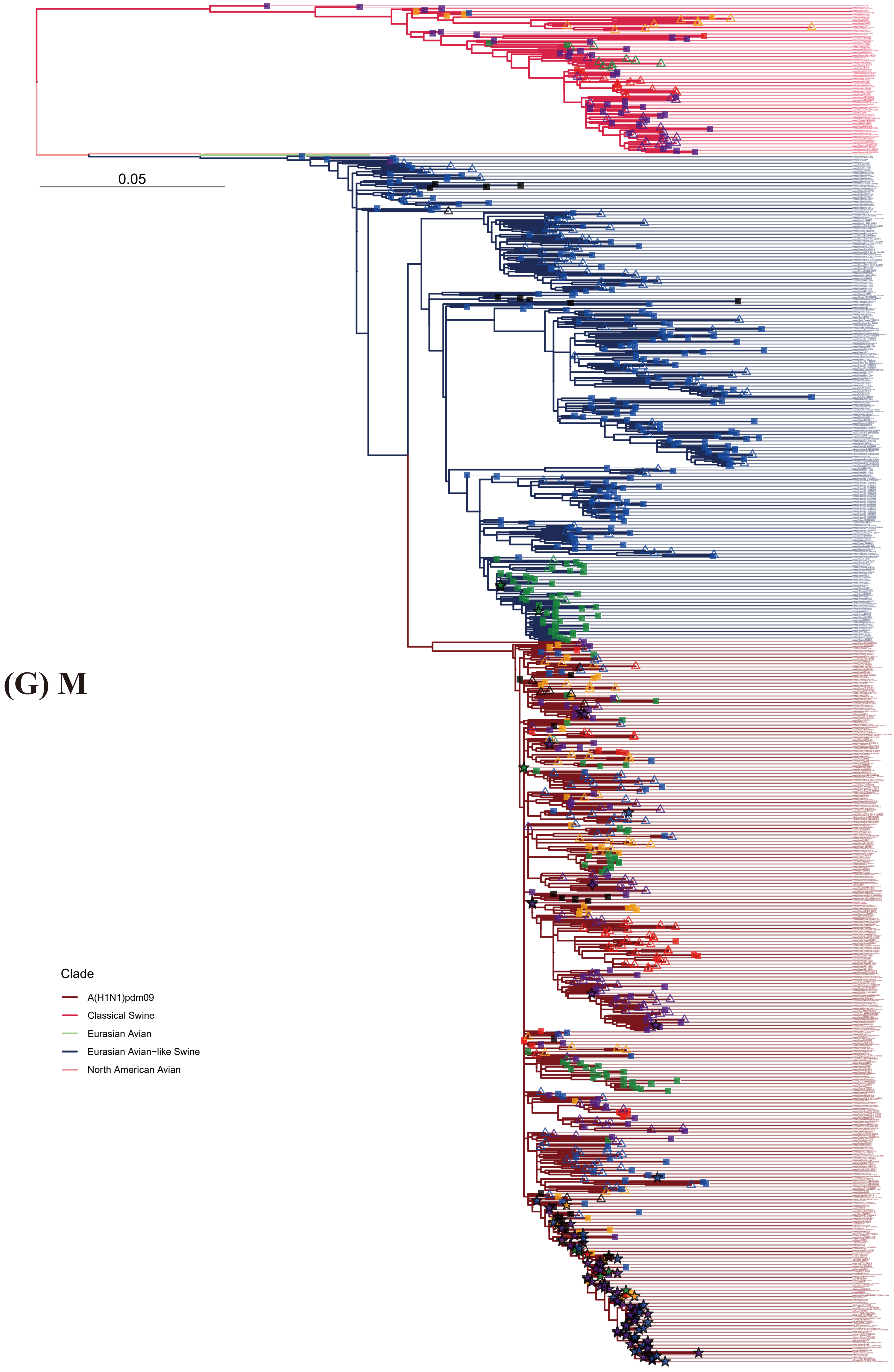
0.05
(G) M
Clade
A(H1N1)pdm09
Classical Swine
Eurasian Avian
Eurasian Avian-like Swine
North American Avian

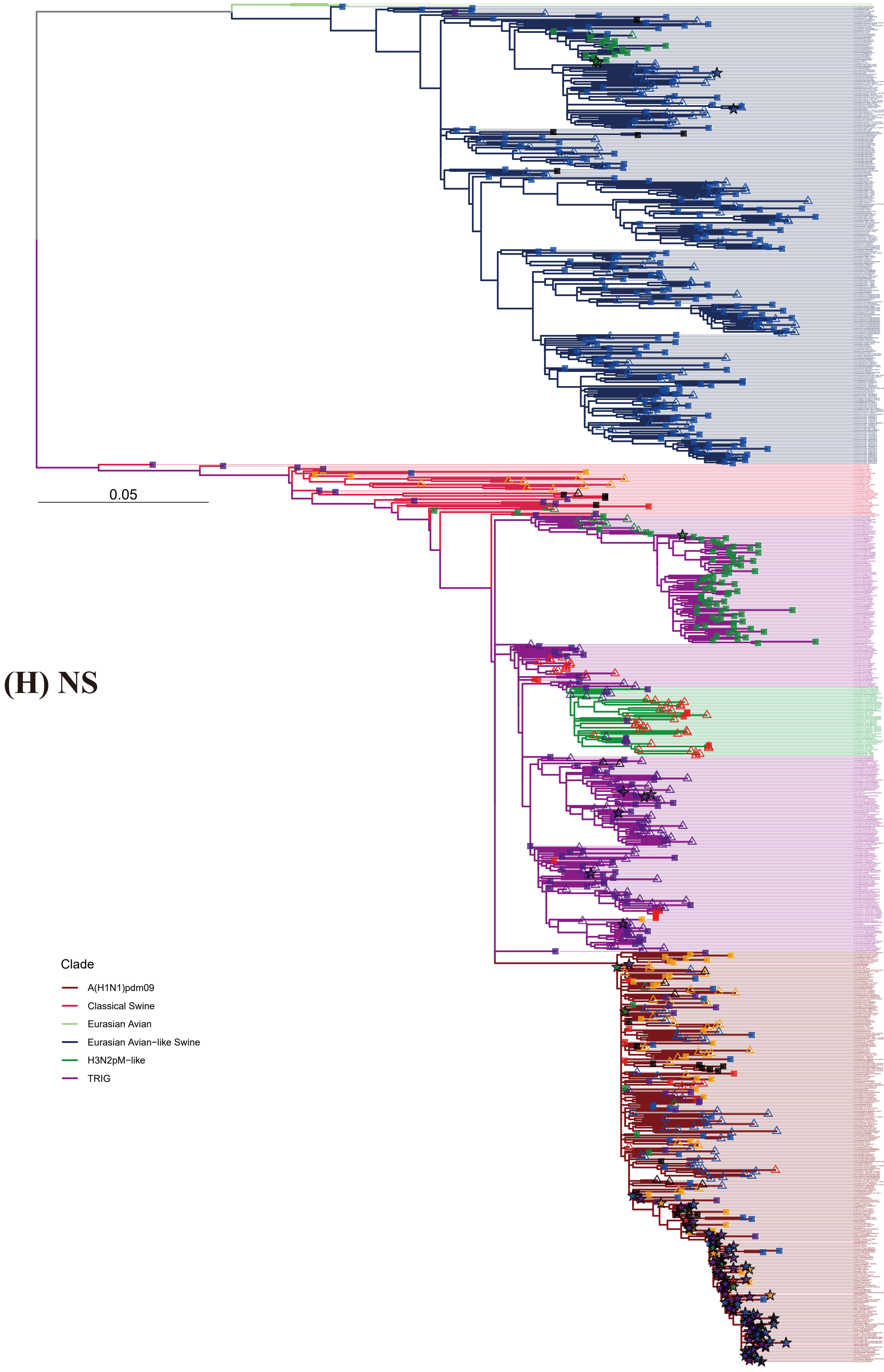

(H) NS
0.05
Clade
A(H1N1)pdm09
Classical Swine
Eurasian Avian
Eurasian Avian−like Swine
H3N2pM−like
TRIG

**Supplementary Figure S1.** Maximum-likelihood (ML) phylogenetic tree of non-HA segments including (A) PB2, (B) PB1, (C) PA, (D) NP, (E) NA-N1, (F) NA-N2, (G) M, (H) NS.
