# Supplementary material for: Pathogenicity and zoonotic potential of a novel pdm09-lineage H1N2 swine influenza A virus spatiotemporally linked to transboundary pig trade
Source: PLoS Pathog. 2026 Nov 3;22(11):e1014779. doi: 10.1371/journal.ppat.1014779 (PMC13743032; doi:10.1371/journal.ppat.1014779)
Supplement: Supplementary file 3 [file ppat.1014779.s003.pdf]

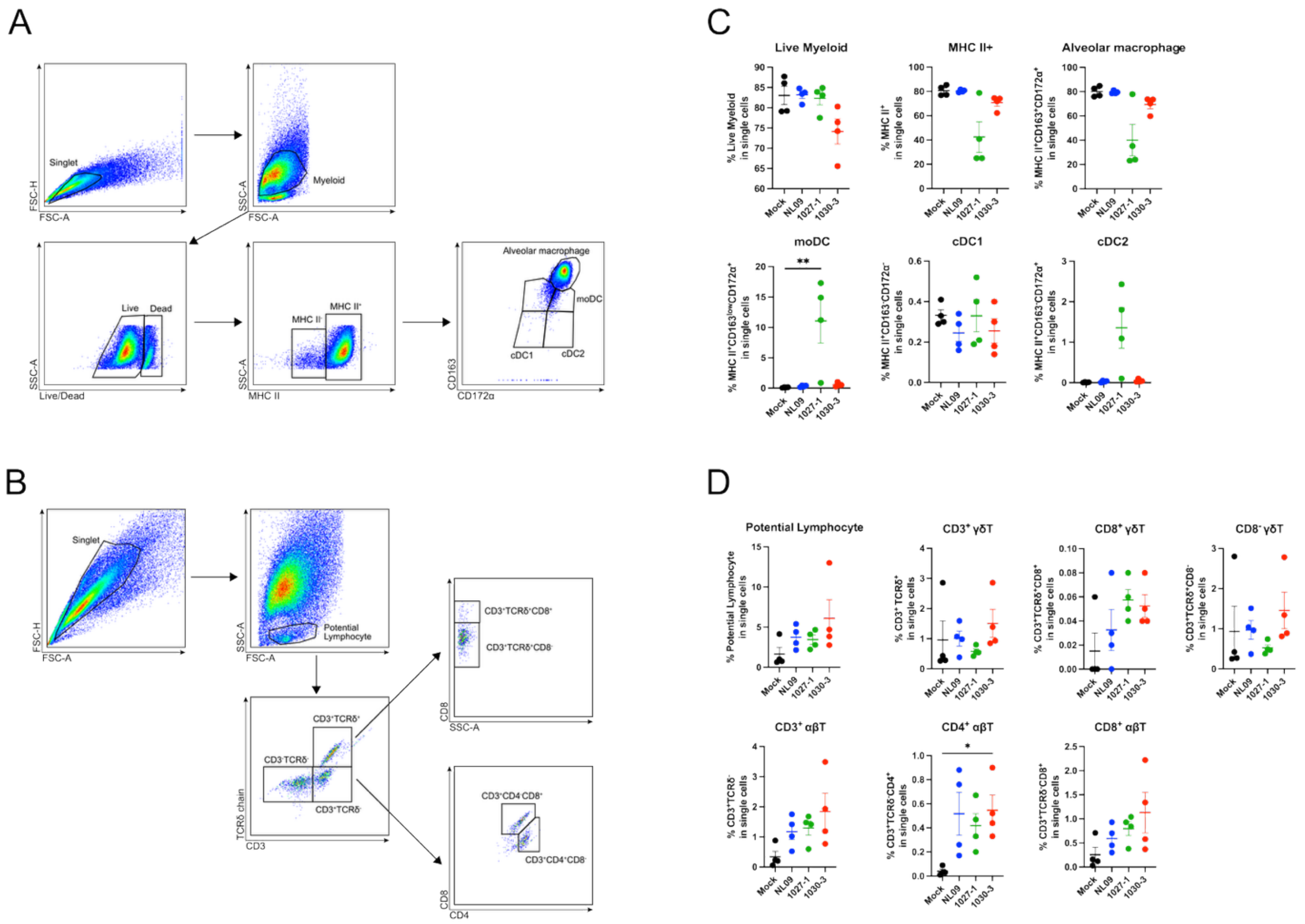

Supplementary Figure S3. Bronchoalveolar lavage immune cell populations in directly infected pigs. (A) Frequencies of myeloid cell populations in BAL cells at 5 dpi, including live myeloid cells, MHC II^{+} cells, alveolar macrophages, monocyte-derived dendritic cells (moDC), cDC1, and cDC2. Alveolar macrophages, moDC, cDC1, and cDC2 were defined as MHC II^{+}CD163^{+}CD172α^{+}, MHC II^{+}CD163^{low}CD172α^{+}, MHC II^{+}CD163^{-}CD172α^{-}, and MHC II^{+}CD163^{-}CD172α^{+} cells, respectively. (B) Frequencies of lymphocyte populations in BAL cells, including potential lymphocytes, CD3^{+}γδ T cells, CD8^{+}γδ T cells, CD8^{-}γδ T cells, CD3^{+}αβ T cells, CD4^{+}αβ T cells, and CD8^{+}αβ T cells. Frequencies are expressed among single cells. Each dot represents an individual animal, and bars indicate mean ± SEM.
