# Supplementary material for: Pathogenicity and zoonotic potential of a novel pdm09-lineage H1N2 swine influenza A virus spatiotemporally linked to transboundary pig trade
Source: PLoS Pathog. 2026 Nov 3;22(11):e1014779. doi: 10.1371/journal.ppat.1014779 (PMC13743032; doi:10.1371/journal.ppat.1014779)
Supplement: Supplementary file 4 [file ppat.1014779.s004.pdf]

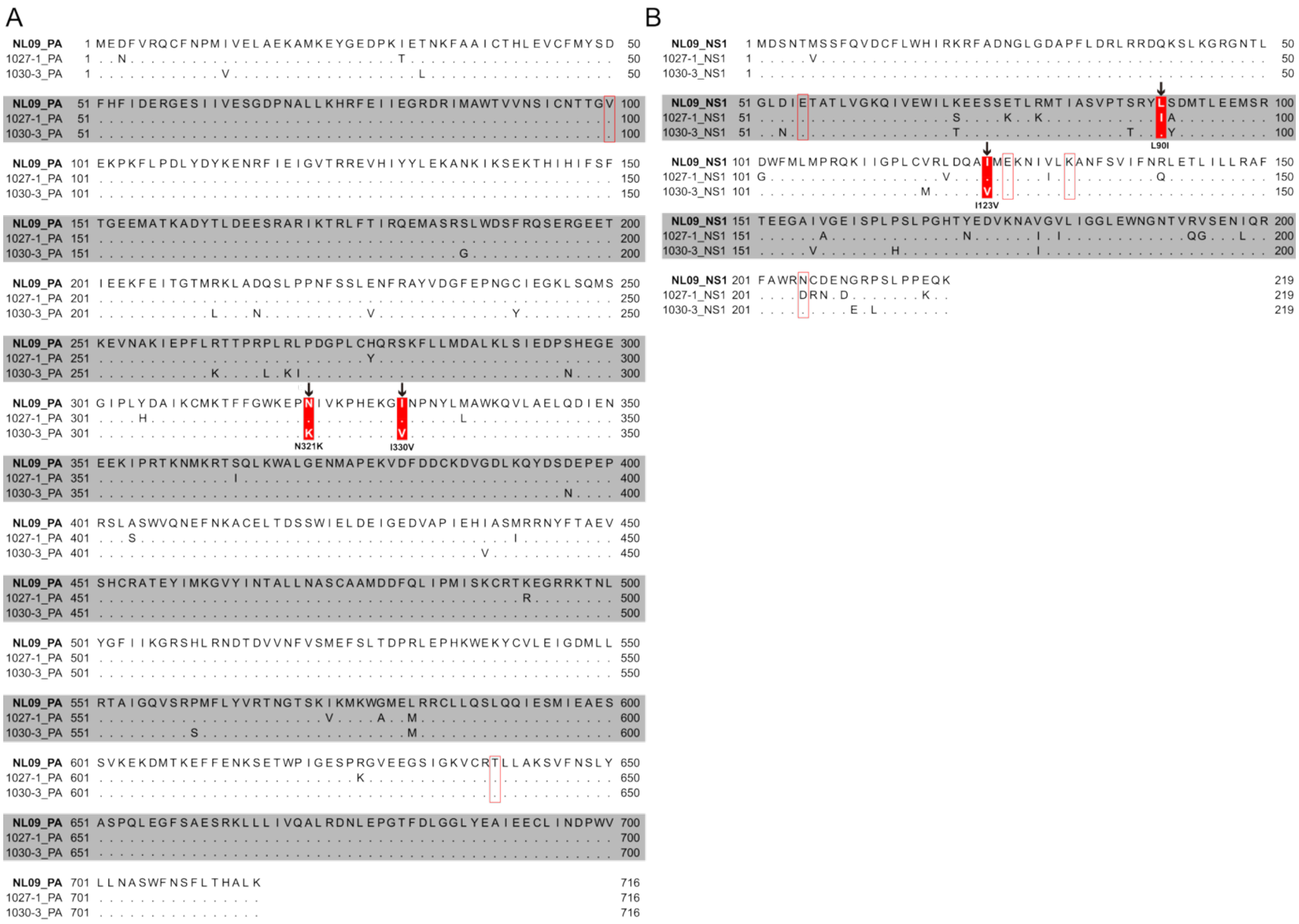

Supplementary Figure S4. Amino acid alignments of the (A) PA and (B) NS1 proteins from A/Netherlands/602/2009 (NL09), A/swine/Korea/1027-1/2023 and A/swine/Korea/1030-3/2023 (1030-3). Dots indicate amino acids identical to those of NL09. Selected substitutions are highlighted in red, with PA N321K, PA I330V, and NS1 I123V annotated as molecular features potentially associated with the high-fitness phenotype of 1030-3.
