# Supplementary material for: Pathogenicity and zoonotic potential of a novel pdm09-lineage H1N2 swine influenza A virus spatiotemporally linked to transboundary pig trade
Source: PLoS Pathog. 2026 Nov 3;22(11):e1014779. doi: 10.1371/journal.ppat.1014779 (PMC13743032; doi:10.1371/journal.ppat.1014779)
Supplement: Supplementary file 5 [file ppat.1014779.s005.pdf]

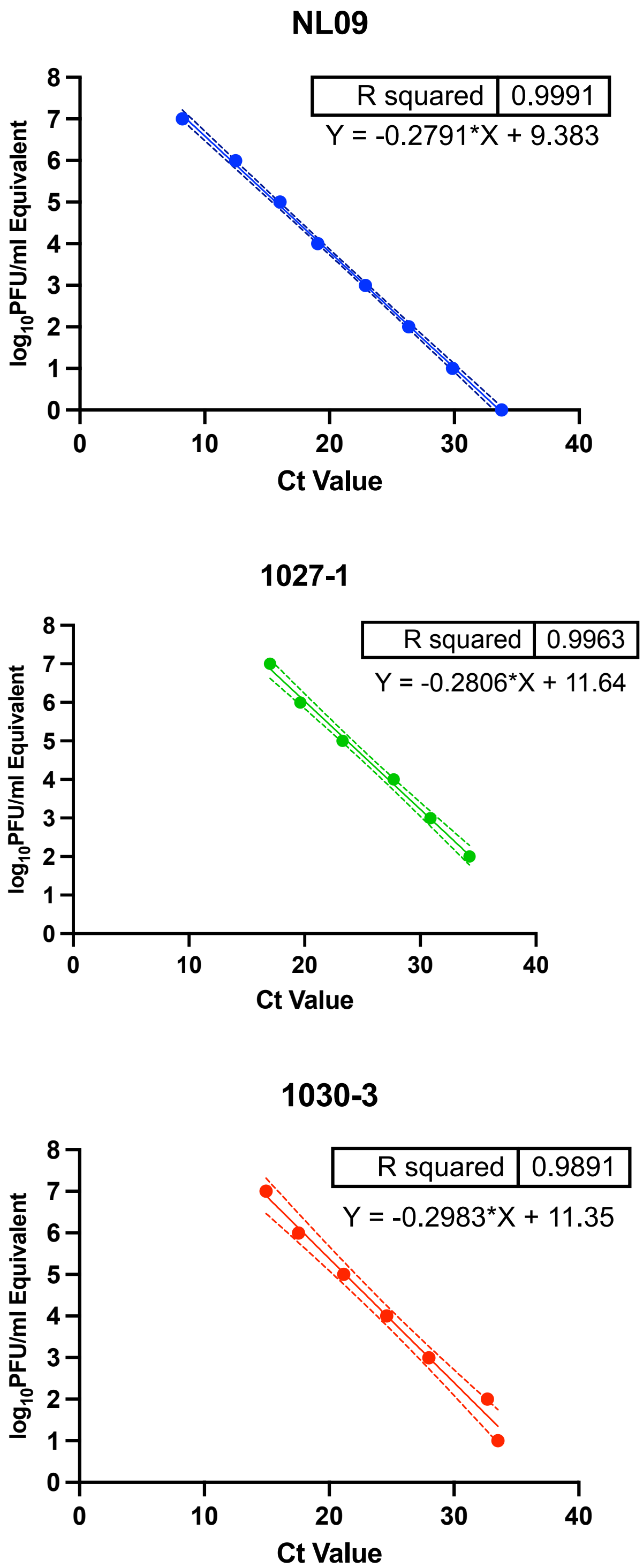

Supplementary Figure S5. Standard curves for conversion of RT-qPCR Ct values to PFU-equivalent viral loads. Standard curves generated from serial dilutions of virus stocks with known infectious titers for (A) NL09, (B) 1027-1 and (C) 1030-3. Linear regression equations and R^2 values are shown in each panel.
