# Supplementary material for: Pathogenicity and zoonotic potential of a novel pdm09-lineage H1N2 swine influenza A virus spatiotemporally linked to transboundary pig trade
Source: PLoS Pathog. 2026 Nov 3;22(11):e1014779. doi: 10.1371/journal.ppat.1014779 (PMC13743032; doi:10.1371/journal.ppat.1014779)
Supplement: Supplementary file 6 [file ppat.1014779.s006.pdf]

**Supplementary Table S1.** Country-specific pig importation data from major exporting nations during 1996-2024.

All data are available in the Korean Animal and Plant Quarantine Agency (https://www.qia.go.kr/). Values represent the number of pigs imported.

| Year | Canada | USA | France | Denmark | UK | Sweden | Japan | Australia | Ireland | Finland | Vietnam |
|---|---|---|---|---|---|---|---|---|---|---|---|
| **1996** | 317 | 1241 | 0 | 136 | 687 | 0 | 52 | 22 | 25 | 0 | 0 |
| **1997** | 1950 | 1344 | 0 | 0 | 486 | 108 | 0 | 34 | 0 | 0 | 0 |
| **1998** | 35 | 218 | 0 | 0 | 153 | 0 | 0 | 0 | 0 | 0 | 4 |
| **1999** | 487 | 888 | 0 | 138 | 179 | 0 | 0 | 0 | 0 | 0 | 0 |
| **2000** | 538 | 579 | 0 | 164 | 486 | 0 | 0 | 0 | 35 | 58 | 0 |
| **2001** | 355 | 1059 | 0 | 0 | 0 | 0 | 0 | 30 | 0 | 0 | 0 |
| **2002** | 331 | 890 | 0 | 19 | 0 | 0 | 0 | 0 | 0 | 0 | 0 |
| **2003** | 305 | 646 | 0 | 0 | 0 | 0 | 23 | 0 | 0 | 0 | 0 |
| **2004** | 404 | 800 | 0 | 54 | 196 | 37 | 0 | 0 | 0 | 0 | 0 |
| **2005** | 355 | 1309 | 0 | 50 | 62 | 0 | 0 | 0 | 0 | 0 | 0 |
| **2006** | 912 | 1210 | 0 | 89 | 43 | 32 | 0 | 0 | 0 | 0 | 0 |
| **2007** | 1507 | 700 | 254 | 41 | 97 | 0 | 26 | 0 | 0 | 0 | 0 |
| **2008** | 977 | 592 | 150 | 46 | 0 | 0 | 0 | 0 | 0 | 0 | 0 |
| **2009** | 102 | 214 | 0 | 0 | 0 | 0 | 5 | 0 | 0 | 0 | 0 |
| **2010** | 1337 | 480 | 424 | 46 | 0 | 0 | 19 | 0 | 0 | 0 | 0 |
| **2011** | 11299 | 4430 | 0 | 0 | 0 | 0 | 0 | 0 | 0 | 0 | 0 |
| **2012** | 6516 | 440 | 3995 | 92 | 0 | 0 | 0 | 0 | 0 | 0 | 0 |
| **2013** | 357 | 966 | 966 | 0 | 0 | 0 | 0 | 0 | 0 | 0 | 0 |
| **2014** | 952 | 276 | 511 | 32 | 0 | 0 | 0 | 0 | 0 | 0 | 0 |
| **2015** | 1100 | 451 | 219 | 161 | 0 | 0 | 0 | 0 | 0 | 0 | 0 |
| **2016** | 1887 | 489 | 515 | 288 | 0 | 0 | 0 | 0 | 0 | 0 | 0 |
| **2017** | 2750 | 230 | 1261 | 664 | 0 | 0 | 0 | 0 | 0 | 0 | 0 |
| **2018** | 1033 | 141 | 346 | 730 | 0 | 0 | 0 | 0 | 0 | 0 | 0 |
| **2019** | 558 | 169 | 23 | 289 | 0 | 0 | 0 | 0 | 0 | 0 | 0 |
| **2020** | 571 | 31 | 0 | 336 | 0 | 0 | 0 | 0 | 0 | 0 | 0 |
| **2021** | 809 | 237 | 54 | 520 | 0 | 0 | 0 | 0 | 0 | 0 | 0 |
| **2022** | 1286 | 41 | 48 | 332 | 0 | 0 | 0 | 0 | 0 | 0 | 0 |
| **2023** | 2492 | 143 | 370 | 907 | 0 | 0 | 0 | 0 | 0 | 0 | 0 |
| **2024** | 930 | 257 | 24 | 681 | 0 | 0 | 0 | 0 | 0 | 0 | 0 |
| **Total** | 42452 | 20471 | 9160 | 5815 | 2389 | 177 | 125 | 86 | 60 | 58 | 4 |
