# Supplementary material for: Pathogenicity and zoonotic potential of a novel pdm09-lineage H1N2 swine influenza A virus spatiotemporally linked to transboundary pig trade
Source: PLoS Pathog. 2026 Nov 3;22(11):e1014779. doi: 10.1371/journal.ppat.1014779 (PMC13743032; doi:10.1371/journal.ppat.1014779)
Supplement: Supplementary file 7 [file ppat.1014779.s007.pdf]

Supplementary Table S2. Clinical and vaccination meta data of human serum samples.

serum ID	Birth year	Vaccination status	Vaccination date	Prior IAV infection	Reported influenza infection date
1	1988	No	-	Yes	2024-01-01
2	1976	No	-	Yes	2024-12-28
3	1996	No	-	Yes	2025-01-09
4	1983	No	-	Yes	2024-04-09
5	1973	Yes	2024-10-14	Yes	2024-01-02
6	1991	Yes	2024-10-25	Yes	2025-01-06
7	1985	Yes	2024-10-18	Yes	2025-01-02
8	1993	Yes	2024-10-21	Yes	2025-01-09
9	1998	Yes	2024-10-22	No	-
10	1997	Yes	2024-10-17	No	-
11	1991	Yes	2024-10-21	No	-
12	1997	Yes	2024-10-22	No	-
13	1999	No	-	No	-
