# Supplementary material for: Pathogenicity and zoonotic potential of a novel pdm09-lineage H1N2 swine influenza A virus spatiotemporally linked to transboundary pig trade
Source: PLoS Pathog. 2026 Nov 3;22(11):e1014779. doi: 10.1371/journal.ppat.1014779 (PMC13743032; doi:10.1371/journal.ppat.1014779)
Supplement: Supplementary file 8 [file ppat.1014779.s008.pdf]

Supplementary Table S3. Summary of HA sequence datasets before and after CD-HIT clustering by host and geographic origin

	Country	Before CD-HIT	After CD-HIT	CD-HIT Threshold
**Swine IAV**	South Korea	97	55	99.9
	Japan	485	75	98.5
	China	759	122	98.5
	South East Asia	462	31	98.5
	Europe	2187	359	97
	North America	3042	185	97
	**SUM**	**7032**	**827**	
**Human -pdm09 IAV**	**Country**	**Before CD-HIT**	**After CD-HIT**	**CD-HIT Threshold**
	Asia	1988	29	98.5
	Europe	16584	30	98.5
	North America	19064	33	98.5
	**SUM**	**37636**	**92**	
**Reference IAV**	Vaccine	4	-	-
	Wild bird	5	-	-
	Swine	12	-	-
