# Supplementary material for: Pathogenicity and zoonotic potential of a novel pdm09-lineage H1N2 swine influenza A virus spatiotemporally linked to transboundary pig trade
Source: PLoS Pathog. 2026 Nov 3;22(11):e1014779. doi: 10.1371/journal.ppat.1014779 (PMC13743032; doi:10.1371/journal.ppat.1014779)
Supplement: Supplementary file 9 [file ppat.1014779.s009.pdf]

Supplementary Table S4. Complete list of H1 influenza A virus isolates with GISAID accessions, HA clade assignments, and metadata.

WHO-recommended pdm09 vaccine strains are highlighted in red.

IAV strain	HA (H1) clade	GISAID ID	Subtype	Region	Host
A/green_winged_teal/Ohio/72/1999	North American Avian		H1N1	North America	wild bird
A/blue_winged_teal/TX/27/2002	North American Avian		H1N1	North America	wild bird
A/pintail/Alberta/68/2005	North American Avian		H1N1	North America	wild bird
A/duck/Mongolia/520/2015	Eurasian Avian	EPI_ISL_91542	H1N1	China	wild bird
A/duck/Shimane/188/1999	Eurasian Avian	EPI_ISL_206573	H1N1	Japan	wild bird
A/swine/Marseille/2260/1980	EAS (1C.1)	EPI_ISL_170518	H1N1	Europe	swine
A/swine/Bakum/3543/1998	EAS (1C.1)	EPI_ISL_504556	H1N1	Europe	swine
A/swine/Italy/670/1987	EAS (1C.1)		H1N1	Europe	swine
A/swine/Germany/Vi5698/95	EAS (1C.1)		H1N1	Europe	swine
A/swine/Austria/516-05/1997	EAS (1C.1)	EPI_ISL_18592209	H1N1	Europe	swine
A/swine/France/WVL13/1995	EAS (1C.1)	EPI_ISL_29412	H1N1	Europe	swine
A/swine/OMS/2112/1995	EAS (1C.1)	EPI_ISL_129518	H1N1	North America	swine
A/swine/Gent/V230/1992	EAS (1C.1)	EPI_ISL_29097	H1N1	Europe	swine
A/swine/England/745769/1995	EAS (1C.1)	EPI_ISL_129501	H1N1	Europe	swine
A/swine/England/254/2002	EAS (1C.1)	EPI_ISL_129472	H1N1	Europe	swine
A/swine/England/383/2005	EAS (1C.1)	EPI_ISL_129482	H1N1	Europe	swine
A/swine/England/001195/2007	EAS (1C.1)	EPI_ISL_129451	H1N1	Europe	swine
A/swine/England/167655/1997	EAS (1C.1)	EPI_ISL_129464	H1N1	Europe	swine
A/swine/England/35320/1999	EAS (1C.1)	EPI_ISL_129479	H1N1	Europe	swine
A/swine/England/193667/1998	EAS (1C.1)	EPI_ISL_129467	H1N1	Europe	swine
A/swine/England/453/2006	EAS (1C.1)	EPI_ISL_129490	H1N1	Europe	swine
A/swine/England/004772/2001	EAS (1C.1)	EPI_ISL_129453	H1N1	Europe	swine
A/swine/Scotland/034632/2012	EAS (1C.1)	EPI_ISL_195130	H1N1	Europe	swine
A/swine/England/024079/2013	EAS (1C.1)	EPI_ISL_195168	H1N1	Europe	swine
A/swine/England/1251/2011	EAS (1C.1)	EPI_ISL_195124	H1N1	Europe	swine
A/swine/England/010402/2003	EAS (1C.1)	EPI_ISL_129454	H1N1	Europe	swine
A/swine/Gent/74/95	EAS (1C.2)	EPI_ISL_199473	H1N1	Europe	swine
A/swine/Italy/1513-1/1998	EAS (1C.2)	EPI_ISL_129521	H1N1	Europe	swine
A/swine/Italy/1369-7/1994	EAS (1C.2)	EPI_ISL_95804	H1N1	Europe	swine
A/swine/Denmark/WVL9/1993	EAS (1C.2)		H1N1	Europe	swine
A/swine/Denmark/19126/1993	EAS (1C.2)		H1N1	Europe	swine
A/swine/Belgium/1/98	EAS (1C.2)	EPI_ISL_199470	H1N1	Europe	swine
A/swine/Denmark/10169-3/2012	EAS (1C.2)	EPI_ISL_195053	H1N1	Europe	swine
A/swine/Italy/187946/2015	EAS (1C.2)	EPI_ISL_278832	H1N2	Europe	swine
A/swine/Denmark/10779-2/2012	EAS (1C.2)	EPI_ISL_195050	H1N2	Europe	swine
A/swine/Denmark/10297-1/2012	EAS (1C.2)	EPI_ISL_195047	H1N2	Europe	swine
A/swine/Emmelsbuell/9876/2009	EAS (1C.2)	EPI_ISL_505178	H1N2	Europe	swine
A/swine/Italy/19-162065-4/2019	EAS (1C.2)	EPI_ISL_4083723	H1N2	Europe	swine
A/swine/Denmark/2018_2511_1_1p1/2018	EAS (1C.2)	EPI_ISL_4081353	H1N2	Europe	swine
A/swine/Guetersloh/15035/2012	EAS (1C.2)	EPI_ISL_505243	H1N2	Europe	swine
A/swine/Italy/75997/2020	EAS (1C.2)	EPI_ISL_14764694	H1N1	Europe	swine
A/swine/Italy/282881/2013	EAS (1C.2)	EPI_ISL_278911	H1N1	Europe	swine
A/swine/Italy/91681-27/2022	EAS (1C.2)	EPI_ISL_14937239	H1N1	Europe	swine
A/swine/Denmark/2014_16252_1p1/2014	EAS (1C.2)	EPI_ISL_4081348	H1N2	Europe	swine
A/swine/Italy/19-142834-30/2019	EAS (1C.2)	EPI_ISL_4083720	H1N2	Europe	swine
A/swine/Denmark/2018_3368_2_1p1/2018	EAS (1C.2)	EPI_ISL_4081347	H1N1	Europe	swine
A/swine/Italy/58769/2010	EAS (1C.2)	EPI_ISL_79837	H1N2	Europe	swine
A/swine/Denmark/S22282-5/2021	EAS (1C.2)	EPI_ISL_9605609	H1N2	Europe	swine
A/swine/Wrestedt/19450/2014	EAS (1C.2)	EPI_ISL_505303	H1N2	Europe	swine
A/swine/Sweden/1021/2009	EAS (1C.2)	EPI_ISL_61669	H1N2	Europe	swine
A/swine/Germany/AR3006/2016	EAS (1C.2)	EPI_ISL_238062	H1N2	Europe	swine
A/swine/Ennigerloh/14785-2/2012	EAS (1C.2)	EPI_ISL_505238	H1N2	Europe	swine
A/swine/Haseluenne/IDT2617/03	EAS (1C.2.2)	EPI_ISL_13578	H1N1	Europe	swine
A/swine/Spain/31174/2019	EAS (1C.2.2)	EPI_ISL_9593374	H1N1	Europe	swine
A/swine/Spain/22253-1/2020	EAS (1C.2.2)	EPI_ISL_18921587	H1N1	Europe	swine
A/swine/Spain/50840/2019	EAS (1C.2.2)	EPI_ISL_9593382	H1N1	Europe	swine
A/swine/Spain/29113/2012	EAS (1C.2.2)	EPI_ISL_195059	H1N1	Europe	swine
A/Swine/Spain/50047/2003	EAS (1C.2.2)	EPI_ISL_5203	H1N1	Europe	swine
A/swine/Brakel/4806/2005	EAS (1C.2.2)	EPI_ISL_504584	H1N1	Europe	swine
A/swine/Poland/006801/2013	EAS (1C.2.2)	EPI_ISL_195172	H1N1	Europe	swine

A/swine/Poland/1744/2012	EAS (1C.2.2)	EPI_ISL_195162	H1N1	Europe	swine
A/swine/Bothenheilingen/13765-3/2011	EAS (1C.2.2)	EPI_ISL_504750	H1N1	Europe	swine
A/swine/Engelsberg/9321/2009	EAS (1C.2.2)	EPI_ISL_504674	H1N1	Europe	swine
A/swine/Italy/34135/2019	EAS (1C.2.2)	EPI_ISL_14749683	H1N1	Europe	swine
A/swine/Poland/321/2012	EAS (1C.2.2)	EPI_ISL_195163	H1N1	Europe	swine
A/Poland/wild_boar/1951713/2013	EAS (1C.2.2)	EPI_ISL_209353	H1N1	Europe	swine
A/swine/Spain/05530-1/2020	EAS (1C.2.2)	EPI_ISL_18921490	H1N2	Europe	swine
A/swine/Spain/50267/2019	EAS (1C.2.2)	EPI_ISL_9593378	H1N2	Europe	swine
A/swine/Spain/6370/2018	EAS (1C.2.2)	EPI_ISL_9593375	H1N2	Europe	swine
A/swine/Fronhausen/6059/2007	EAS (1C.2.2)	EPI_ISL_504616	H1N1	Europe	swine
A/swine/France/01-130145/2013	EAS (1C.2.2)	EPI_ISL_502448	H1N1	Europe	swine
A/swine/Mengkofen/17020/2013	EAS (1C.2.2)	EPI_ISL_504813	H1N1	Europe	swine
A/swine/Erwitte/19661/2014	EAS (1C.2.2)	EPI_ISL_504830	H1N1	Europe	swine
A/swine/Steinfeld/13785/2011	EAS (1C.2.2)	EPI_ISL_504751	H1N1	Europe	swine
A/swine/Germany/2022AI01351/2022	EAS (1C.2.2)	EPI_ISL_17646264	H1N1	Europe	swine
A/swine/Netherlands/Dalfsen-12/2012	EAS (1C.2.2)		H1N1	Europe	swine
A/swine/Germany/2022AI00354/2022	EAS (1C.2.2)	EPI_ISL_17646276	H1N1	Europe	swine
A/Swine/Netherlands/GDE0023/2023	EAS (1C.2.2)	EPI_ISL_18801986	H1N1	Europe	swine
A/Swine/Netherlands/GDE0052/2023	EAS (1C.2.2)	EPI_ISL_18802008	H1N1	Europe	swine
A/Swine/Netherlands/GDE0037/2023	EAS (1C.2.2)	EPI_ISL_18801996	H1N1	Europe	swine
A/swine/Germany/2022AI01090/2022	EAS (1C.2.2)	EPI_ISL_17646268	H1N1	Europe	swine
A/Swine/Netherlands/GDE0058/2023	EAS (1C.2.2)	EPI_ISL_18802010	H1N2	Europe	swine
A/swine/England/048994/2023	EAS (1C.2.2)	EPI_ISL_18586952	H1N1	Europe	swine
A/swine/Germany/2022AI03311/2022	EAS (1C.2.2)	EPI_ISL_17646250	H1N1	Europe	swine
A/swine/Germany/2023AI01627/2023	EAS (1C.2.2)	EPI_ISL_17646298	H1N1	Europe	swine
A/swine/Germany/2022AI02969/2022	EAS (1C.2.2)	EPI_ISL_17646253	H1N1	Europe	swine
A/Swine/Netherlands/GDE0020/2023	EAS (1C.2.2)	EPI_ISL_18802198	H1N1	Europe	swine
A/Swine/Netherlands/GDE0106/2022	EAS (1C.2.2)	EPI_ISL_18802195	H1N1	Europe	swine
A/swine/Germany/2022AI04737/2022	EAS (1C.2.2)	EPI_ISL_17646239	H1N1	Europe	swine
A/swine/Germany/2022AI05247/2022	EAS (1C.2.2)	EPI_ISL_17646237	H1N1	Europe	swine
A/Swine/Netherlands/GDE0083/2023	EAS (1C.2.2)	EPI_ISL_18802141	H1N2	Europe	swine
A/swine/Cotes_dArmor/1624/2002	EAS (1C.2.3)	EPI_ISL_129516	H1N1	Europe	swine
A/swine/Finnistere/800/2004	EAS (1C.2.3)	EPI_ISL_129511	H1N1	Europe	swine
A/swine/Guangxi/S2/2013	EAS (1C.2.3)	EPI_ISL_157896	H1N1	China	swine
A/swine/Guangdong/1604/2010	EAS (1C.2.3)	EPI_ISL_96966	H1N1	China	swine
A/Swine/Guangdong/52/2017	EAS (1C.2.3)	EPI_ISL_293280	H1N1	China	swine
A/swine/Guangdong/104/2013	EAS (1C.2.3)	EPI_ISL_176792	H1N1	China	swine
A/swine/Chongqing/241/2017	EAS (1C.2.3)	EPI_ISL_12940969	H1N1	China	swine
A/swine/Guangdong/SS1/2012	EAS (1C.2.3)	EPI_ISL_166533	H1N1	China	swine
A/swine/Henan/11/2005	EAS (1C.2.3)	EPI_ISL_132838	H1N1	China	swine
A/swine/Liaoning/32/2006	EAS (1C.2.3)	EPI_ISL_76963	H1N1	China	swine
A/swine/Guangdong/1361/2010	EAS (1C.2.3)	EPI_ISL_96950	H1N1	China	swine
A/swine/China/Qingdao/2018	EAS (1C.2.3)	EPI_ISL_370660	H1N1	China	swine
A/swine/Beijing/21/2008	EAS (1C.2.3)	EPI_ISL_33881	H1N1	China	swine
A/swine/Zhejiang/43/2012	EAS (1C.2.3)	EPI_ISL_171206	H1N2	China	swine
A/swine/Guangdong/2/2013	EAS (1C.2.3)	EPI_ISL_169556	H1N1	China	swine
A/swine/Guangdong/32/2013	EAS (1C.2.3)	EPI_ISL_169558	H1N1	China	swine
A/swine/Hunan/951/2013	EAS (1C.2.3)	EPI_ISL_169576	H1N1	China	swine
A/swine/Henan/232/2011	EAS (1C.2.3)	EPI_ISL_169569	H1N1	China	swine
A/swine/Jiangsu/zg4/2010	EAS (1C.2.3)	EPI_ISL_109819	H1N1	China	swine
A/swine/Korea/CY0423-33/2013	EAS (1C.2.3)	EPI_ISL_142741	H1N2	South Korea	swine
A/swine/South_Korea/CVM-0070/2022	EAS (1C.2.3)	EPI_ISL_18708438	H1N2	South Korea	swine
A/swine/South_Korea/CVM-0608/2021	EAS (1C.2.3)	EPI_ISL_18708363	H1N2	South Korea	swine
A/swine/South_Korea/CVM-0388/2019	EAS (1C.2.3)	EPI_ISL_18708362	H1N2	South Korea	swine
A/swine/South_Korea/CVM-0677/2021	EAS (1C.2.3)	EPI_ISL_18708385	H1N2	South Korea	swine
A/swine/South_Korea/s803/2018	EAS (1C.2.3)	EPI_ISL_17885888	H1N2	South Korea	swine
A/swine/South_Korea/s802/2018	EAS (1C.2.3)	EPI_ISL_14174097	H1N2	South Korea	swine
A/swine/South_Korea/CVM-06323/2021	EAS (1C.2.3)	EPI_ISL_18708366	H1N2	South Korea	swine
A/swine/South_Korea/CVM-0841/2021	EAS (1C.2.3)	EPI_ISL_18708392	H1N2	South Korea	swine
A/swine/South_Korea/CVM-0074/2022	EAS (1C.2.3)	EPI_ISL_18708393	H1N2	South Korea	swine
A/swine/South_Korea/CVM-0727/2021	EAS (1C.2.3)	EPI_ISL_18708388	H1N2	South Korea	swine
A/swine/South_Korea/CVM-0046/2022	EAS (1C.2.3)	EPI_ISL_18708409	H1N2	South Korea	swine
A/swine/South_Korea/KBUS/2021	EAS (1C.2.3)	EPI_ISL_16216025	H1N2	South Korea	swine
A/swine/Heilongjiang/27/2012	EAS (1C.2.3)	EPI_ISL_169566	H1N1	China	swine
A/swine/Jiangsu/s15/2011	EAS (1C.2.3)	EPI_ISL_148288	H1N1	China	swine
A/swine/Taian/23/2017	EAS (1C.2.3)	EPI_ISL_389785	H1N1	China	swine
A/swine/Hubei/2/2014	EAS (1C.2.3)	EPI_ISL_12958025	H1N1	China	swine
A/swine/Guangxi/GG6/2013	EAS (1C.2.3)	EPI_ISL_282075	H1N1	China	swine
A/swine/Guangxi/BB2/2013	EAS (1C.2.3)	EPI_ISL_282074	H1N1	China	swine
A/swine/Tianjin/42/2011	EAS (1C.2.3)	EPI_ISL_169585	H1N1	China	swine

A/swine/Hunan/26/2010	EAS (1C.2.3)	EPI_ISL_132836	H1N1	China	swine
A/swine/Hunan/269/2016	EAS (1C.2.3)	EPI_ISL_12958029	H1N1	China	swine
A/swine/Jiangxi/400/2015	EAS (1C.2.3)	EPI_ISL_12958179	H1N1	China	swine
A/swine/Jiangsu/zg13/2011	EAS (1C.2.3)	EPI_ISL_109826	H1N1	China	swine
A/Jiangsu/1/2011	EAS (1C.2.3)	EPI_ISL_505579	H1N1	China	human
A/Jiangsu/ALS1/2011	EAS (1C.2.3)	EPI_ISL_170785	H1N1	China	human
A/swine/Shanghai/3/2014	EAS (1C.2.3)	EPI_ISL_167155	H1N1	China	swine
A/swine/Jiangsu/89/2011	EAS (1C.2.3)	EPI_ISL_482256	H1N1	China	swine
A/Swine/Guangdong/MS493/2017	EAS (1C.2.3)	EPI_ISL_293283	H1N1	China	swine
A/swine/Jiangxi/186/2017	EAS (1C.2.3)	EPI_ISL_12958465	H1N1	China	swine
A/swine/Guangxi/749/2017	EAS (1C.2.3)	EPI_ISL_12957485	H1N1	China	swine
A/swine/Jilin/280/2017	EAS (1C.2.3)	EPI_ISL_12958186	H1N1	China	swine
A/swine/Guangdong/473/2014	EAS (1C.2.3)	EPI_ISL_12941097	H1N1	China	swine
A/swine/Guangxi/10/2016	EAS (1C.2.3)	EPI_ISL_12941148	H1N1	China	swine
A/swine/Tianjin/282/2018	EAS (1C.2.3)	EPI_ISL_12958417	H1N1	China	swine
A/swine/Guizhou/828/2016	EAS (1C.2.3)	EPI_ISL_10398540	H1N1	China	swine
A/swine/Tianjin/301/2018	EAS (1C.2.3)	EPI_ISL_12958419	H1N1	China	swine
A/Swine/Guangdong/BRT15/2015	EAS (1C.2.3)	EPI_ISL_249812	H1N1	China	swine
A/Swine/Guangdong/MS285/2017	EAS (1C.2.3)	EPI_ISL_293281	H1N1	China	swine
A/swine/Chongqing/705/2016	EAS (1C.2.3)	EPI_ISL_12941032	H1N1	China	swine
A/swine/Shandong/JM78/2017	EAS (1C.2.3)	EPI_ISL_482283	H1N1	China	swine
A/swine/Anhui/1227/2015	EAS (1C.2.3)	EPI_ISL_482221	H1N1	China	swine
A/swine/Chongqing/104/2016	EAS (1C.2.3)	EPI_ISL_12940946	H1N1	China	swine
A/swine/Guangxi/576/2018	EAS (1C.2.3)	EPI_ISL_12957478	H1N1	China	swine
A/swine/Anhui/53/2018	EAS (1C.2.3)	EPI_ISL_12939617	H1N1	China	swine
A/Swine/Guangdong/FSC17/2015	EAS (1C.2.3)	EPI_ISL_249852	H1N1	China	swine
A/swine/Shaanxi/1150/2016	EAS (1C.2.3)	EPI_ISL_12958226	H1N1	China	swine
A/swine/Sichuan/1142/2017	EAS (1C.2.3)	EPI_ISL_12958394	H1N1	China	swine
A/swine/Yunnan/221/2018	EAS (1C.2.3)	EPI_ISL_12958466	H1N1	China	swine
A/swine/Chongqing/622/2016	EAS (1C.2.3)	EPI_ISL_12941025	H1N1	China	swine
A/swine/Sichuan/492/2017	EAS (1C.2.3)	EPI_ISL_12958399	H1N1	China	swine
A/swine/Jiangxi/699/2017	EAS (1C.2.3)	EPI_ISL_12958183	H1N1	China	swine
A/swine/Chongqing/563/2016	EAS (1C.2.3)	EPI_ISL_12941017	H1N1	China	swine
A/swine/Jiangsu/462/2017	EAS (1C.2.3)	EPI_ISL_12958040	H1N1	China	swine
A/swine/Guangdong/132/2019	EAS (1C.2.3)	EPI_ISL_12941074	H1N1	China	swine
A/swine/Hebei/292/2018	EAS (1C.2.3)	EPI_ISL_12957532	H1N1	China	swine
A/swine/Liaoning/AS1732/2020	EAS (1C.2.3)	EPI_ISL_14768127	H1N1	China	swine
A/swine/Liaoning/TL5239/2020	EAS (1C.2.3)	EPI_ISL_14768135	H1N1	China	swine
A/swine/Henan/764/2019	EAS (1C.2.3)	EPI_ISL_12957977	H1N1	China	swine
A/swine/Jiangsu/45/2019	EAS (1C.2.3)	EPI_ISL_12958039	H1N1	China	swine
A/swine/Liaoning/TL5404/2020	EAS (1C.2.3)	EPI_ISL_14768136	H1N1	China	swine
A/swine/Jilin/683/2019	EAS (1C.2.3)	EPI_ISL_12958198	H1N1	China	swine
A/swine/Henan/208/2019	EAS (1C.2.3)	EPI_ISL_12957779	H1N1	China	swine
A/swine/Liaoning/265/2017	EAS (1C.2.3)	EPI_ISL_10398539	H1N1	China	swine
A/swine/Anhui/335/2018	EAS (1C.2.3)	EPI_ISL_12939610	H1N1	China	swine
A/swine/Jiangsu/629/2019	EAS (1C.2.3)	EPI_ISL_12958045	H1N1	China	swine
A/swine/Shaanxi/428/2017	EAS (1C.2.3)	EPI_ISL_12958235	H1N1	China	swine
A/swine/Anhui/589/2015	EAS (1C.2.3)	EPI_ISL_12940930	H1N1	China	swine
A/swine/Liaoning/1011/2016	EAS (1C.2.3)	EPI_ISL_12958203	H1N1	China	swine
A/swine/Guangxi/324/2016	EAS (1C.2.3)	EPI_ISL_12957210	H1N1	China	swine
A/swine/Guangxi/276/2018	EAS (1C.2.3)	EPI_ISL_12957206	H1N1	China	swine
A/swine/Guangxi/264/2019	EAS (1C.2.3)	EPI_ISL_12941321	H1N1	China	swine
A/Hunan/42443/2015	EAS (1C.2.3)	EPI_ISL_505578	H1N1	China	human
A/swine/Chongqing/112/2018	EAS (1C.2.3)	EPI_ISL_12940953	H1N1	China	swine
A/swine/Hubei/221/2016	EAS (1C.2.3)	EPI_ISL_282052	H1N1	China	swine
A/swine/Henan/606/2018	EAS (1C.2.3)	EPI_ISL_12957974	H1N1	China	swine
A/swine/Guangxi/416/2016	EAS (1C.2.3)	EPI_ISL_12957474	H1N1	China	swine
A/swine/Guangxi/206/2016	EAS (1C.2.3)	EPI_ISL_12941297	H1N1	China	swine
A/swine/Liaoning/JZ164/2020	EAS (1C.2.3)	EPI_ISL_14768117	H1N1	China	swine
A/swine/Hubei/580/2019	EAS (1C.2.3)	EPI_ISL_12958027	H1N1	China	swine
A/swine/Liaoning/10/2015	EAS (1C.2.3)	EPI_ISL_12958202	H1N1	China	swine
A/swine/Hebei/101/2016	EAS (1C.2.3)	EPI_ISL_12957500	H1N1	China	swine
A/swine/Heilongjiang/1085/2015	EAS (1C.2.3)	EPI_ISL_12957770	H1N1	China	swine
A/swine/Taian/95/2017	EAS (1C.2.3)	EPI_ISL_389786	H1N1	China	swine
A/swine/Shandong/60/2019	EAS (1C.2.3)	EPI_ISL_12958385	H1N1	China	swine
A/swine/Guizhou/132/2016	EAS (1C.2.3)	EPI_ISL_12957495	H1N1	China	swine
A/swine/Shandong/123/2018	EAS (1C.2.3)	EPI_ISL_12958240	H1N1	China	swine
A/swine/Guangxi/118/2017	EAS (1C.2.3)	EPI_ISL_12941217	H1N1	China	swine
A/swine/Anhui/583/2017	EAS (1C.2.3)	EPI_ISL_12940921	H1N1	China	swine
A/swine/Liaoning/248/2018	EAS (1C.2.3)	EPI_ISL_12958210	H1N1	China	swine

A/swine/Zhejiang/132/2019	EAS (1C.2.3)	EPI_ISL_12958451	H1N1	China	swine
A/swine/Jilin/164/2019	EAS (1C.2.3)	EPI_ISL_12958184	H1N1	China	swine
A/swine/Liaoning/FS1084/2020	EAS (1C.2.3)	EPI_ISL_14768123	H1N1	China	swine
A/swine/Liaoning/HLD1795/2020	EAS (1C.2.3)	EPI_ISL_14768130	H1N1	China	swine
A/swine/Liaoning/PJ521/2020	EAS (1C.2.3)	EPI_ISL_14768119	H1N1	China	swine
A/swine/Liaoning/SY369/2020	EAS (1C.2.3)	EPI_ISL_14768118	H1N1	China	swine
A/swine/Liaoning/DL5436/2020	EAS (1C.2.3)	EPI_ISL_14768137	H1N1	China	swine
A/swine/Liaoning/LY3468/2020	EAS (1C.2.3)	EPI_ISL_14768133	H1N1	China	swine
A/swine/Italy/22530/2002	EAS (1C.2.1)	EPI_ISL_95884	H1N2	Europe	swine
A/swine/Bieganow/1/2001	EAS (1C.2.1)	EPI_ISL_129447	H1N1	Europe	swine
A/swine/Denmark/2015_04775_1p1/2015	EAS (1C.2.1)	EPI_ISL_4081263	H1N2	Europe	swine
A/swine/Denmark/10802-1/2012	EAS (1C.2.1)	EPI_ISL_195051	H1N2	Europe	swine
A/swine/Homberg/7039/2008	EAS (1C.2.1)	EPI_ISL_504640	H1N1	Europe	swine
A/swine/Denmark/2017_2139_4_1p1/2017	EAS (1C.2.1)	EPI_ISL_4081264	H1N1	Europe	swine
A/swine/Boesel/22002/2015	EAS (1C.2.1)	EPI_ISL_505347	H1N2	Europe	swine
A/swine/Ellerbrock/18558/2013	EAS (1C.2.1)	EPI_ISL_505285	H1N2	Europe	swine
A/swine/Garrel/20222/2014	EAS (1C.2.1)	EPI_ISL_505317	H1N2	Europe	swine
A/swine/Italy/17-147155-16/2017	EAS (1C.2.1)	EPI_ISL_4083714	H1N2	Europe	swine
A/swine/Denmark/2014_10_786_3p1/2014	EAS (1C.2.1)	EPI_ISL_4081267	H1N2	Europe	swine
A/swine/Denmark/2013_10_1326_1p1/2013	EAS (1C.2.1)	EPI_ISL_4081269	H1N1	Europe	swine
A/swine/Denmark/10448-3/2012	EAS (1C.2.1)	EPI_ISL_195049	H1N2	Europe	swine
A/swine/Garrel/20866/2014	EAS (1C.2.1)	EPI_ISL_505325	H1N2	Europe	swine
A/swine/Italy/101797/2012	EAS (1C.2.1)	EPI_ISL_278924	H1N1	Europe	swine
A/swine/Germany/R404/2014	EAS (1C.2.1)	EPI_ISL_245681	H1N2	Europe	swine
A/swine/Denmark/2014_7191_2p1/2014	EAS (1C.2.1)	EPI_ISL_4081394	H1N2	Europe	swine
A/swine/Grossharie/4372/2005	EAS (1C.2.1)	EPI_ISL_504572	H1N1	Europe	swine
A/swine/Wasserleben/13484/2011	EAS (1C.2.1)	EPI_ISL_504742	H1N1	Europe	swine
A/swine/Denmark/2013_30_5127_12p1/2013	EAS (1C.2.1)	EPI_ISL_4081396	H1N2	Europe	swine
A/swine/Borken/10752a/2009	EAS (1C.2.1)	EPI_ISL_504692	H1N1	Europe	swine
A/swine/Denmark/2013_10_13_4p1/2013	EAS (1C.2.1)	EPI_ISL_4081398	H1N1	Europe	swine
A/swine/Denmark/2016_4025_1p1/2016	EAS (1C.2.1)	EPI_ISL_4081397	H1N1	Europe	swine
A/swine/Emstek/12594a/2010	EAS (1C.2.1)	EPI_ISL_505195	H1N2	Europe	swine
A/swine/Denmark/2013_10_84_2p1/2013	EAS (1C.2.1)	EPI_ISL_4081359	H1N2	Europe	swine
A/swine/Denmark/10345-1/2012	EAS (1C.2.1)	EPI_ISL_195100	H1N2	Europe	swine
A/swine/Denmark/2018_4528_2_1p1/2018	EAS (1C.2.1)	EPI_ISL_4081360	H1N2	Europe	swine
A/swine/Italy/102227/2019	EAS (1C.2.1)	EPI_ISL_14749719	H1N2	Europe	swine
A/swine/Fjerritslev/19884-P5/2014	EAS (1C.2.1)	EPI_ISL_505312	H1N2	Europe	swine
A/swine/Denmark/2018_13990_1_1p1/2018	EAS (1C.2.1)	EPI_ISL_4081361	H1N2	Europe	swine
A/swine/Denmark/2016_15673_1p1/2016	EAS (1C.2.1)	EPI_ISL_4081369	H1N2	Europe	swine
A/swine/Ba_Ria_Vung_Tau/02-07-2/2016	EAS (1C.2.1)	EPI_ISL_257494	H1N2	South East Asia	swine
A/sw/Sachsen-Anhalt/3/2021	EAS (1C.2.1)	EPI_ISL_12589311	H1N2	Europe	swine
A/swine/Germany/Bak50/2017	EAS (1C.2.1)	EPI_ISL_263845	H1N2	Europe	swine
A/swine/Denmark/2017_10640_2_2p1/2017	EAS (1C.2.1)	EPI_ISL_4081372	H1N2	Europe	swine
A/swine/Denmark/2017_11771_1_1p1/2017	EAS (1C.2.1)	EPI_ISL_4081364	H1N2	Europe	swine
A/swine/Italy/18-170704/2018	EAS (1C.2.1)	EPI_ISL_4083721	H1N2	Europe	swine
A/swine/Czech_Republic/12244/2023	EAS (1C.2.1)	EPI_ISL_18257787	H1N1	Europe	swine
A/swine/Pskov_Region/RII-36S/2022	EAS (1C.2.1)	EPI_ISL_13699581	H1N2	Europe	swine
A/swine/Pskov_region/RII-41-2/2019	EAS (1C.2.1)	EPI_ISL_906136	H1N2	Europe	swine
A/swine/Spain/06001/2019	EAS (1C.2.1)	EPI_ISL_9593377	H1N2	Europe	swine
A/swine/Germany/2022AI00322/2022	EAS (1C.2.1)	EPI_ISL_17646280	H1N2	Europe	swine
A/swine/Germany/2022AI00342/2022	EAS (1C.2.1)	EPI_ISL_17646278	H1N2	Europe	swine
A/swine/Sellingen/19342/2013	EAS (1C.2.1)	EPI_ISL_505301	H1N2	Europe	swine
A/swine/Italy/228721/2017	EAS (1C.2.1)	EPI_ISL_14761691	H1N2	Europe	swine
A/swine/Denmark/2013_10_1545_1p1/2013	EAS (1C.2.1)	EPI_ISL_4081399	H1N1	Europe	swine
A/swine/Denmark/10-1391-3/2011	EAS (1C.2.1)	EPI_ISL_195111	H1N2	Europe	swine
A/swine/Denmark/10-2526-1/2011	EAS (1C.2.1)	EPI_ISL_195046	H1N1	Europe	swine
A/swine/Germany-NI/R369/09	EAS (1C.2.1)	EPI_ISL_79310	H1N2	Europe	swine
A/swine/Denmark/2014_10_28_1p1/2014	EAS (1C.2.1)	EPI_ISL_4081356	H1N2	Europe	swine
A/swine/Denmark/10-1958-1/2011	EAS (1C.2.1)	EPI_ISL_195045	H1N2	Europe	swine
A/swine/Denmark/2016_17805_2p1/2016	EAS (1C.2.1)	EPI_ISL_4081358	H1N2	Europe	swine
A/swine/France/59-200121/2020	EAS (1C.2.1)	EPI_ISL_9593331	H1N2	Europe	swine
A/swine/Denmark/2016_10856_3p1/2016	EAS (1C.2.1)	EPI_ISL_4081262	H1N1	Europe	swine
A/swine/Zeitlarn/4287/2005	EAS (1C.2.1)	EPI_ISL_504570	H1N1	Europe	swine
A/swine/Spain/6370-5/2018	EAS (1C.2.1)	EPI_ISL_9593372	H1N2	Europe	swine
A/swine/Serbia/7/2017	EAS (1C.2.1)	EPI_ISL_501507	H1N1	Europe	swine
A/swine/Balge/12670/2010	EAS (1C.2.1)	EPI_ISL_504723	H1N1	Europe	swine
A/swine/Netherlands/AR3009/2016	EAS (1C.2.1)	EPI_ISL_304089	H1N1	Europe	swine
A/swine/Germany/2021AI05981/2021	EAS (1C.2.1)	EPI_ISL_17646290	H1N1	Europe	swine
A/swine/Brilon/12263-2/2010	EAS (1C.2.1)	EPI_ISL_504706	H1N1	Europe	swine
A/swine/Czech_Republic/25-18/2017	EAS (1C.2.1)	EPI_ISL_304092	H1N1	Europe	swine

A/swine/Bad_Rothenfelde/4255/2005	EAS (1C.2.1)	EPI_ISL_504567	H1N1	Europe	swine
A/swine/Dornburg/10061/2009	EAS (1C.2.1)	EPI_ISL_504682	H1N1	Europe	swine
A/swine/Belgium/Glabbeek-287/2012	EAS (1C.2.1)	EPI_ISL_195144	H1N1	Europe	swine
A/swine/Greven/IDT2889/2004	EAS (1C.2.1)	EPI_ISL_29116	H1N1	Europe	swine
A/swine/Geldern/IDT2888/2004	EAS (1C.2.1)	EPI_ISL_29114	H1N1	Europe	swine
A/swine/Melle/5003/2006	EAS (1C.2.1)	EPI_ISL_504588	H1N1	Europe	swine
A/swine/Steinhorst/5129/2006	EAS (1C.2.1)	EPI_ISL_504592	H1N1	Europe	swine
A/swine/Germany/R312/2015	EAS (1C.2.1)	EPI_ISL_245748	H1N1	Europe	swine
A/swine/Buch/14162/2011	EAS (1C.2.1)	EPI_ISL_504766	H1N1	Europe	swine
A/swine/Italy/208724/2015	EAS (1C.2.1)	EPI_ISL_278822	H1N1	Europe	swine
A/swine/Moenchengladbach/21124/2015	EAS (1C.2.1)	EPI_ISL_504858	H1N1	Europe	swine
A/swine/Netherlands/Gent-62/2015	EAS (1C.2.1)	EPI_ISL_4081789	H1N1	Europe	swine
A/swine/Neudoerpen/9185/2009	EAS (1C.2.1)	EPI_ISL_504670	H1N1	Europe	swine
A/swine/Spain/40217-1/2020	EAS (1C.2.1)	EPI_ISL_18921583	H1N2	Europe	swine
A/swine/Spain/40240/2019	EAS (1C.2.1)	EPI_ISL_9593381	H1N1	Europe	swine
A/swine/Spain/33903/2012	EAS (1C.2.1)	EPI_ISL_195173	H1N1	Europe	swine
A/swine/Spain/44490-1/2020	EAS (1C.2.1)	EPI_ISL_6781259	H1N2	Europe	swine
A/swine/Italy/327669/2013	EAS (1C.2.1)	EPI_ISL_278899	H1N2	Europe	swine
A/swine/Italy/317775/2010	EAS (1C.2.1)	EPI_ISL_195019	H1N1	Europe	swine
A/swine/Italy/181051/2017	EAS (1C.2.1)	EPI_ISL_14761173	H1N2	Europe	swine
A/swine/Italy/333140/2014	EAS (1C.2.1)	EPI_ISL_278853	H1N2	Europe	swine
A/swine/Italy/302597/2017	EAS (1C.2.1)	EPI_ISL_14762862	H1N2	Europe	swine
A/swine/Italy/154890-2/2015	EAS (1C.2.1)	EPI_ISL_278829	H1N1	Europe	swine
A/swine/Italy/311368/2013	EAS (1C.2.1)	EPI_ISL_278897	H1N1	Europe	swine
A/swine/Italy/122595/2012	EAS (1C.2.1)	EPI_ISL_278926	H1N1	Europe	swine
A/swine/Italy/5033/2017	EAS (1C.2.1)	EPI_ISL_14755016	H1N1	Europe	swine
A/swine/Raddestorf/12564/2010	EAS (1C.2.1)	EPI_ISL_504719	H1N1	Europe	swine
A/swine/Italy/244784/2014	EAS (1C.2.1)	EPI_ISL_278860	H1N1	Europe	swine
A/swine/Italy/98113/2017	EAS (1C.2.1)	EPI_ISL_14755007	H1N1	Europe	swine
A/swine/Italy/87590/2017	EAS (1C.2.1)	EPI_ISL_14755010	H1N1	Europe	swine
A/swine/Italy/98240/2017	EAS (1C.2.1)	EPI_ISL_14755006	H1N1	Europe	swine
A/swine/Italy/19-49885-4/2019	EAS (1C.2.1)	EPI_ISL_4083716	H1N1	Europe	swine
A/swine/Italy/218884-2/2012	EAS (1C.2.1)	EPI_ISL_142229	H1N1	Europe	swine
A/swine/Italy/17-66671-3/2017	EAS (1C.2.1)	EPI_ISL_4083724	H1N1	Europe	swine
A/Pavia/65/2016	EAS (1C.2.1)	EPI_ISL_159427	H1N1	Europe	human
A/swine/Spain/32738/2012	EAS (1C.2.1)	EPI_ISL_195061	H1N1	Europe	swine
A/swine/Spain/50001-6/2019	EAS (1C.2.1)	EPI_ISL_6781229	H1N2	Europe	swine
A/swine/Austria/263-05/2005	EAS (1C.2.1)	EPI_ISL_18592208	H1N1	Europe	swine
A/swine/Lohne/14766/2012	EAS (1C.2.1)	EPI_ISL_504796	H1N1	Europe	swine
A/swine/Ostbevern/12867-2/2011	EAS (1C.2.1)	EPI_ISL_504730	H1N1	Europe	swine
A/swine/Spain/21290/2019	EAS (1C.2.1)	EPI_ISL_9593376	H1N1	Europe	swine
A/swine/Spain/45690-8/2018	EAS (1C.2.1)	EPI_ISL_9594274	H1N1	Europe	swine
A/swine/Spain/45690-4/2018	EAS (1C.2.1)	EPI_ISL_9594283	H1N2	Europe	swine
A/swine/Spain/000-1/2019	EAS (1C.2.1)	EPI_ISL_6781344	H1N1	Europe	swine
A/swine/Spain/45700/2019	EAS (1C.2.1)	EPI_ISL_9593379	H1N2	Europe	swine
A/swine/Spain/50547/2019	EAS (1C.2.1)	EPI_ISL_9593373	H1N2	Europe	swine
A/swine/Spain/25211-1/2020	EAS (1C.2.1)	EPI_ISL_18921578	H1N1	Europe	swine
A/swine/Spain/50001-2/2019	EAS (1C.2.1)	EPI_ISL_6781289	H1N2	Europe	swine
A/swine/Neuharlingersiel/14624-3/2012	EAS (1C.2.1)	EPI_ISL_504785	H1N1	Europe	swine
A/swine/Ringleben/20128/2014	EAS (1C.2.1)	EPI_ISL_505316	H1N2	Europe	swine
A/swine/Serbia/5/2016	EAS (1C.2.1)	EPI_ISL_501505	H1N1	Europe	swine
A/swine/Serbia/1/2017	EAS (1C.2.1)	EPI_ISL_501501	H1N1	Europe	swine
A/swine/Denmark/1085-1/2012	EAS (1C.2.1)	EPI_ISL_195052	H1N1	Europe	swine
A/swine/Lohne/12330/2010	EAS (1C.2.1)	EPI_ISL_504709	H1N1	Europe	swine
A/swine/France/44-200018/2020	EAS (1C.2.1)	EPI_ISL_9593301	H1N1	Europe	swine
A/swine/Warstein/13455/2011	EAS (1C.2.1)	EPI_ISL_504739	H1N1	Europe	swine
A/swine/Spain/32070-1/2020	EAS (1C.2.1)	EPI_ISL_18921582	H1N1	Europe	swine
A/swine/Italy/20-5709-1/2020	EAS (1C.2.1)	EPI_ISL_4083730	H1N1	Europe	swine
A/swine/Georgsmarienhuette/13824-2/2011	EAS (1C.2.1)	EPI_ISL_504754	H1N1	Europe	swine
A/swine/Iserlohn/11287/2010	EAS (1C.2.1)	EPI_ISL_504695	H1N1	Europe	swine
A/swine/France/35-170233/2017	EAS (1C.2.1)	EPI_ISL_502493	H1N1	Europe	swine
A/swine/France/50-170323/2017	EAS (1C.2.1)	EPI_ISL_502507	H1N1	Europe	swine
A/swine/France/22-200232/2020	EAS (1C.2.1)	EPI_ISL_9591701	H1N2	Europe	swine
A/swine/France/35-160197/2016	EAS (1C.2.1)	EPI_ISL_502490	H1N1	Europe	swine
A/swine/France/85-200222/2020	EAS (1C.2.1)	EPI_ISL_9593354	H1N1	Europe	swine
A/swine/France/29-200272/2020	EAS (1C.2.1)	EPI_ISL_9593278	H1N1	Europe	swine
A/swine/France/29-190583/2019	EAS (1C.2.1)	EPI_ISL_9593217	H1N1	Europe	swine
A/swine/France/29-190501/2019	EAS (1C.2.1)	EPI_ISL_9593211	H1N1	Europe	swine
A/swine/France/22-170192/2017	EAS (1C.2.1)	EPI_ISL_502459	H1N1	Europe	swine
A/swine/France/56-170084/2017	EAS (1C.2.1)	EPI_ISL_502512	H1N1	Europe	swine

A/swine/France/29-190437/2019	EAS (1C.2.1)	EPI_ISL_9593203	H1N1	Europe	swine
A/swine/France/22-180017/2018	EAS (1C.2.1)	EPI_ISL_502463	H1N1	Europe	swine
A/swine/France/29-200104/2020	EAS (1C.2.1)	EPI_ISL_9593221	H1N1	Europe	swine
A/swine/France/29-190539/2019	EAS (1C.2.1)	EPI_ISL_9593213	H1N1	Europe	swine
A/swine/Schwedeneck/21724/2015	EAS (1C.2.1)	EPI_ISL_504869	H1N1	Europe	swine
A/swine/Drensteinfurt/19376/2013	EAS (1C.2.1)	EPI_ISL_504828	H1N1	Europe	swine
A/swine/Czech_Republic/13240/2017	EAS (1C.2.1)	EPI_ISL_282135	H1N1	Europe	swine
A/swine/Poland/18275/2012	EAS (1C.2.1)	EPI_ISL_195266	H1N1	Europe	swine
A/swine/Viersen/13836/2011	EAS (1C.2.1)	EPI_ISL_504755	H1N1	Europe	swine
A/swine/Harreveld/21082/2014	EAS (1C.2.1)	EPI_ISL_504854	H1N1	Europe	swine
A/swine/Wetedorf/14350/2011	EAS (1C.2.1)	EPI_ISL_504773	H1N1	Europe	swine
A/swine/Aulendorf/12502/2010	EAS (1C.2.1)	EPI_ISL_504717	H1N1	Europe	swine
A/swine/Netherlands/Hoogeloon-167C/2012	EAS (1C.2.1)	EPI_ISL_195145	H1N1	Europe	swine
A/swine/Germany/R1181/2014	EAS (1C.2.1)	EPI_ISL_245703	H1N1	Europe	swine
A/swine/Belgium/Heist-op-den-Berg-363/2012	EAS (1C.2.1)	EPI_ISL_195146	H1N1	Europe	swine
A/swine/Belgorod_region/RII-64-3S/2023	EAS (1C.2.1)	EPI_ISL_18837244	H1N1	Europe	swine
A/swine/Ankum/13894/2011	EAS (1C.2.1)	EPI_ISL_504757	H1N1	Europe	swine
A/swine/Glabbeek/21006/2014	EAS (1C.2.1)	EPI_ISL_504851	H1N1	Europe	swine
A/swine/Belgium/Gent-241/2018	EAS (1C.2.1)	EPI_ISL_4081784	H1N1	Europe	swine
A/swine/France/01-140489/2014	EAS (1C.2.1)	EPI_ISL_502445	H1N1	Europe	swine
A/swine/France/64-140385/2014	EAS (1C.2.1)	EPI_ISL_502540	H1N1	Europe	swine
A/swine/France/61-140350/2014	EAS (1C.2.1)	EPI_ISL_502535	H1N2	Europe	swine
A/swine/France/22-180018/2018	EAS (1C.2.1)	EPI_ISL_502464	H1N1	Europe	swine
A/swine/France/35-200008/2019	EAS (1C.2.1)	EPI_ISL_9593284	H1N1	Europe	swine
A/swine/France/22-170307/2017	EAS (1C.2.1)	EPI_ISL_502460	H1N1	Europe	swine
A/swine/France/22-110529/2011	EAS (1C.2.1)	EPI_ISL_195080	H1N2	Europe	swine
A/swine/France/Cotes_dArmor-0455/2011	EAS (1C.2.1)	EPI_ISL_502585	H1N1	Europe	swine
A/swine/France/61-150219/2015	EAS (1C.2.1)	EPI_ISL_502536	H1N2	Europe	swine
A/swine/France/56-170226/2017	EAS (1C.2.1)	EPI_ISL_502516	H1N1	Europe	swine
A/swine/France/29-160352/2016	EAS (1C.2.1)	EPI_ISL_502476	H1N1	Europe	swine
A/swine/France/22-200290/2020	EAS (1C.2.1)	EPI_ISL_9593187	H1N1	Europe	swine
A/swine/France/05-200058/2020	EAS (1C.2.1)	EPI_ISL_9589893	H1N1	Europe	swine
A/swine/France/22-170027/2017	EAS (1C.2.1)	EPI_ISL_502457	H1N1	Europe	swine
A/swine/France/56-170177/2017	EAS (1C.2.1)	EPI_ISL_502515	H1N1	Europe	swine
A/swine/France/50-120350/2012	EAS (1C.2.1)	EPI_ISL_195154	H1N1	Europe	swine
A/swine/France/53-170144/2017	EAS (1C.2.1)	EPI_ISL_502510	H1N1	Europe	swine
A/swine/France/29-160311/2016	EAS (1C.2.1)	EPI_ISL_502475	H1N1	Europe	swine
A/swine/France/22-180008/2018	EAS (1C.2.1)	EPI_ISL_502462	H1N1	Europe	swine
A/swine/France/50-130337/2013	EAS (1C.2.1)	EPI_ISL_502504	H1N1	Europe	swine
A/swine/France/29-160215/2016	EAS (1C.2.1)	EPI_ISL_502578	H1N1	Europe	swine
A/swine/Netherlands/Groenlo-37/2012	EAS (1C.2.1)	EPI_ISL_195025	H1N1	Europe	swine
A/swine/Belgium/Poperinge-387/2011	EAS (1C.2.1)	EPI_ISL_195028	H1N1	Europe	swine
A/swine/France/59_180334/2018	EAS (1C.2.1)	EPI_ISL_502534	H1N1	Europe	swine
A/Swine/Netherlands/GDE0036/2023	EAS (1C.2.1)	EPI_ISL_18801995	H1N1	Europe	swine
A/Swine/Netherlands/GDE0061/2023	EAS (1C.2.1)	EPI_ISL_18802014	H1N1	Europe	swine
A/Swine/Netherlands/GDE0087/2023	EAS (1C.2.1)	EPI_ISL_18802205	H1N1	Europe	swine
A/swine/France/59-190568/2020	EAS (1C.2.1)	EPI_ISL_9593330	H1N1	Europe	swine
A/swine/Belgium/Gent-05/2019	EAS (1C.2.1)	EPI_ISL_4081775	H1N1	Europe	swine
A/swine/Germany/2021AI08854/2021	EAS (1C.2.1)	EPI_ISL_17646282	H1N1	Europe	swine
A/swine/Rhede/21178/2015	EAS (1C.2.1)	EPI_ISL_504861	H1N1	Europe	swine
A/swine/Illinois/02757/2009	HSS (1B.2.1)	EPI_ISL_150940	H1N1	North America	swine
A/swine/North_Carolina/SG1335/2009	HSS (1B.2.1)	EPI_ISL_150935	H1N2	North America	swine
A/swine/North_Carolina/CL1203/2018	HSS (1B.2.1)	EPI_ISL_14769977	H1N2	North America	swine
A/swine/North_Carolina/02249/2008	HSS (1B.2.1)	EPI_ISL_150921	H1N2	North America	swine
A/swine/Mexico/GtoDMZC01/2014	HSS (1B.2.1)	EPI_ISL_234602	H1N2	North America	swine
A/swine/Kentucky/02564/2009	HSS (1B.2.1)	EPI_ISL_150932	H1N2	North America	swine
A/swine/Virginia/SG1386/2011	HSS (1B.2.1)	EPI_ISL_150995	H1N2	North America	swine
A/swine/Pennsylvania/A01944215/2015	HSS (1B.2.1)	EPI_ISL_203194	H1N2	North America	swine
A/swine/North_Carolina/A01290777/2013	HSS (1B.2.1)	EPI_ISL_153757	H1N2	North America	swine
A/swine/North_Carolina/1565521/2015	HSS (1B.2.1)	EPI_ISL_4082090	H1N2	North America	swine
A/swine/Ohio/OH-17-25445/2017	HSS (1B.2.1)	EPI_ISL_388921	H1N1	North America	swine
A/swine/Pennsylvania/A02636085/2021	HSS (1B.2.1)	EPI_ISL_6795220	H1N2	North America	swine
A/swine/Iowa/17TOSU3333/2017	HSS (1B.2.1)	EPI_ISL_373550	H1N2	North America	swine
A/swine/Indiana/A01932256/2017	HSS (1B.2.1)	EPI_ISL_260805	H1N2	North America	swine
A/swine/North_Carolina/A01728820/2015	HSS (1B.2.1)	EPI_ISL_211825	H1N2	North America	swine
A/swine/Indiana/A01894974/2016	HSS (1B.2.1)	EPI_ISL_222687	H1N2	North America	swine
A/swine/North_Carolina/A02245213/2019	HSS (1B.2.1)	EPI_ISL_393502	H1N1	North America	swine
A/swine/Iowa/A02524417/2020	HSS (1B.2.1)	EPI_ISL_499222	H1N2	North America	swine
A/swine/Ohio/OH-21613/2021	HSS (1B.2.1)	EPI_ISL_17471886	H1N2	North America	swine

A/swine/Ohio/18TOSU4084/2018	HSS (1B.2.1)	EPI_ISL_399663	H1N2	North America	swine
A/swine/Ohio/A02636465/2022	HSS (1B.2.1)	EPI_ISL_14775610	H1N2	North America	swine
A/swine/Indiana/A02635956/2021	HSS (1B.2.1)	EPI_ISL_4089889	H1N2	North America	swine
A/swine/Iowa/SJCEIRR-SU1504/2023	HSS (1B.2.1)	EPI_ISL_18755193	H1N2	North America	swine
A/swine/Minnesota/SG-00239/2007	HSS (1B.2.2)	EPI_ISL_25306	H1N2	North America	swine
A/swine/Texas/008648/2008	HSS (1B.2.2)	EPI_ISL_63210	H1N2	North America	swine
A/swine/Oklahoma/A01203865/2012	HSS (1B.2.2)	EPI_ISL_129802	H1N2	North America	swine
A/swine/Missouri/A01444664/2013	HSS (1B.2.2.2)	EPI_ISL_136654	H1N2	North America	swine
A/swine/Illinois/A01944807/2015	HSS (1B.2.2.2)	EPI_ISL_203138	H1N2	North America	swine
A/swine/Oklahoma/A01202498/2011	HSS (1B.2.2.2)	EPI_ISL_127770	H1N2	North America	swine
A/swine/Nebraska/A01300150/2012	HSS (1B.2.2.2)	EPI_ISL_130123	H1N2	North America	swine
A/swine/Minnesota/A01943452/2016	HSS (1B.2.2.2)	EPI_ISL_226061	H1N2	North America	swine
A/swine/Nebraska/A01202501/2011	HSS (1B.2.2.2)	EPI_ISL_127771	H1N2	North America	swine
A/swine/Missouri/A01444795/2013	HSS (1B.2.2.2)	EPI_ISL_137561	H1N2	North America	swine
A/swine/Oklahoma/A01202415/2011	HSS (1B.2.2.2)	EPI_ISL_127756	H1N2	North America	swine
A/swine/Oklahoma/A01290605/2013	HSS (1B.2.2.2)	EPI_ISL_150673	H1N1	North America	swine
A/swine/Oklahoma/A01672680/2017	HSS (1B.2.2.2)	EPI_ISL_257154	H1N1	North America	swine
A/swine/South_Dakota/A01678606/2019	HSS (1B.2.2.2)	EPI_ISL_393525	H1N2	North America	swine
A/swine/Iowa/A02636039/2021	HSS (1B.2.2.2)	EPI_ISL_6781458	H1N2	North America	swine
A/swine/Oklahoma/A02245291/2019	HSS (1B.2.2.2)	EPI_ISL_399433	H1N1	North America	swine
A/swine/Minnesota/A02478597/2019	HSS (1B.2.2.2)	EPI_ISL_388670	H1N2	North America	swine
A/swine/Minnesota/A01047913/2010	HSS (1B.2.2.1)	EPI_ISL_119335	H1N2	North America	swine
A/swine/Missouri/A01411322/2014	HSS (1B.2.2.1)	EPI_ISL_157196	H1N2	North America	swine
A/swine/Nebraska/A01104068/2013	HSS (1B.2.2.1)	EPI_ISL_153861	H1N2	North America	swine
A/swine/Michigan/A01671231/2016	HSS (1B.2.2.1)	EPI_ISL_253645	H1N2	North America	swine
A/swine/Iowa/A02134236/2017	HSS (1B.2.2.1)	EPI_ISL_296426	H1N2	North America	swine
A/swine/Nebraska/A01727959/2015	HSS (1B.2.2.1)	EPI_ISL_209199	H1N2	North America	swine
A/swine/Iowa/A01668210/2016	HSS (1B.2.2.1)	EPI_ISL_246032	H1N2	North America	swine
A/swine/Texas/A02245132/2019	HSS (1B.2.2.1)	EPI_ISL_368783	H1N2	North America	swine
A/swine/Illinois/TM1702DTW22/2017	HSS (1B.2.2.1)	EPI_ISL_282414	H1N2	North America	swine
A/swine/Minnesota/USDA-A02978581/2023	HSS (1B.2.2.1)	EPI_ISL_18758905	H1N2	North America	swine
A/swine/Iowa/A02525361/2021	HSS (1B.2.2.1)	EPI_ISL_4083562	H1N2	North America	swine
A/swine/Wyoming/A02636435/2022	HSS (1B.2.2.1)	EPI_ISL_14775605	H1N2	North America	swine
A/swine/Illinois/A02478528/2019	HSS (1B.2.2.1)	EPI_ISL_372420	H1N2	North America	swine
A/swine/Minnesota/A02636074/2021	HSS (1B.2.2.1)	EPI_ISL_6781671	H1N2	North America	swine
A/swine/Indiana/A02636150/2021	HSS (1B.2.2.1)	EPI_ISL_6795300	H1N2	North America	swine
A/swine/Iowa/A02524419/2020	HSS (1B.2.2.1)	EPI_ISL_499225	H1N2	North America	swine
A/swine/Ploufragan/0214/2006	HSS (1B.1.2.1)	EPI_ISL_129530	H1N2	Europe	swine
A/swine/England/1437/2004	HSS (1B.1.2.1)	EPI_ISL_195117	H1N2	Europe	swine
A/swine/England/002295/2002	HSS (1B.1.2.1)	EPI_ISL_129452	H1N2	Europe	swine
A/swine/England/000304/2009	HSS (1B.1.2.1)	EPI_ISL_195120	H1N2	Europe	swine
A/swine/England/7856/2012	HSS (1B.1.2.1)	EPI_ISL_195097	H1N2	Europe	swine
A/swine/England/044649/2022	HSS (1B.1.2.1)	EPI_ISL_18586951	H1N2	Europe	swine
A/swine/England/058879/2011	HSS (1B.1.2.1)	EPI_ISL_195039	H1N2	Europe	swine
A/swine/Poland/T2/2008	HSS (1B.1.2.1)	EPI_ISL_505169	H1N2	Europe	swine
A/swine/England/1382/2010	HSS (1B.1.2.1)	EPI_ISL_88197	H1N2	Europe	swine
A/swine/England/483/2006	HSS (1B.1.2.1)	EPI_ISL_129491	H1N2	Europe	swine
A/swine/England/43142/2001	HSS (1B.1.2.1)	EPI_ISL_129488	H1N2	Europe	swine
A/swine/England/303/2009	HSS (1B.1.2.1)	EPI_ISL_129477	H1N2	Europe	swine
A/swine/England/60797/2000	HSS (1B.1.2.1)	EPI_ISL_129493	H1N2	Europe	swine
A/swine/England/038712/2012	HSS (1B.1.2.1)	EPI_ISL_195101	H1N2	Europe	swine
A/swine/England/247909/2022	HSS (1B.1.2.1)	EPI_ISL_18586961	H1N2	Europe	swine
A/swine/England/245754/2022	HSS (1B.1.2.1)	EPI_ISL_18586959	H1N2	Europe	swine
A/swine/England/260038/2023	HSS (1B.1.2.1)	EPI_ISL_18586962	H1N2	Europe	swine
A/swine/England/661263/1997	HSS (1B.1.2)	EPI_ISL_129498	H1N2	Europe	swine
A/swine/Scotland/410440/94	HSS (1B.1.2)	EPI_ISL_1113	H1N2	Europe	swine
A/swine/Spain/29262/2012	HSS (1B.1.1)	EPI_ISL_195113	H1N2	Europe	swine
A/swine/Spain/41566-1/2021	HSS (1B.1.1)	EPI_ISL_18921585	H1N2	Europe	swine
A/swine/Spain/50001-5/2019	HSS (1B.1.1)	EPI_ISL_6781292	H1N2	Europe	swine
A/swine/Spain/29257/2012	HSS (1B.1.1)	EPI_ISL_195060	H1N2	Europe	swine
A/swine/Spain/25001-3/2019	HSS (1B.1.1)	EPI_ISL_6781291	H1N2	Europe	swine
A/swine/Spain/24237/2011	HSS (1B.1.1)	EPI_ISL_195057	H1N2	Europe	swine
A/swine/Spain/22270-2/2020	HSS (1B.1.1)	EPI_ISL_18921577	H1N2	Europe	swine
A/swine/Italy/3592/1999	HSS (1B.1.1)	EPI_ISL_95866	H1N2	Europe	swine
A/swine/Italy/172336/2001	HSS (1B.1.1)	EPI_ISL_129525	H1N1	Europe	swine
A/swine/France/Cotes_dArmor-0295/2011	HSS (1B.1.1)	EPI_ISL_502584	H1N2	Europe	swine
A/swine/France/Ille_et_Vilaine-0648/2011	HSS (1B.1.1)	EPI_ISL_502588	H1N2	Europe	swine
A/swine/France/Cotes_dArmor-0070/2010	HSS (1B.1.1)	EPI_ISL_502551	H1N1	Europe	swine
A/swine/France/35-120024/2012	HSS (1B.1.1)	EPI_ISL_195077	H1N2	Europe	swine
A/swine/France/Finistere-0186/2011	HSS (1B.1.1)	EPI_ISL_502586	H1N2	Europe	swine

A/swine/France/22-150384/2015	HSS (1B.1.1)	EPI_ISL_502456	H1N2	Europe	swine
A/swine/France/29-170091/2017	HSS (1B.1.1)	EPI_ISL_502479	H1N2	Europe	swine
A/swine/France/29-160400/2016	HSS (1B.1.1)	EPI_ISL_502477	H1N2	Europe	swine
A/swine/Italy/259543/2003	HSS (1B.1.1)	EPI_ISL_95862	H1N2	Europe	swine
A/swine/Gent/100/2007	HSS (1B.1.1)	EPI_ISL_129558	H1N2	Europe	swine
A/swine/Natingen/13545/2011	HSS (1B.1.1)	EPI_ISL_505210	H1N2	Europe	swine
A/swine/Belgium/Oostkamp-26/2012	HSS (1B.1.1)	EPI_ISL_195037	H1N2	Europe	swine
A/swine/Emstek/10466/2009	HSS (1B.1.1)	EPI_ISL_505180	H1N2	Europe	swine
A/swine/Italy/57680/2011	HSS (1B.1.1)	EPI_ISL_195017	H1N2	Europe	swine
A/swine/Bad_Wuennenberg/6405/2007	HSS (1B.1.1)	EPI_ISL_505159	H1N2	Europe	swine
A/swine/Werl/21305/2015	HSS (1B.1.1)	EPI_ISL_505336	H1N2	Europe	swine
A/swine/Czech_Republic/17690/2018	HSS (1B.1.1)	EPI_ISL_18146522	H1N2	Europe	swine
A/swine/Dahlheim/19138/2013	HSS (1B.1.1)	EPI_ISL_505291	H1N2	Europe	swine
A/swine/Rosendahl/13572/2011	HSS (1B.1.1)	EPI_ISL_505211	H1N2	Europe	swine
A/swine/Lohne/14151/2011	HSS (1B.1.1)	EPI_ISL_505224	H1N2	Europe	swine
A/swine/Essen/20452/2014	HSS (1B.1.1)	EPI_ISL_504839	H1N1	Europe	swine
A/swine/Kuehlenthal/17963/2013	HSS (1B.1.1)	EPI_ISL_505279	H1N2	Europe	swine
A/swine/Lohne/15102/2012	HSS (1B.1.1)	EPI_ISL_505244	H1N2	Europe	swine
A/swine/Germany/2022AI00361/2022	HSS (1B.1.1)	EPI_ISL_17646273	H1N2	Europe	swine
A/Swine/Netherlands/GDE0057/2022	HSS (1B.1.1)	EPI_ISL_18802199	H1N2	Europe	swine
A/swine/Germany/2021AI08934/2021	HSS (1B.1.1)	EPI_ISL_17646281	H1N2	Europe	swine
A/Swine/Netherlands/GDE0098/2023	HSS (1B.1.1)	EPI_ISL_18802189	H1N2	Europe	swine
A/swine/Spain/31310-1/2020	HSS (1B.1.1)	EPI_ISL_18921581	H1N2	Europe	swine
A/swine/Belgium/Gent-178/2019	HSS (1B.1.1)	EPI_ISL_4081736	H1N2	Europe	swine
A/swine/France/59-190057/2019	HSS (1B.1.1)	EPI_ISL_9593328	H1N2	Europe	swine
A/swine/Ohio/23/1935	CS (1A.1.1)	EPI_ISL_7899	H1N1	North America	swine
A/swine/Wisconsin/1/61	CS (1A.1.1)	EPI_ISL_1120	H1N1	North America	swine
A/swine/Illinois/1/1975	CS (1A.1.1)	EPI_ISL_7124	H1N1	North America	swine
A/swine/Arizona/148/1977	CS (1A.1.1)	EPI_ISL_7537	H1N1	North America	swine
A/swine/Tennessee/25/1977	CS (1A.1.1)	EPI_ISL_5206	H1N1	North America	swine
A/swine/Niigata/1/1977	CS (1A.1.1)	EPI_ISL_28432	H1N1	Japan	swine
A/swine/England/117316/1986	CS (1A.1.1)	EPI_ISL_153501	H1N1	Europe	swine
A/swine/Okinawa/2/2005	CS (1A.1.1)	EPI_ISL_77730	H1N1	Japan	swine
A/swine/Miyazaki/1/2006	CS (1A.1.1)	EPI_ISL_20099	H1N2	Japan	swine
A/swine/Tochigi/1/2008	CS (1A.1.1)	EPI_ISL_237796	H1N2	Japan	swine
A/swine/Miyagi/3/2003	CS (1A.1.1)	EPI_ISL_379211	H1N2	Japan	swine
A/swine/Niigata/729/2004	CS (1A.1.1)	EPI_ISL_90157	H1N2	Japan	swine
A/swine/Chiba/1/1991	CS (1A.1.1)	EPI_ISL_76997	H1N2	Japan	swine
A/swine/Saitama/1/2000	CS (1A.1.1)	EPI_ISL_90158	H1N2	Japan	swine
A/swine/Saitama/1996	CS (1A.1.1)	EPI_ISL_68696	H1N2	Japan	swine
A/swine/Tochigi/38-7119/2019	CS (1A.1.1)	EPI_ISL_369287	H1N2	Japan	swine
A/swine/Gunma/SVB-160-1/2017	CS (1A.1.1)	EPI_ISL_290897	H1N2	Japan	swine
A/swine/Chiba/30/2012	CS (1A.1.1)	EPI_ISL_181286	H1N1	Japan	swine
A/swine/Gunma/35-6817/2019	CS (1A.1.1)	EPI_ISL_365558	H1N2	Japan	swine
A/swine/Chiba/1-1532/2016	CS (1A.1.1)	EPI_ISL_233596	H1N2	Japan	swine
A/swine/Chiba/1-2690/2016	CS (1A.1.1)	EPI_ISL_237406	H1N1	Japan	swine
A/swine/Chiba/1-4957/2017	CS (1A.1.1)	EPI_ISL_281901	H1N1	Japan	swine
A/swine/Chiba/1/2014	CS (1A.1.1)	EPI_ISL_275577	H1N2	Japan	swine
A/swine/Saitama/21/2004	CS (1A.1.1)	EPI_ISL_90159	H1N2	Japan	swine
A/swine/Gunma/12-3700/2016	CS (1A.1.1)	EPI_ISL_237928	H1N2	Japan	swine
A/swine/Gunma/46-6975/2019	CS (1A.1.1)	EPI_ISL_365554	H1N2	Japan	swine
A/swine/Gunma/SVB38/2016	CS (1A.1.1)	EPI_ISL_268776	H1N2	Japan	swine
A/swine/Gunma/09/2014	CS (1A.1.1)	EPI_ISL_274846	H1N2	Japan	swine
A/swine/Gunma/35-5573/2018	CS (1A.1.1)	EPI_ISL_304107	H1N2	Japan	swine
A/swine/Gunma/11-2902/2016	CS (1A.1.1)	EPI_ISL_255371	H1N2	Japan	swine
A/swine/Chiba/14/2012	CS (1A.1.1)	EPI_ISL_181291	H1N2	Japan	swine
A/swine/Chiba/107/2012	CS (1A.1.1)	EPI_ISL_181292	H1N2	Japan	swine
A/swine/Chiba/V662/2015	CS (1A.1.1)	EPI_ISL_233610	H1N2	Japan	swine
A/swine/Ibaraki/1/2019	CS (1A.1.1)	EPI_ISL_365550	H1N2	Japan	swine
A/swine/Chiba/1-956/2016	CS (1A.1.1)	EPI_ISL_221977	H1N2	Japan	swine
A/swine/Mie/R02/2012	CS (1A.1.1)	EPI_ISL_134588	H1N2	Japan	swine
A/swine/Chiba/40/2013	CS (1A.1.1)	EPI_ISL_274849	H1N2	Japan	swine
A/swine/Gunma/6-3177/2016	CS (1A.1.1)	EPI_ISL_237389	H1N2	Japan	swine
A/swine/Gunma/34-4898/2017	CS (1A.1.1)	EPI_ISL_281900	H1N2	Japan	swine
A/swine/Gunma/7-6448/2019	CS (1A.1.1)	EPI_ISL_348521	H1N2	Japan	swine
A/swine/Tochigi/1/2012	CS (1A.1.1)	EPI_ISL_237798	H1N2	Japan	swine
A/swine/Ibaraki/1/2015	CS (1A.1.1)	EPI_ISL_221976	H1N2	Japan	swine
A/swine/Tochigi/2/2018	CS (1A.1.1)	EPI_ISL_314819	H1N2	Japan	swine
A/swine/Chiba/1-6874/2019	CS (1A.1.1)	EPI_ISL_365289	H1N2	Japan	swine
A/swine/Hokkaido/1980	CS (1A.1.1)	EPI_ISL_379212	H1N1	Japan	swine

A/swine/Shimane/1/1978	CS (1A.1.1)	EPI_ISL_379213	H1N1	Japan	swine
A/swine/Saraburi/NIAH13021/2005	CS (1A.1.1)	EPI_ISL_13920	H1N2	South East Asia	swine
A/swine/Chonburi/NIAH977/2004	CS (1A.1.1)	EPI_ISL_28426	H1N1	South East Asia	swine
A/swine/Chonburi/NIAH589/2005	CS (1A.1.1)	EPI_ISL_13916	H1N1	South East Asia	swine
A/swine/Saraburi/NIAH100761-22/2009	CS (1A.1.1)	EPI_ISL_106912	H1N1	South East Asia	swine
A/swine/Thailand/CB069/2010	CS (1A.1.1)	EPI_ISL_146843	H1N1	South East Asia	swine
A/swine/Wisconsin/661/1980	CS (1A.1.1)	EPI_ISL_7138	H1N1	North America	swine
A/swine/Ontario/57561/03	CS (1A.1.1)	EPI_ISL_9877	H1N1	North America	swine
A/swine/Korea/VDS3/2009	CS (1A.1.1)	EPI_ISL_132827	H1N1	South Korea	swine
A/swine/QC/370/2009	CS (1A.1.1)	EPI_ISL_93161	H1N1	North America	swine
A/swine/Ontario/11112/04	CS (1A.1.1)	EPI_ISL_65306	H1N1	North America	swine
A/swine/Oklahoma/SG1458/2004	CS (1A.1.1)	EPI_ISL_151256	H1N1	North America	swine
A/swine/QC/1531-3/2009	CS (1A.1.1)	EPI_ISL_93172	H1N1	North America	swine
A/swine/Ontario/53518/03	CS (1A.1.1)	EPI_ISL_9880	H1N1	North America	swine
A/swine/QC/3399-6/2009	CS (1A.1.1)	EPI_ISL_93193	H1N1	North America	swine
A/swine/Alberta/SD0014/2013	CS (1A.1.1)	EPI_ISL_207169	H1N1	North America	swine
A/swine/Alberta/SD0164/2016	CS (1A.1.1)	EPI_ISL_282514	H1N2	North America	swine
A/swine/Saskatchewan/SD0056/2014	CS (1A.1.1)	EPI_ISL_207980	H1N1	North America	swine
A/swine/Manitoba/01781/2007	CS (1A.1.1)	EPI_ISL_151014	H1N1	North America	swine
A/SW/MB/5-5/2009	CS (1A.1.1)	EPI_ISL_93209	H1N1	North America	swine
A/swine/Alberta/SD0154/2016	CS (1A.1.1)	EPI_ISL_281247	H1N1	North America	swine
A/swine/Manitoba/D0342/2014	CS (1A.1.1)	EPI_ISL_207956	H1N2	North America	swine
A/swine/Manitoba/D0175/2012	CS (1A.1.1)	EPI_ISL_207053	H1N1	North America	swine
A/swine/Alberta/SD0042/2014	CS (1A.1.1)	EPI_ISL_207968	H1N2	North America	swine
A/swine/Iowa/A01796529/2015	CS (1A.1.1)	EPI_ISL_208974	H1N2	North America	swine
A/swine/Manitoba/D0523/2017	CS (1A.1.1)	EPI_ISL_378585	H1N2	North America	swine
A/swine/Indiana/22TOSU0234/2022	CS (1A.1.1)	EPI_ISL_17409686	H1N2	North America	swine
A/swine/Minnesota/A02245178/2019	CS (1A.1.1)	EPI_ISL_372560	H1N1	North America	swine
A/swine/Saskatchewan/SD0204/2016	CS (1A.1.1)	EPI_ISL_282448	H1N2	North America	swine
A/swine/Manitoba/SD0144/2016	CS (1A.1.1)	EPI_ISL_282543	H1N2	North America	swine
A/swine/Pennsylvania/A02478616/2019	CS (1A.1.1)	EPI_ISL_393491	H1N2	North America	swine
A/swine/Iowa/A02479262/2020	CS (1A.1.1)	EPI_ISL_502849	H1N2	North America	swine
A/swine/North_Carolina/A02635751/2021	CS (1A.1.1)	EPI_ISL_4083629	H1N2	North America	swine
A/swine/North_Carolina/A02245230/2019	CS (1A.1.1)	EPI_ISL_393524	H1N2	North America	swine
A/swine/Utah/A02478716/2019	CS (1A.1.1)	EPI_ISL_399397	H1N2	North America	swine
A/swine/Saskatchewan/SD0240/2017	CS (1A.1.1)	EPI_ISL_373103	H1N2	North America	swine
A/swine/Iowa/A02479351/2020	CS (1A.1.1)	EPI_ISL_501787	H1N2	North America	swine
A/swine/Kansas/A02636499/2022	CS (1A.1.1)	EPI_ISL_14777815	H1N2	North America	swine
A/swine/California/A01671936/2016	CS (1A.1.1)	EPI_ISL_253928	H1N2	North America	swine
A/swine/Illinois/A02479149/2020	CS (1A.1.1)	EPI_ISL_504087	H1N2	North America	swine
A/swine/South_Dakota/G76266/2022	CS (1A.1.1)	EPI_ISL_15620432	H1N2	North America	swine
A/swine/Kansas/A02636473/2022	CS (1A.1.1)	EPI_ISL_14777814	H1N2	North America	swine
A/swine/Indiana/SJCEIRR-SU1783/2023	CS (1A.1.1)	EPI_ISL_18752273	H1N1	North America	swine
A/swine/Kansas/A02246997/2021	CS (1A.1.1)	EPI_ISL_14769972	H1N2	North America	swine
A/swine/Iowa/1/1985	CS (1A.1.1)	EPI_ISL_7121	H1N1	North America	swine
A/swine/Ratchaburi/NIAH550/2003	CS (1A.1.1)	EPI_ISL_28428	H1N1	South East Asia	swine
A/swine/Thailand/CU-S3248N/2012	CS (1A.1.1)	EPI_ISL_156349	H1N1	South East Asia	swine
A/swine/Thailand/CU21304/2018	CS (1A.1.1)	EPI_ISL_696538	H1N1	South East Asia	swine
A/swine/Ratchaburi/NIAH1481/2000	CS (1A.1.1)	EPI_ISL_28425	H1N1	South East Asia	swine
A/swine/Thailand/CU-SPL4/2010	CS (1A.1.1)	EPI_ISL_146752	H1N1	South East Asia	swine
A/swine/Thailand/CB001/2009	CS (1A.1.1)	EPI_ISL_146867	H1N1	South East Asia	swine
A/swine/Beijing/156/1991	CS (1A.1.1)	EPI_ISL_148291	H1N1	China	swine
A/swine/Shanghai/3/2005	CS (1A.1.1)	EPI_ISL_29264	H1N1	China	swine
A/swine/Guangdong/553/2010	CS (1A.1.1)	EPI_ISL_96975	H1N2	China	swine
A/swine/Zhejiang/1/2004	CS (1A.1.1)	EPI_ISL_9538	H1N2	China	swine
A/swine/Tianjin/1/2007	CS (1A.1.1)	EPI_ISL_103792	H1N2	China	swine
A/swine/Tianjin/1/2004	CS (1A.1.1)	EPI_ISL_33834	H1N2	China	swine
A/swine/Guangdong/1/2012	CS (1A.1.1)	EPI_ISL_230761	H1N2	China	swine
A/swine/Fujian/12/2010	CS (1A.1.1)	EPI_ISL_168484	H1N2	China	swine
A/swine/Guangdong/1/2010	CS (1A.1.1)	EPI_ISL_97360	H1N2	China	swine
A/swine/Hubei/04/2009	CS (1A.1.1)	EPI_ISL_91639	H1N2	China	swine
A/swine/North_Carolina/47834/2000	CS (1A.2)	EPI_ISL_95807	H1N1	North America	swine
A/swine/Virginia/01359/2006	CS (1A.2)	EPI_ISL_150847	H1N1	North America	swine
A/swine/Kentucky/02116/2008	CS (1A.2)	EPI_ISL_150890	H1N1	North America	swine
A/swine/Ontario/DM_21/2017	CS (1A.2)	EPI_ISL_378993	H1N2	North America	swine
A/swine/Quebec/SD0175/2016	CS (1A.2)	EPI_ISL_282495	H1N1	North America	swine
A/SW/CO/17871/01	CS (1A.2)	EPI_ISL_3075	H1N2	North America	swine
A/swine/Iowa/H03HS5/2003	CS (1A.2)	EPI_ISL_83307	H1N1	North America	swine
A/Swine/Korea/CY02/02	CS (1A.2)	EPI_ISL_3106	H1N2	South Korea	swine
A/swine/Texas/01657/2007	CS (1A.2)	EPI_ISL_150872	H1N1	North America	swine

A/swine/Korea/CAN01/2004	CS (1A.2)	EPI_ISL_24571	H1N1	South Korea	swine
A/swine/Korea/CAS08/2005	CS (1A.2)	EPI_ISL_24572	H1N1	South Korea	swine
A/swine/Minnesota/SG1144/2006	CS (1A.2)	EPI_ISL_96930	H1N1	North America	swine
A/swine/Indiana/240218/2010	CS (1A.2)	EPI_ISL_88837	H1N2	North America	swine
A/swine/Iowa/02096/2008	CS (1A.2)	EPI_ISL_79852	H1N1	North America	swine
A/Minnesota/10/2009	CS (1A.2)	EPI_ISL_85649	H1N1	North America	human
A/swine/Mississippi/A01202593/2011	CS (1A.2)	EPI_ISL_127780	H1N1	North America	swine
A/swine/Korea/VDS1/2010	CS (1A.2)	EPI_ISL_132824	H1N2	South Korea	swine
A/swine/Korea/VDS4/2008	CS (1A.2)	EPI_ISL_132826	H1N1	South Korea	swine
A/swine/Nebraska/A01301493/2012	CS (1A.2)	EPI_ISL_130124	H1N1	North America	swine
A/swine/Nebraska/A01277241/2012	CS (1A.2)	EPI_ISL_132964	H1N1	North America	swine
A/swine/Nebraska/02013/2008	CS (1A.2)	EPI_ISL_79853	H1N1	North America	swine
A/swine/Minnesota/A01049462/2011	CS (1A.2)	EPI_ISL_93366	H1N1	North America	swine
A/swine/Mexico/8935602/2012	CS (1A.2)	EPI_ISL_196000	H1N1	North America	swine
A/swine/Missouri/A01432837/2013	CS (1A.2)	EPI_ISL_145746	H1N2	North America	swine
A/swine/Ohio/09SW1488E/2009	CS (1A.2)	EPI_ISL_133784	H1N2	North America	swine
A/swine/Oklahoma/SG1251/2010	CS (1A.2)	EPI_ISL_150941	H1N1	North America	swine
A/swine/Oklahoma/A01733315/2016	CS (1A.2)	EPI_ISL_220518	H1N1	North America	swine
A/swine/Oklahoma/A01671784/2016	CS (1A.2)	EPI_ISL_253932	H1N1	North America	swine
A/swine/Texas/A01785406/2018	CS (1A.2)	EPI_ISL_298632	H1N1	North America	swine
A/swine/Oklahoma/A01785752/2018	CS (1A.2)		H1N1	North America	swine
A/swine/Oklahoma/A02245094/2019	CS (1A.2)		H1N1	North America	swine
A/swine/Oklahoma/A02245006/2019	CS (1A.2-3-like)		H1N2	North America	swine
A/swine/Mexico/AVX62/2014	CS (1A.3.1)		H1N1	North America	swine
A/swine/Mexico/Mex52/2010	CS (1A.3.1)	EPI_ISL_128164	H1N1	North America	swine
A/swine/Oklahoma/SG1188/2004	CS (1A.3.1)	EPI_ISL_151616	H1N1	North America	swine
A/swine/Minnesota/A01201429/2011	CS (1A.3.1)	EPI_ISL_119366	H1N2	North America	swine
A/swine/Texas/00244/2004	CS (1A.3.1)	EPI_ISL_151254	H1N1	North America	swine
A/swine/Illinois/A01047930/2011	CS (1A.3.1)	EPI_ISL_116935	H1N1	North America	swine
A/swine/Missouri/A01932424/2017	CS (1A.3.1)	EPI_ISL_261458	H1N1	North America	swine
A/swine/Kentucky/SG1167/2003	CS (1A.3.1)	EPI_ISL_151609	H1N1	North America	swine
A/swine/Virginia/A01202625/2011	CS (1A.3.3.3)	EPI_ISL_127783	H1N1	North America	swine
A/swine/Illinois/A01240830/2011	CS (1A.3.3.3)	EPI_ISL_130052	H1N2	North America	swine
A/swine/Iowa/A02636482/2022	CS (1A.3.3.3)	EPI_ISL_14777888	H1N1	North America	swine
A/swine/North_Carolina/1593414/2015	CS (1A.3.3.3)	EPI_ISL_4082105	H1N1	North America	swine
A/swine/OH/511445/2007	CS (1A.3.3.3)	EPI_ISL_28826	H1N1	North America	swine
A/swine/Indiana/14TOSU3381/2014	CS (1A.3.3.3)	EPI_ISL_200854	H1N1	North America	swine
A/swine/South_Dakota/A02478533/2019	CS (1A.3.3.3)	EPI_ISL_372502	H1N1	North America	swine
A/swine/YokohamaAQ/1/2014	CS (1A.3.3.3)	EPI_ISL_233607	H1N1	Japan	swine
A/swine/California/T1800664/2018	CS (1A.3.3.3)	EPI_ISL_372884	H1N1	North America	swine
A/swine/Iowa/A01672824/2017	CS (1A.3.3.3)	EPI_ISL_258617	H1N1	North America	swine
A/swine/Michigan/A01259077/2017	CS (1A.3.3.3)	EPI_ISL_278198	H1N2	North America	swine
A/swine/Indiana/A01781271/2016	CS (1A.3.3.3)	EPI_ISL_237040	H1N1	North America	swine
A/Minnesota/33/2014	CS (1A.3.3.3)	EPI_ISL_252683	H1N1	North America	human
A/swine/Minnesota/A02710695/2021	CS (1A.3.3.3)	EPI_ISL_14769988	H1N1	North America	swine
A/swine/Missouri/A01945149/2015	CS (1A.3.3.3)	EPI_ISL_201851	H1N1	North America	swine
A/Iowa/39/2015	CS (1A.3.3.3)	EPI_ISL_253936	H1N1	North America	human
A/swine/Missouri/A01796703/2015	CS (1A.3.3.3)	EPI_ISL_208983	H1N1	North America	swine
A/swine/Iowa/SJCEIRR-SU1529/2023	CS (1A.3.3.3)	EPI_ISL_18755194	H1N1	North America	swine
A/swine/South_Dakota/G85022NW/2022	CS (1A.3.3.3)	EPI_ISL_16854315	H1N1	North America	swine
A/swine/Nebraska/A02245292/2019	CS (1A.3.3.3)	EPI_ISL_399437	H1N2	North America	swine
A/Minnesota/46/2015	CS (1A.3.3.3)	EPI_ISL_30411	H1N1	North America	human
A/swine/Iowa/A01667089/2017	CS (1A.3.3.3)	EPI_ISL_264228	H1N1	North America	swine
A/swine/Iowa/A02636317/2021	CS (1A.3.3.3)	EPI_ISL_14770782	H1N1	North America	swine
A/swine/Iowa/18TOSU2664/2018	CS (1A.3.3.3)	EPI_ISL_399672	H1N1	North America	swine
A/swine/Ohio/OH-29101/2021	CS (1A.3.3.3)	EPI_ISL_17471919	H1N1	North America	swine
A/swine/Iowa/A02478629/2019	CS (1A.3.3.3)	EPI_ISL_393545	H1N1	North America	swine
A/swine/Minnesota/5763/2003	CS (1A.3.3.3)	EPI_ISL_95805	H1N2	North America	swine
A/swine/Korea/S11/2005	CS (1A.3.3.3)	EPI_ISL_10356	H1N2	South Korea	swine
A/Swine/Korea/K1/2005	CS (1A.3.3.3)		H1N2	South Korea	swine
A/swine/Korea/JL01/2005	CS (1A.3.3.3)	EPI_ISL_24574	H1N2	South Korea	swine
A/swine/Korea/Hongsong2/2004	CS (1A.3.3.3)	EPI_ISL_24573	H1N2	South Korea	swine
A/swine/Korea/Asan04/2006	CS (1A.3.3.3)	EPI_ISL_24577	H1N2	South Korea	swine
A/swine/Korea/JL04/2005	CS (1A.3.3.3)	EPI_ISL_24576	H1N2	South Korea	swine
A/swine/Korea/JL02/2005	CS (1A.3.3.3)	EPI_ISL_24575	H1N2	South Korea	swine
A/swine/Korea/VDS2/2009	CS (1A.3.3.3)	EPI_ISL_132825	H1N2	South Korea	swine
A/swine/Korea/PZ4/2006	CS (1A.3.3.3)	EPI_ISL_24578	H1N2	South Korea	swine
A/swine/Korea/1204/2009	CS (1A.3.3.3)	EPI_ISL_129445	H1N2	South Korea	swine
A/swine/Korea/CY12-03/2011	CS (1A.3.3.3)	EPI_ISL_142690	H1N2	South Korea	swine
A/swine/South_Korea/CVM-0121/2022	CS (1A.3.3.3)	EPI_ISL_18708356	H1N2	South Korea	swine

A/swine/South_Korea/KS60/2016	CS (1A.3.3.3)	EPI_ISL_381145	H1N2	South Korea	swine
A/swine/SouthKorea/GBKW-3/2016	CS (1A.3.3.3)		H1N2	South Korea	swine
A/swine/South_Korea/CNU-1902/2019	CS (1A.3.3.3)	EPI_ISL_18699021	H1N2	South Korea	swine
A/swine/South_Korea/CNU-1904/2019	CS (1A.3.3.3)	EPI_ISL_18699043	H1N2	South Korea	swine
A/swine/South_Korea/CNU-1903/2019	CS (1A.3.3.3)	EPI_ISL_18699042	H1N2	South Korea	swine
A/swine/South_Korea/s804/2018	CS (1A.3.3.3)	EPI_ISL_17885885	H1N2	South Korea	swine
A/swine/South_Korea/s806/2018	CS (1A.3.3.3)	EPI_ISL_17885989	H1N2	South Korea	swine
A/swine/South_Korea/s805/2018	CS (1A.3.3.3)	EPI_ISL_17885974	H1N2	South Korea	swine
A/swine/South_Korea/CVM-0656/2021	CS (1A.3.3.3)	EPI_ISL_18708384	H1N2	South Korea	swine
A/swine/South_Korea/GN-1/2018	CS (1A.3.3.3)	EPI_ISL_16215749	H1N2	South Korea	swine
A/swine/South_Korea/GNJJ/2020	CS (1A.3.3.3)	EPI_ISL_16215764	H1N2	South Korea	swine
A/swine/TienGiang/01-22-2/2011	CS (1A.3.3.3)	EPI_ISL_164395	H1N2	South East Asia	swine
A/swine/Dong_Nai/07-28-3/2015	CS (1A.3.3.3)	EPI_ISL_218597	H1N2	South East Asia	swine
A/swine/Tien_Giang/08-10-1/2014	CS (1A.3.3.3)	EPI_ISL_218055	H1N2	South East Asia	swine
A/swine/DongNai/03-17-2/2011	CS (1A.3.3.3)	EPI_ISL_164462	H1N2	South East Asia	swine
A/swine/Ba_Ria_Vung_Tau/02-17-1/2015	CS (1A.3.3.3)	EPI_ISL_218275	H1N2	South East Asia	swine
A/swine/Tien_Giang/07-04-1/2015	CS (1A.3.3.3)	EPI_ISL_218306	H1N2	South East Asia	swine
A/swine/Hong_Kong/2237/2010	CS (1A.3.3.3)	EPI_ISL_96980	H1N2	China	swine
A/swine/Guangxi/13/2006	CS (1A.3.3.3)	EPI_ISL_11923	H1N2	China	swine
A/swine/Hebei/10/2006	CS (1A.3.3.3)	EPI_ISL_103827	H1N2	China	swine
A/swine/Guangdong/2670/2010	CS (1A.3.3.3)	EPI_ISL_96981	H1N2	China	swine
A/swine/Guangdong/1222/2006	CS (1A.3.3.3)	EPI_ISL_81517	H1N2	China	swine
A/California/07/2009	CS (1A.3.3.2) pdm09	EPI_ISL_93974	H1N1	North America	human
A/Sichuan/1/2009	CS (1A.3.3.2) pdm09	EPI_ISL_200780	H1N1	China	human
A/swine/Yamagata/11/2010	CS (1A.3.3.2) pdm09	EPI_ISL_134582	H1N1	Japan	swine
A/swine/South_Korea/KS61/2016	CS (1A.3.3.2) pdm09	EPI_ISL_381085	H1N1	South Korea	swine
A/swine/Korea/SCJ09/2009	CS (1A.3.3.2) pdm09	EPI_ISL_79825	H1N1	South Korea	swine
A/swine/Iowa/A01049578/2011	CS (1A.3.3.2) pdm09	EPI_ISL_97102	H1N1	North America	swine
A/swine/Mexico/11004418/2014	CS (1A.3.3.2) pdm09	EPI_ISL_295531	H1N2	North America	swine
A/swine/Saga/KU-FK1/2016	CS (1A.3.3.2) pdm09	EPI_ISL_277386	H1N1	Japan	swine
A/swine/Kagoshima/65/2012	CS (1A.3.3.2) pdm09	EPI_ISL_181290	H1N1	Japan	swine
A/swine/Gunma/1/2012	CS (1A.3.3.2) pdm09	EPI_ISL_142837	H1N2	Japan	swine
A/swine/Niigata/2/2019	CS (1A.3.3.2) pdm09	EPI_ISL_353280	H1N2	Japan	swine
A/swine/Niigata/1/2018	CS (1A.3.3.2) pdm09	EPI_ISL_334239	H1N2	Japan	swine
A/swine/Korea/SCJ11/2009	CS (1A.3.3.2) pdm09	EPI_ISL_79827	H1N1	South Korea	swine
A/swine/Mexico/10926242/2014	CS (1A.3.3.2) pdm09	EPI_ISL_295529	H1N2	North America	swine
A/swine/Hokkaido/3-170/2015	CS (1A.3.3.2) pdm09	EPI_ISL_221975	H1N1	Japan	swine
A/swine/Manitoba/D0338/2014	CS (1A.3.3.2) pdm09	EPI_ISL_207918	H1N2	North America	swine
A/swine/Italy/73449/2013	CS (1A.3.3.2) pdm09	EPI_ISL_145714	H1N1	Europe	swine
A/swine/Kagoshima/43-P84/2018	CS (1A.3.3.2) pdm09	EPI_ISL_369291	H1N1	Japan	swine
A/swine/Alberta/SD0242/2017	CS (1A.3.3.2) pdm09	EPI_ISL_378614	H1N1	North America	swine
A/swine/Saskatchewan/SD0053/2014	CS (1A.3.3.2) pdm09	EPI_ISL_207977	H1N1	North America	swine
A/swine/Ehime/01/2015	CS (1A.3.3.2) pdm09	EPI_ISL_277370	H1N1	Japan	swine
A/swine/Ehime/10/2015	CS (1A.3.3.2) pdm09	EPI_ISL_277371	H1N1	Japan	swine
A/swine/Nara/1/2013	CS (1A.3.3.2) pdm09	EPI_ISL_275575	H1N1	Japan	swine
A/swine/Mexico/8630529/2011	CS (1A.3.3.2) pdm09	EPI_ISL_195995	H1N1	North America	swine
A/swine/Aichi/KU-OE7/2017	CS (1A.3.3.2) pdm09	EPI_ISL_576565	H1N2	Japan	swine
A/swine/Minnesota/A01941304/2015	CS (1A.3.3.2) pdm09	EPI_ISL_208934	H1N1	North America	swine
A/swine/Chonburi/NIAH127329-067/2019	CS (1A.3.3.2) pdm09	EPI_ISL_515514	H1N1	South East Asia	swine
A/swine/Chonburi/NIAH110873-033/2012	CS (1A.3.3.2) pdm09	EPI_ISL_168405	H1N1	South East Asia	swine
A/swine/Chonburi/NIAH109558-063/2015	CS (1A.3.3.2) pdm09	EPI_ISL_236909	H1N1	South East Asia	swine
A/swine/Chonburi/NIAH107142-073/2018	CS (1A.3.3.2) pdm09	EPI_ISL_310060	H1N1	South East Asia	swine
A/swine/Chonburi/NIAH113219-057/2017	CS (1A.3.3.2) pdm09	EPI_ISL_277709	H1N1	South East Asia	swine
A/swine/Thailand/NS393/2009	CS (1A.3.3.2) pdm09	EPI_ISL_146839	H1N1	South East Asia	swine
A/swine/Tornitz/IDT14598/2012	CS (1A.3.3.2) pdm09	EPI_ISL_151089	H1N2	Europe	swine
A/swine/Holguin/121/2010	CS (1A.3.3.2) pdm09	EPI_ISL_97044	H1N1	Europe	swine
A/swine/Jangsu/434/2010	CS (1A.3.3.2) pdm09	EPI_ISL_104328	H1N1	China	swine
A/swine/Nanchang/F9/2010	CS (1A.3.3.2) pdm09	EPI_ISL_89638	H1N1	China	swine
A/swine/Saskatchewan/SD0163/2016	CS (1A.3.3.2) pdm09	EPI_ISL_282513	H1N2	North America	swine
A/swine/Kagoshima/37-6342/2018	CS (1A.3.3.2) pdm09	EPI_ISL_334031	H1N1	Japan	swine
A/swine/Miyazaki/SVB56-2/2016	CS (1A.3.3.2) pdm09	EPI_ISL_255364	H1N1	Japan	swine
A/swine/Thailand/CU-RP1/2010	CS (1A.3.3.2) pdm09	EPI_ISL_89961	H1N1	South East Asia	swine
A/swine/Saskatchewan/SD0052/2014	CS (1A.3.3.2) pdm09	EPI_ISL_207978	H1N1	North America	swine
A/swine/Serbia/3/2016	CS (1A.3.3.2) pdm09	EPI_ISL_501503	H1N1	Europe	swine
A/swine/Mexico/84703178820/2014	CS (1A.3.3.2) pdm09	EPI_ISL_295544	H1N1	North America	swine
A/swine/Korea/SCJ08/2009	CS (1A.3.3.2) pdm09	EPI_ISL_79824	H1N1	South Korea	swine
A/swine/Korea/SCJ01/2009	CS (1A.3.3.2) pdm09	EPI_ISL_79817	H1N1	South Korea	swine
A/swine/Aichi/18/2014	CS (1A.3.3.2) pdm09	EPI_ISL_275569	H1N2	Japan	swine
A/swine/Ontario/16-24/2012	CS (1A.3.3.2) pdm09	EPI_ISL_191719	H1N1	North America	swine
A/swine/Poland/1889614/2014	CS (1A.3.3.2) pdm09	EPI_ISL_206455	H1N1	Europe	swine

A/swine/Saskatchewan/SD0108/2015	CS (1A.3.3.2) pdm09	EPI_ISL_282706	H1N1	North America	swine
A/swine/TienGiang/03-10-1/2013	CS (1A.3.3.2) pdm09	EPI_ISL_162668	H1N2	South East Asia	swine
A/swine/Guatemala/CIP049-C3804/2018	CS (1A.3.3.2) pdm09	EPI_ISL_16209250	H1N1	North America	swine
A/swine/Gunma/43/2013	CS (1A.3.3.2) pdm09	EPI_ISL_275572	H1N2	Japan	swine
A/Guangdong/SWL3/2009	CS (1A.3.3.2) pdm09	EPI_ISL_62196	H1N1	China	human
A/swine/Saskatchewan/SD0205/2016	CS (1A.3.3.2) pdm09	EPI_ISL_282455	H1N1	North America	swine
A/swine/Kagoshima/23/2012	CS (1A.3.3.2) pdm09	EPI_ISL_181282	H1N1	Japan	swine
A/swine/Zhejiang/SW64/2014	CS (1A.3.3.2) pdm09	EPI_ISL_217172	H1N2	China	swine
A/swine/Virginia/A01432745/2013	CS (1A.3.3.2) pdm09	EPI_ISL_147152	H1N2	North America	swine
A/swine/Voltlage/21525/2015	CS (1A.3.3.2) pdm09	EPI_ISL_505341	H1N2	Europe	swine
A/swine/Leningrad_Region/RII-02/2017	CS (1A.3.3.2) pdm09	EPI_ISL_1095455	H1N2	Europe	swine
A/swine/Calhorn/18417/2013	CS (1A.3.3.2) pdm09	EPI_ISL_505283	H1N2	Europe	swine
A/swine/Iowa/A02636162/2021	CS (1A.3.3.2) pdm09	EPI_ISL_6816041	H1N1	North America	swine
A/swine/Kagoshima/SVB-167/2017	CS (1A.3.3.2) pdm09	EPI_ISL_290891	H1N1	Japan	swine
A/swine/Kagoshima/KU-FK1/2014	CS (1A.3.3.2) pdm09	EPI_ISL_277367	H1N1	Japan	swine
A/swine/Kagoshima/30/2015	CS (1A.3.3.2) pdm09	EPI_ISL_237923	H1N1	Japan	swine
A/swine/Wolfhagen/21852/2015	CS (1A.3.3.2) pdm09	EPI_ISL_505345	H1N2	Europe	swine
A/swine/Denmark/S04291-1/2020	CS (1A.3.3.2) pdm09	EPI_ISL_16209047	H1N2	Europe	swine
A/Denmark/36/2021	CS (1A.3.3.2) pdm09	EPI_ISL_8786194	H1N1	Europe	human
A/swine/Nordrhein-Westfalen/28/2024	CS (1A.3.3.2) pdm09	EPI_ISL_18889615	H1N1	Europe	swine
A/swine/Neuenkirchen/20946/2014	CS (1A.3.3.2) pdm09	EPI_ISL_505326	H1N2	Europe	swine
A/swine/Damme/21342/2015	CS (1A.3.3.2) pdm09	EPI_ISL_505338	H1N2	Europe	swine
A/swine/Germany/Bak47/2015	CS (1A.3.3.2) pdm09	EPI_ISL_239027	H1N2	Europe	swine
A/swine/Goldenbeck/22011/2015	CS (1A.3.3.2) pdm09	EPI_ISL_505348	H1N2	Europe	swine
A/swine/Spain/45690-9/2018	CS (1A.3.3.2) pdm09	EPI_ISL_9594282	H1N2	Europe	swine
A/swine/France/59-200250/2020	CS (1A.3.3.2) pdm09	EPI_ISL_9593332	H1N2	Europe	swine
A/swine/England/041406/2013	CS (1A.3.3.2) pdm09	EPI_ISL_195136	H1N1	Europe	swine
A/swine/England/072865/2013	CS (1A.3.3.2) pdm09	EPI_ISL_195171	H1N1	Europe	swine
A/swine/Gunma/40/2013	CS (1A.3.3.2) pdm09	EPI_ISL_275576	H1N1	Japan	swine
A/swine/Saskatchewan/SD0274/2018	CS (1A.3.3.2) pdm09	EPI_ISL_373107	H1N1	North America	swine
A/swine/Northern_Ireland/2042-34/2022	CS (1A.3.3.2) pdm09	EPI_ISL_18221373	H1N1	Europe	swine
A/swine/Nagasaki/SV156/2014	CS (1A.3.3.2) pdm09	EPI_ISL_275568	H1N1	Japan	swine
A/swine/Garrel/IDT14844/2012	CS (1A.3.3.2) pdm09	EPI_ISL_151091	H1N2	Europe	swine
A/swine/Mexico/84704755140/2014	CS (1A.3.3.2) pdm09	EPI_ISL_295551	H1N1	North America	swine
A/swine/Korea/CY11-01/2011	CS (1A.3.3.2) pdm09	EPI_ISL_142686	H1N1	South Korea	swine
A/swine/Northern_Ireland/2016-14/2021	CS (1A.3.3.2) pdm09	EPI_ISL_18221342	H1N1	Europe	swine
A/swine/Aichi/1/2018	CS (1A.3.3.2) pdm09	EPI_ISL_314822	H1N1	Japan	swine
A/swine/Aichi/101/2018	CS (1A.3.3.2) pdm09	EPI_ISL_576566	H1N1	Japan	swine
A/swine/South_Korea/CVM-0629/2021	CS (1A.3.3.2) pdm09	EPI_ISL_18708364	H1N1	South Korea	swine
A/swine/South_Korea/CVM-0138/2022	CS (1A.3.3.2) pdm09	EPI_ISL_18708357	H1N1	South Korea	swine
A/swine/South_Korea/CVM-0139/2022	CS (1A.3.3.2) pdm09	EPI_ISL_18708358	H1N1	South Korea	swine
A/swine/South_Korea/CVM-0760/2021	CS (1A.3.3.2) pdm09	EPI_ISL_18708389	H1N1	South Korea	swine
A/swine/South_Korea/CVM-0878/2021	CS (1A.3.3.2) pdm09	EPI_ISL_18708439	H1N1	South Korea	swine
A/swine/South_Korea/CVM-01192/2022	CS (1A.3.3.2) pdm09	EPI_ISL_18708342	H1N1	South Korea	swine
A/swine/South_Korea/CVM-01193/2022	CS (1A.3.3.2) pdm09	EPI_ISL_18708354	H1N1	South Korea	swine
A/swine/South_Korea/CVM-01191/2022	CS (1A.3.3.2) pdm09	EPI_ISL_18708341	H1N1	South Korea	swine
A/swine/France/40-160098/2016	CS (1A.3.3.2) pdm09	EPI_ISL_381047	H1N1	Europe	swine
A/swine/Hokkaido/10/2013	CS (1A.3.3.2) pdm09	EPI_ISL_273570	H1N1	Japan	swine
A/swine/Aichi/007/2012	CS (1A.3.3.2) pdm09	EPI_ISL_275573	H1N1	Japan	swine
A/Paris/2251/2010	CS (1A.3.3.2) pdm09	EPI_ISL_164113	H1N1	Europe	human
A/Osaka/UT-A01/2013	CS (1A.3.3.2) pdm09	EPI_ISL_393910	H1N1	Japan	human
A/swine/BaRiaVungTau/02-03-2/2011	CS (1A.3.3.2) pdm09	EPI_ISL_161840	H1N1	South East Asia	swine
A/swine/Saskatchewan/D0515/2017	CS (1A.3.3.2) pdm09	EPI_ISL_378639	H1N1	North America	swine
A/swine/Saskatchewan/SD0017/2013	CS (1A.3.3.2) pdm09	EPI_ISL_207173	H1N1	North America	swine
A/North_Carolina/09/2012	CS (1A.3.3.2) pdm09	EPI_ISL_117047	H1N1	North America	human
A/swine/Chachoengsao/NIAH112882-046/2013	CS (1A.3.3.2) pdm09	EPI_ISL_167966	H1N1	South East Asia	swine
A/swine/Italy/321118-6/2014	CS (1A.3.3.2) pdm09	EPI_ISL_278850	H1N1	Europe	swine
A/swine/Germany/AR1760/2014	CS (1A.3.3.2) pdm09	EPI_ISL_238059	H1N1	Europe	swine
A/swine/Denmark/S02444-1/2020	CS (1A.3.3.2) pdm09	EPI_ISL_16209046	H1N1	Europe	swine
A/Denmark/1/2021	CS (1A.3.3.2) pdm09	EPI_ISL_909652	H1N1	Europe	human
A/Swine/Netherlands/GDE0091/2023	CS (1A.3.3.2) pdm09	EPI_ISL_18802145	H1N2	Europe	swine
A/swine/Germany/2022AI03599/2022	CS (1A.3.3.2) pdm09	EPI_ISL_17646247	H1N2	Europe	swine
A/Swine/Netherlands/GDE0048/2023	CS (1A.3.3.2) pdm09	EPI_ISL_18802005	H1N2	Europe	swine
A/swine/Germany/2021AI04886/2021	CS (1A.3.3.2) pdm09	EPI_ISL_17646293	H1N2	Europe	swine
A/swine/Germany/2022AI03362/2022	CS (1A.3.3.2) pdm09	EPI_ISL_17646249	H1N2	Europe	swine
A/Swine/Netherlands/GDE0101/2023	CS (1A.3.3.2) pdm09	EPI_ISL_18802192	H1N2	Europe	swine
A/swine/Guangxi/DX24/2013	CS (1A.3.3.2) pdm09	EPI_ISL_282076	H1N1	China	swine
A/Haiti/1790/2013	CS (1A.3.3.2) pdm09	EPI_ISL_145496	H1N1	North America	human
A/Vietnam/13V_H1-1/2013	CS (1A.3.3.2) pdm09	EPI_ISL_173295	H1N1	South East Asia	human

A/swine/Gunma/8-2368/2016	CS (1A.3.3.2) pdm09	EPI_ISL_232772	H1N1	Japan	swine
A/Vietnam/13V_H1-6/2013	CS (1A.3.3.2) pdm09	EPI_ISL_173298	H1N1	South East Asia	human
A/swine/Hanoi/788/2014	CS (1A.3.3.2) pdm09	EPI_ISL_17001880	H1N1	South East Asia	swine
A/swine/Italy/18-64849-32/2018	CS (1A.3.3.2) pdm09	EPI_ISL_4083704	H1N1	Europe	swine
A/swine/Mexico/10126158/2013	CS (1A.3.3.2) pdm09	EPI_ISL_195983	H1N1	North America	swine
A/swine/Hanoi/952/2013	CS (1A.3.3.2) pdm09	EPI_ISL_17001895	H1N1	South East Asia	swine
A/swine/Indiana/A02525081/2021	CS (1A.3.3.2) pdm09	EPI_ISL_4081983	H1N1	North America	swine
A/Okinawa/14T001/2014	CS (1A.3.3.2) pdm09	EPI_ISL_176332	H1N1	Japan	human
A/Shanghai-Xuhui/SWL1809/2019	CS (1A.3.3.2) pdm09	EPI_ISL_402382	H1N1	China	human
A/Iowa/53/2015	CS (1A.3.3.2) pdm09	EPI_ISL_206530	H1N1	North America	human
A/Mexico/2/2015	CS (1A.3.3.2) pdm09	EPI_ISL_222194	H1N1	North America	human
A/swine/Hanoi/894/2014	CS (1A.3.3.2) pdm09	EPI_ISL_17001892	H1N1	South East Asia	swine
A/swine/Tottori/41-6260/2018	CS (1A.3.3.2) pdm09	EPI_ISL_334038	H1N1	Japan	swine
A/swine/Nagasaki/1/2016	CS (1A.3.3.2) pdm09	EPI_ISL_255355	H1N1	Japan	swine
A/Puerto_Rico/49/2015	CS (1A.3.3.2) pdm09	EPI_ISL_211948	H1N1	North America	human
A/Saint-Petersburg/RII1073M/2016	CS (1A.3.3.2) pdm09	EPI_ISL_234003	H1N1	Europe	human
A/Volgograd/CRIE-4/2014	CS (1A.3.3.2) pdm09	EPI_ISL_166412	H1N1	Europe	human
A/swine/Aichi/30-36/2014	CS (1A.3.3.2) pdm09	EPI_ISL_274845	H1N1	Japan	swine
A/Phuket/325/2016	CS (1A.3.3.2) pdm09	EPI_ISL_246663	H1N1	South East Asia	human
A/swine/Gunma/40-P74/2018	CS (1A.3.3.2) pdm09	EPI_ISL_348516	H1N1	Japan	swine
A/Saitama-C/10/2016	CS (1A.3.3.2) pdm09	EPI_ISL_236833	H1N1	Japan	human
A/Michigan/45/2015	CS (1A.3.3.2) pdm09	EPI_ISL_202952	H1N1	North America	human
A/swine/Minnesota/A02135748/2017	CS (1A.3.3.2) pdm09	EPI_ISL_297073	H1N1	North America	swine
A/swine/Utah/A02636067/2021	CS (1A.3.3.2) pdm09	EPI_ISL_6781478	H1N1	North America	swine
A/Ekaterinburg/RII-4693S/2018	CS (1A.3.3.2) pdm09	EPI_ISL_322352	H1N1	Europe	human
A/Yunnan-Wuhua/1938/2016	CS (1A.3.3.2) pdm09	EPI_ISL_282778	H1N1	China	human
A/Ningxia-Xixia/SWL137/2020	CS (1A.3.3.2) pdm09	EPI_ISL_485488	H1N1	China	human
A/Sweden/15/2018	CS (1A.3.3.2) pdm09	EPI_ISL_307076	H1N1	Europe	human
A/Seoul/224/2016	CS (1A.3.3.2) pdm09	EPI_ISL_277243	H1N1	South Korea	human
A/swine/France/72-160174/2016	CS (1A.3.3.2) pdm09	EPI_ISL_381049	H1N1	Europe	swine
A/Alaska/01/2016	CS (1A.3.3.2) pdm09	EPI_ISL_214953	H1N1	North America	human
A/swine/Okayama/1/2019	CS (1A.3.3.2) pdm09	EPI_ISL_377135	H1N1	Japan	swine
A/Daejeon/1455/2018	CS (1A.3.3.2) pdm09	EPI_ISL_170673	H1N1	South Korea	human
A/Daegu/1454/2018	CS (1A.3.3.2) pdm09	EPI_ISL_83274	H1N1	South Korea	human
A/Vietnam/026/2019	CS (1A.3.3.2) pdm09	EPI_ISL_395284	H1N1	South East Asia	human
A/swine/Tochigi/44-1/2018	CS (1A.3.3.2) pdm09	EPI_ISL_334034	H1N1	Japan	swine
A/Cardiff/4341/2017	CS (1A.3.3.2) pdm09	EPI_ISL_302460	H1N1	Europe	human
A/Hawaii/39/2017	CS (1A.3.3.2) pdm09	EPI_ISL_277880	H1N1	North America	human
A/Vietnam/Lisn18135/2018	CS (1A.3.3.2) pdm09	EPI_ISL_349785	H1N1	South East Asia	human
A/Oregon/17/2017	CS (1A.3.3.2) pdm09	EPI_ISL_284095	H1N1	North America	human
A/New_York/PV01482/2018	CS (1A.3.3.2) pdm09	EPI_ISL_17053640	H1N1	North America	human
A/swine/England/245622/2022	CS (1A.3.3.2) pdm09	EPI_ISL_18586958	H1N1	Europe	swine
A/Zhejiang-Nanxun/SWL11023/2019	CS (1A.3.3.2) pdm09	EPI_ISL_410625	H1N1	China	human
A/Yunnan-Simao/SWL188/2020	CS (1A.3.3.2) pdm09	EPI_ISL_485447	H1N1	China	human
A/Guatemala/019/2019	CS (1A.3.3.2) pdm09	EPI_ISL_364560	H1N1	North America	human
A/swine/Texas/A02246976/2021	CS (1A.3.3.2) pdm09	EPI_ISL_6795332	H1N1	North America	swine
A/swine/Indiana/22TOSU2835/2022	CS (1A.3.3.2) pdm09	EPI_ISL_17409523	H1N2	North America	swine
A/Iowa/05/2021	CS (1A.3.3.2) pdm09	EPI_ISL_7334894	H1N1	North America	human
A/Michigan/45/2019	CS (1A.3.3.2) pdm09	EPI_ISL_358906	H1N1	North America	human
A/SAITAMA/158/2018	CS (1A.3.3.2) pdm09	EPI_ISL_344508	H1N1	Japan	human
A/swine/China/2327/2020	CS (1A.3.3.2) pdm09	EPI_ISL_18950679	H1N2	China	swine
A/Mississippi/30/2019	CS (1A.3.3.2) pdm09	EPI_ISL_398439	H1N1	North America	human
A/Omsk/RII-1323S/2020	CS (1A.3.3.2) pdm09	EPI_ISL_407118	H1N1	Europe	human
A/Guangdong/GLW/2018	CS (1A.3.3.2) pdm09	EPI_ISL_335963	H1N1	China	human
A/South_Dakota/18/2019	CS (1A.3.3.2) pdm09	EPI_ISL_349798	H1N1	North America	human
A/Minnesota/03/2018	CS (1A.3.3.2) pdm09	EPI_ISL_296055	H1N1	North America	human
A/New_York/PV04697/2019	CS (1A.3.3.2) pdm09	EPI_ISL_17054497	H1N1	North America	human
A/Yamaguchi/35/2019	CS (1A.3.3.2) pdm09	EPI_ISL_413299	H1N1	Japan	human
A/Kyiv/31/2020	CS (1A.3.3.2) pdm09	EPI_ISL_495514	H1N1	Europe	human
A/Saskatchewan/RV4005/2018	CS (1A.3.3.2) pdm09	EPI_ISL_336824	H1N1	North America	human
A/Barbados/7019/2018	CS (1A.3.3.2) pdm09	EPI_ISL_353430	H1N1	North America	human
A/North_Carolina/01/2021	CS (1A.3.3.2) pdm09	EPI_ISL_12109633	H1N1	North America	human
A/Baltimore/R0686/2019	CS (1A.3.3.2) pdm09	EPI_ISL_17614713	H1N1	North America	human
A/Zhejiang-Kecheng/SWL1976/2019	CS (1A.3.3.2) pdm09	EPI_ISL_402270	H1N1	China	human
A/NAGASAKI/1/2022	CS (1A.3.3.2) pdm09	EPI_ISL_13026389	H1N1	Japan	human
A/swine/Tottori/B34/2020	CS (1A.3.3.2) pdm09	EPI_ISL_14866753	H1N1	Japan	swine
A/Hawaii/70/2019	CS (1A.3.3.2) pdm09	EPI_ISL_397028	H1N1	North America	human
A/swine/South_Korea/CVM-0171/2022	CS (1A.3.3.2) pdm09	EPI_ISL_18708359	H1N1	South Korea	swine
A/swine/SouthKorea/BRI5/2020	CS (1A.3.3.2) pdm09		H1N1	South Korea	swine
A/Switzerland/36111/2022	CS (1A.3.3.2) pdm09	EPI_ISL_14328693	H1N1	Europe	human

A/Luxembourg/LNS0771005/2022	CS (1A.3.3.2) pdm09	EPI_ISL_17503018	H1N1	Europe	human
A/New_York/PV42740/2022	CS (1A.3.3.2) pdm09	EPI_ISL_16044205	H1N1	North America	human
A/swine/Iowa/19TOSU2724/2019	CS (1A.3.3.2) pdm09	EPI_ISL_707075	H1N1	North America	swine
A/Krasnodar/32/2019	CS (1A.3.3.2) pdm09	EPI_ISL_338310	H1N1	Europe	human
A/swine/Pskov_region/RII-8-2S/2023	CS (1A.3.3.2) pdm09	EPI_ISL_18837260	H1N1	Europe	swine
A/Iowa/59/2018	CS (1A.3.3.2) pdm09	EPI_ISL_330464	H1N1	North America	human
A/Novosibirsk/RII-27028S/2020	CS (1A.3.3.2) pdm09	EPI_ISL_497908	H1N1	Europe	human
A/Wisconsin/588/2019	CS (1A.3.3.2) pdm09	EPI_ISL_404460	H1N1	North America	human
A/New_York/PV07984/2020	CS (1A.3.3.2) pdm09	EPI_ISL_17056824	H1N1	North America	human
A/Romania/4750/2022	CS (1A.3.3.2) pdm09	EPI_ISL_16757852	H1N1	Europe	human
A/British_Columbia/123/2022	CS (1A.3.3.2) pdm09	EPI_ISL_14466482	H1N1	North America	human
A/Vietnam/0265/2023	CS (1A.3.3.2) pdm09	EPI_ISL_18857339	H1N1	South East Asia	human
A/Minnesota/40/2022	CS (1A.3.3.2) pdm09	EPI_ISL_15928557	H1N1	North America	human
A/France/ARA-HCL023021641001/2023	CS (1A.3.3.2) pdm09	EPI_ISL_17693440	H1N1	Europe	human
A/Stockholm/10/2021	CS (1A.3.3.2) pdm09	EPI_ISL_5743024	H1N1	Europe	human
A/England/224680746/2022	CS (1A.3.3.2) pdm09	EPI_ISL_16100802	H1N1	Europe	human
A/New_York/PV83325/2022	CS (1A.3.3.2) pdm09	EPI_ISL_17065818	H1N1	North America	human
A/France/NOR-IPP02297/2024	CS (1A.3.3.2) pdm09	EPI_ISL_18944326	H1N1	Europe	human
A/Thailand/CU-PEH231010/2023	CS (1A.3.3.2) pdm09	EPI_ISL_18939718	H1N1	South East Asia	human
A/France/ARA-RELAB-HCL023169048901/2023	CS (1A.3.3.2) pdm09	EPI_ISL_18585239	H1N1	Europe	human
A/Bangladesh/4014/2021	CS (1A.3.3.2) pdm09	EPI_ISL_5978883	H1N1	South East Asia	human
A/France/ARA-HCL022173253301/2022	CS (1A.3.3.2) pdm09	EPI_ISL_16311720	H1N1	Europe	human
A/New_Jersey/33/2022	CS (1A.3.3.2) pdm09	EPI_ISL_15928399	H1N1	North America	human
A/Nakhon_Phanom/P3705/2023	CS (1A.3.3.2) pdm09	EPI_ISL_18320333	H1N1	Europe	human
A/Nakhon_Phanom/P3470/2023	CS (1A.3.3.2) pdm09	EPI_ISL_18292930	H1N1	South East Asia	human
A/Michigan/49/2022	CS (1A.3.3.2) pdm09	EPI_ISL_15724356	H1N1	North America	human
A/France/ARA-HCL023018581401/2023	CS (1A.3.3.2) pdm09	EPI_ISL_17638540	H1N1	Europe	human
A/Louisiana/EVTL-100024/2022	CS (1A.3.3.2) pdm09	EPI_ISL_17673788	H1N1	North America	human
A/Wisconsin/67/2022	CS (1A.3.3.2) pdm09	EPI_ISL_15928563	H1N1	North America	human
A/France/PAC-HCL023130201701/2023	CS (1A.3.3.2) pdm09	EPI_ISL_18585144	H1N1	Europe	human
A/France/BRE-RELAB-IPP27065/2023	CS (1A.3.3.2) pdm09	EPI_ISL_18718272	H1N1	Europe	human
A/France/ARA-HCL023205544801/2023	CS (1A.3.3.2) pdm09	EPI_ISL_18885591	H1N1	Europe	human
A/Arizona/ASU107399/2023	CS (1A.3.3.2) pdm09	EPI_ISL_18928541	H1N1	North America	human
A/Chiang_Rai/P396/2024	CS (1A.3.3.2) pdm09	EPI_ISL_18841636	H1N1	South East Asia	human
A/Andalucia/PMC-00046/2023	CS (1A.3.3.2) pdm09	EPI_ISL_17539974	H1N1	Europe	human
A/swine/Moscow_region/RII-47/2023	CS (1A.3.3.2) pdm09	EPI_ISL_18837254	H1N1	Europe	swine
A/St._Petersburg/RII-23/2022	CS (1A.3.3.2) pdm09	EPI_ISL_17102844	H1N1	North America	human
A/France/OCC-RELAB-HCL024015547201/2024	CS (1A.3.3.2) pdm09	EPI_ISL_18885811	H1N1	Europe	human
A/Beijing/CY00002/2023	CS (1A.3.3.2) pdm09	EPI_ISL_18056704	H1N1	China	human
A/British_Columbia/PHL-1194/2023	CS (1A.3.3.2) pdm09	EPI_ISL_18665842	H1N1	North America	human
A/Schleswig-Holstein/19/2024	CS (1A.3.3.2) pdm09	EPI_ISL_18926975	H1N1	Europe	human
A/Andalucia/PMC-00152/2024	CS (1A.3.3.2) pdm09	EPI_ISL_18815778	H1N1	Europe	human
