# Supplementary material for: Pathogenicity and zoonotic potential of a novel pdm09-lineage H1N2 swine influenza A virus spatiotemporally linked to transboundary pig trade
Source: PLoS Pathog. 2026 Nov 3;22(11):e1014779. doi: 10.1371/journal.ppat.1014779 (PMC13743032; doi:10.1371/journal.ppat.1014779)
Supplement: Supplementary file 10 [file ppat.1014779.s012.pdf]

Response to Reviewers

Part I – Summary

Reviewer #1: In the manuscript by Kim and colleagues, the authors identify a novel swine H1N2 influenza virus strain and link it to an introduction from imported European pigs, which reassorted with a local South Korean strain. The authors then go on to characterize this novel swine H1N2 strain in vitro and in vivo, as compared to a reference strain, H1N1pdm09. Guinea pig studies indicate that this novel swine H1N2 is unable to transmit via the airborne route, however, in pigs this novel swine H1N2 strain replicated better than the H1N1pdm09 control and transmitted efficiently to contact pigs. Overall, the manuscript provides interesting data that is well discussed.

Response: We thank the reviewer for the positive assessment of our manuscript and for the constructive suggestions provided below.

Reviewer #2: The manuscript by Kim and coworkers describes the identification and characterization of a novel H1N2 swine influenza A virus (swIAV), A/swine/Korea/1030-3/202, detected through active surveillance efforts in South Korea. Phylogenetic analyses showed that the virus possesses 7 gene segments closely related to a Danish zoonotic pandemic-origin H1N1 (pdm09) virus, while containing a neuraminidase gene segment derived from endemic Korean swIAVs. The authors further charactered the virus in in vitro and in vivo studies in mice, guinea pigs and swine. Overall, the study addresses an important topic because continued evolution of pdm09-derived viruses in pigs remains a significant zoonotic concern. The manuscript is well-written, and the experiments are technically sound. The combination of phylogenetics, animal infection and transmission studies, receptor binding, and serology provide a descent, but not complete characterization of the virus. However, many of the conclusions extend beyond what the presented data demonstrate, and this some of the claims should be revised.

Response: We thank the reviewer for the constructive evaluation and for emphasizing the need to avoid overinterpretation of our findings. We agree that our in vitro and in vivo studies do not provide a complete characterization of this novel genotype and acknowledge that some statements in the original manuscript extended beyond what could be directly supported by the presented data. Accordingly, we substantially revised the manuscript to temper conclusions regarding the route of introduction, human infectivity, virulence, and pandemic risk. In addition, we incorporated the endemic Korean H1N2 swIAV A/swine/Korea/1027-1/2023 as an additional comparator in the in vitro and swine experiments, expanded the serological characterization to include hemagglutination inhibition and microneutralization assays, and revised the Discussion to more clearly distinguish risk-associated phenotypes from evidence of enhanced zoonotic or pandemic potential.

Part II – Major Issues: Key Experiments Required for Acceptance

Reviewer #1: none

Reviewer #2:

1. The central conclusion is that the virus entered Korea through imported pigs from Denmark. The evidence consists of: (a) phylogenetic clustering, (b) Bayesian phylogeography, (c) pig import data and (d) tMRCA estimates. These data certainly support a European origin. However, they do not demonstrate the transmission route. Do the authors have more specific information about the history of live animal imports to the farm the virus was isolated from? What about alternative introduction routes, such as (a) undetected circulation in other Asian countries that could act as intermediate countries? Could the authors elaborate about such possibilities in the discussion?

Response: We appreciate the reviewer's constructive suggestion. We agree that our data are insufficient to establish the precise transmission route of this virus. Unfortunately, farm-level records documenting the origin or movement history of the gilt from which A/swine/Korea/1030-3/2023 was isolated were not available. The virus was recovered from a recently introduced gilt, providing a temporal association with animal movement; however, the origin and prior movement history of this animal could not be independently verified.

We also agree that alternative introduction routes, including undetected circulation through other countries, cannot be excluded. Recent surveillance studies of H1 swIAVs in China have predominantly identified G4 Eurasian avian-like H1N1 viruses and other reassortant H1 viruses, and, to our knowledge, viruses closely corresponding to the European-associated genotype identified in the present study have not been reported in these surveillance datasets. However, the absence of such viruses from available surveillance data does not exclude their undetected circulation in China or other Asian countries, particularly given differences in surveillance intensity and the incomplete availability of swIAV sequence data across the region.

We therefore revised the manuscript to clarify that the phylogenetic clustering, Bayesian phylogeographic reconstruction, tMRCA estimates, and live-pig import records are consistent with a European origin and introduction in the context of international pig movement, but do not constitute direct evidence of transmission from Denmark to the source farm. Accordingly, we revised the Abstract, Results, Figure 2 legend, and Discussion to replace definitive statements regarding Denmark-to-Korea transmission with more cautious wording, including "European-associated," "spatiotemporally associated with international pig movement," and "consistent with introduction in the context of international pig movement." We also explicitly state that the precise introduction route remains

unresolved and that additional animal movement records and broader regional sequence data would be required to reconstruct the introduction pathway more confidently.

These revisions are reflected in the Abstract (Lines 29–31 and 44–48), Results (Lines 173–185), and Discussion (Lines 371–393) of the revised manuscript. All line numbers cited in our responses refer to the clean version of the revised manuscript.

2. The authors clearly demonstrate that this is a novel reassortant virus. Between 2023 and 2026 have viruses with this gene constellation become established in swine in South Korea? Was this a one-time event or has there been sustained transmission of such viruses and further reassortments?

Response: We agree that determining whether viruses with this gene constellation became established in South Korean swine is important for interpreting the epidemiological significance of 1030-3. The present study is based on nationwide active swIAV surveillance conducted in 2023, during which 1030-3 was identified as a divergent H1N2 isolate from a newly introduced gilt. Comparable nationwide follow-up surveillance data for 2024–2026 were not available for the present analysis.

We revised the Results and Discussion accordingly and now describe 1030-3 as a novel reassortant detected during the 2023 surveillance period. We also explicitly acknowledge that its persistence and longer-term evolutionary trajectory remain unknown and that continued nationwide genomic surveillance will be required to determine whether related European-associated pdm09-derived reassortants persist, disappear, or generate additional reassortants with endemic Korean swIAV lineages.

The uncertainty regarding persistence and establishment after 2023, together with the need for continued surveillance, is now explicitly stated in the Discussion (Lines 384–393 and 500–504).

3. Comparison only against NL09 is somewhat problematic. The reviewer understands that conducting new in vitro and in vivo studies are expensive, but the comparison should have included swIAVs endemic in South Korea, not just a pandemic prototype virus, such as NL09. I would like the authors to elaborate about this in the discussion, and potentially tone down some of their conclusions.

Response: We agree with the reviewer that comparison with NL09 alone did not sufficiently contextualize the phenotype of 1030-3 relative to swIAVs endemic in South Korea. NL09 was initially included as an early human A(H1N1)pdm09 reference strain to provide a baseline for comparison with the pdm09-derived genetic background of 1030-3. However, we agree that an endemic swIAV comparator is necessary to distinguish phenotypes associated with general swine adaptation from those more specific to 1030-3.

In response, we included A/swine/Korea/1027-1/2023 (1027-1), an endemic Korean H1N2 swIAV belonging to the classical swine 1A.3.3.3 lineage, as an additional comparator. Its phylogenetic position is now indicated in revised Figure 1, and comparative data for 1027-1 have been incorporated into the in vitro characterization and pig infection and transmission experiments shown in revised Figures 3, 5, and 6.

Inclusion of 1027-1 showed that several phenotypes initially interpreted relative to NL09 are shared with an endemic, swine-adapted H1N2 virus. Both 1027-1 and 1030-3 replicated efficiently in polarized Calu-3 cells and preferentially bound α2,6-linked sialic acids. In pigs, both viruses produced substantial nasal shedding, reduced average daily weight gain, and efficient direct-contact transmission. However, the comparative outcomes varied depending on the measured parameter. Contact pigs exposed to 1030-3 showed a significantly greater cumulative shedding burden than those exposed to NL09 and a numerically greater burden than those exposed to 1027-1, whereas 1027-1 produced numerically greater macroscopic lung lesion scores than 1030-3.

Accordingly, we revised the Results, Abstract, Author Summary, and Discussion to avoid describing 1030-3 as uniformly more replicative, transmissible, or pathogenic than endemic Korean swIAVs. We now describe 1030-3 as a newly detected reassortant that replicates and transmits efficiently in pigs, while specifying the individual outcomes in which it differed from NL09 or 1027-1. These revisions place the phenotype of 1030-3 in a more appropriate swine-adapted context and substantially temper the original interpretation.

These revisions are reflected in the Abstract and Author Summary (Lines 34–66), the revised in vitro and pig Results (Lines 187–199 and 242–316), and the Discussion (Lines 399–415, 438–461, and 487–497). The endemic comparator is additionally included in revised Figures 1, 3, 5, and 6.

4. In vitro characterization in Calu-3 is useful, but again, with the lack of additional control viruses the conclusions drawn might be somewhat overinterpreted, and should be approached more cautiously. The same applies to guinea pig transmission data.

Response: We agree with the reviewer that the original comparison with NL09 alone did not adequately contextualize the in vitro phenotype of 1030-3 relative to endemic swIAVs. In response, we included the endemic Korean H1N2 swIAV A/swine/Korea/1027-1/2023 as an additional comparator in the Calu-3 replication, receptor-binding, and HA fusion-pH assays presented in revised Figure 3.

The revised data show that both 1027-1 and 1030-3 replicated efficiently in polarized Calu-3 cells and preferentially bound α2,6-linked sialic acids. Their HA fusion thresholds were also similar (pH 5.6 for 1027-1 and pH 5.7 for 1030-3), compared with pH 5.3 for NL09. These findings demonstrate that

efficient replication in Calu-3 cells and α2,6-linked receptor preference are not unique to 1030-3, but are shared with an endemic Korean swIAV comparator. We therefore revised the Abstract, Results, and Discussion to avoid interpreting these phenotypes as evidence of uniquely enhanced human infectivity or adaptation. Instead, we describe them as features relevant to zoonotic-risk assessment when considered together with the genomic and antigenic characteristics of 1030-3.

We did not include 1027-1 in the guinea pig transmission experiment. We therefore agree that these data cannot establish whether the transmission phenotype of 1030-3 differs from that of endemic Korean swIAVs. Accordingly, we revised the Results and Discussion to report the guinea pig findings descriptively: 1030-3 transmitted to a subset of direct-contact animals but showed no detectable aerosol transmission under the conditions tested. We also explicitly acknowledge the absence of an endemic swIAV comparator as a limitation and removed broader claims regarding enhanced mammalian transmissibility.

The revised in vitro and guinea pig findings are presented in the Results (Lines 187–199 and 234–240), and the corresponding limitations and more cautious interpretation are discussed at Lines 399–415 and 429–437.

5. Swine experiments demonstrate increased fitness but not increased in virulence, which is somewhat expected. Again, here the lack of appropriate controls (endemic swIAVs) is problematic. Have the authors conducted similar studies with endemic swIAVs? If so adding this information would enforce the conclusions of this manuscript.

Response: We agree with the reviewer that efficient replication and transmission in pigs should not be interpreted as evidence of uniformly increased virulence, and that comparison with an endemic swIAV is important for contextualizing the phenotype of 1030-3.

In response, we included the endemic Korean H1N2 swIAV A/swine/Korea/1027-1/2023 as an additional comparator in the pig infection and direct-contact transmission experiment. The revised data show that both 1027-1 and 1030-3 replicated efficiently in pigs and transmitted to all direct-contact animals. Contact pigs exposed to 1030-3 showed the highest cumulative nasal shedding burden over 0–10 dpc, which was significantly greater than that observed in the NL09 group and numerically greater than that in the 1027-1 group. However, 1030-3 did not show uniformly greater pathogenicity than the endemic comparator; for example, 1027-1 produced numerically greater macroscopic lung lesion scores.

We therefore revised the Results and Discussion to distinguish efficient replication and transmission from virulence and removed statements implying that 1030-3 is uniformly more fit or pathogenic than

endemic Korean swIAVs. We now describe 1030-3 as a virus that replicates and transmits efficiently in pigs, while specifying the individual outcomes in which it differed from NL09 or 1027-1.

The comparative pig data are presented in the revised Results (Lines 242–316), and the distinction between efficient replication/transmission and virulence is incorporated into the Discussion (Lines 438–461).

6. The cytokine and flow cytometry analyses are probably the weakest experimental section. Several findings are merely trends. Without longitudinal sampling or functional assays, the biological significance remains speculative.

Response: We agree with the reviewer that the cytokine and flow cytometry analyses should be interpreted as descriptive and exploratory because they were performed at a single time point, involved relatively small experimental groups, and were not accompanied by functional validation. We therefore substantially revised this section to remove causal or mechanistic interpretations and to clearly distinguish statistically supported findings from numerical trends.

Specifically, the revised Results now report the significant increase in IL-12p40 concentrations in 1027-1-infected pigs and the significant increase in $CD4^+$ αβ T-cell frequency in 1030-3-infected pigs separately from non-significant numerical changes in alveolar macrophages, total αβ T cells, and $CD8^+$ αβ T cells. We now describe these observations as strain-associated differences in the local respiratory immune environment at 5 dpi rather than as mechanisms underlying the replication or transmission phenotype of 1030-3.

We also revised the Discussion to explicitly acknowledge that the single-time-point design, limited group size, and absence of functional assays preclude mechanistic interpretation. Additional longitudinal and functional studies will be required to determine whether these immune changes contribute to viral replication, pathology, or transmission.

The revised cytokine and flow-cytometry Results are presented at Lines 300–316, and the limitations of these exploratory analyses are explicitly discussed at Lines 449–461.

7. Only 13 human sera were tested, which makes this part of the analysis underpowered, and thus the conclusions debatable. I would have preferred to see hemagglutination inhibition (HAI) assays as well, since HAI is considered the golden standard for influenza serology and corelate of protection. I would also conduct serological testing using not only a broader panel of human sera, but also ferret and swine sera.

Response: We agree with the reviewer that the original human serum panel was limited in size and that hemagglutination inhibition (HI) analysis would provide an important complementary measure of HA-directed antibody recognition. In response, we performed HI assays using the available human serum panel (n = 13) against the recent human pdm09 H1N1 strain A/Hawaii/70/2019 (Hawaii2019), the endemic Korean H1N2 swIAV A/swine/Korea/1027-1/2023 (1027-1), and 1030-3. We also expanded the microneutralization analysis to include 1027-1 as an endemic swIAV comparator. These new data are presented in revised Figure 7.

The HI results showed substantial inter-individual variability. HI titers against 1027-1 were significantly lower than those against Hawaii2019, whereas the difference between Hawaii2019 and 1030-3 did not reach statistical significance. In contrast, microneutralization assays revealed significantly lower neutralizing titers against 1030-3 than against both Hawaii2019 and 1027-1. Thus, although the HI data did not demonstrate uniformly reduced HA-directed recognition of 1030-3, the MN data showed reduced functional cross-neutralization of 1030-3 relative to both the human seasonal H1N1 strain and the endemic Korean H1N2 comparator. Reference antiserum against a recent H1N1 vaccine antigen also showed lower neutralizing activity against 1030-3 than against the human pdm09 H1N1 comparator viruses.

We agree that inclusion of a broader human serum panel, as well as ferret and swine sera, would provide additional information on cross-reactive immunity and antigenic relationships. These additional serum panels were not available for the present revision. We therefore revised the Results and Discussion to ensure that our conclusions reflect the scope of the serum panel examined and now describe the serological findings as evidence of reduced cross-neutralizing antibody recognition within this panel. We also clarify that broader serological studies would be valuable for further defining the extent of cross-reactive immunity against 1030-3.

The new serological data are presented in revised Figure 7 and the Results (Lines 317–357). The HI, MN, and reference-antiserum neutralization procedures are described in the Materials and methods (Lines 649–685), and the scope and limitations of the human serum findings are discussed at Lines 469–486.

8. Pandemic risk discussion is exaggerated. Receptor binding to α 2.6 SA receptors and replication in Calu-3s are common features of most, if not all, swine influenza viruses. Again, the lack of appropriate control viruses leads to exaggerated conclusions. Additional studies, such as transmission in ferrets, replication in primary respiratory cells or human airway organoids, antigenic characterization in a broader panel of human sera, would be useful. For these reasons, the statement that the virus has "high zoonotic potential" is stronger than warranted.

Response: We agree with the reviewer that our original wording overstated the zoonotic and pandemic implications of the data. In particular, efficient replication in Calu-3 cells and preferential binding to α2,6-linked sialic acids are not unique indicators of enhanced human adaptation, as these properties were also observed for the endemic Korean H1N2 swIAV 1027-1 in the revised experiments. In addition, 1030-3 transmitted only to a subset of direct-contact guinea pigs and showed no detectable aerosol transmission under the conditions tested.

We therefore removed statements describing 1030-3 as having "high zoonotic potential" and revised the Abstract, Author Summary, Results, and Discussion to distinguish individual risk-associated features from evidence of enhanced zoonotic or pandemic potential. We now describe 1030-3 as a reassortant that combines efficient replication and transmission in pigs, α2,6-linked receptor preference, replication in a human bronchial epithelial cell model, and reduced cross-neutralizing antibody recognition. Importantly, the revised serological analyses showed that microneutralization titers against 1030-3 were significantly lower than those against both the recent human H1N1 strain Hawaii2019 and the endemic Korean H1N2 comparator 1027-1, while antiserum against a recent H1N1 vaccine antigen also showed reduced neutralization of 1030-3.

At the same time, we explicitly state that these combined findings do not establish efficient human transmission or high pandemic potential. We also acknowledge that a more comprehensive risk assessment would require additional studies, including ferret transmission experiments, replication in primary human respiratory cells or airway organoids, and serological testing using broader human and animal serum panels.

Accordingly, the revised Discussion now frames the significance of 1030-3 in terms of the convergence of a novel European-associated pdm09-derived genomic background, efficient replication and transmission in pigs, and reduced cross-neutralizing antibody recognition, rather than any single phenotype being interpreted as evidence of high zoonotic or pandemic potential.

These changes are reflected throughout the revised Abstract and Author Summary (Lines 34–66) and are explicitly incorporated into the Discussion (Lines 394–415, 429–437, and 469–505), including our statement that the available data do not establish efficient human transmission or high pandemic potential (Lines 496–497).

Part III – Minor Issues: Editorial and Data Presentation Modifications

Reviewer #1:

1) The result sections are written in different styles. The last 2 sections about the serology have summary statements at the end of each section, which helps the reader. This should be applied across all the results. Similarly, some of the materials and methods sections have sufficient detail but others are written very superficially, please make them more uniform in level of detail.
Some of the figures have a poor resolution and need to be improved.

Response: We thank the reviewer for these helpful suggestions. We revised the Results sections to provide a more consistent structure and added or refined brief concluding statements at the end of the major result sections. We also revised the Materials and methods to provide a more uniform level of experimental detail, including expanded or clarified descriptions of the phylogeographic analysis, Calu-3 air–liquid interface infection, receptor-binding and HA fusion-pH assays, pig infection and co-housing design, respiratory viral-load measurements, cytokine analysis, BAL-cell flow cytometry, and serological assays. In addition, figures requiring improvement were regenerated and replaced with higher-resolution versions to improve readability and presentation.

Examples of the revised concluding statements appear at Lines 151–152, 197–199, 238–240, 275–277, and 313–316. Additional methodological detail was incorporated throughout the Materials and methods, including the phylogeographic analysis (Lines 548–566), in vitro assays (Lines 581–598), pig experiment (Lines 631–648), and serological assays (Lines 649–685).

2) Figure 2E would be more appropriate as a graphical abstract instead of a main figure.
For figure 7A do the authors have access to the birth years for each of the individuals? That would be nice to include in the supplemental table.

Response: We agree that the schematic previously shown as Figure 2E functions better as a graphical summary than as a primary data panel. We therefore removed this panel from the main Figure 2 and adapted it as the Graphical Abstract. Revised Figure 2 now focuses on the time-scaled phylogeny, genotype constellation, live-pig importation data, and phylogeographic reconstruction.

We also agree that donor age information provides useful context for interpretation of the human serological data. Birth year information was available for all 13 individuals, and we have added these data to the revised Supplementary Table S2, together with information on seasonal influenza vaccination and documented influenza infection history.

The revised Figure 2 is described at Lines 161–171, and donor metadata, including birth year, are now specified in the Materials and methods (Lines 649–657) and provided in revised Supplementary Table S2.

3) Can the authors clarify the pig transmission experiment? Were donor and contact pigs housed 1:1 or as a group? Clearly, pigs are shedding greater amounts of 1030-3 but the statement that the virus transmitted faster seems a bit of a stretch since the authors are comparing 100% transmission to 75% transmission on day 1. Please explain or revise.

Response: We thank the reviewer for highlighting the need to clarify the pig transmission design. We revised the Materials and methods, Results, and Figure 5 legend to specify that four inoculated donor pigs and four naïve contact pigs were co-housed as groups within each virus treatment, rather than maintained as 1:1 donor–contact pairs.

We also agree that our original statement that 1030-3 "transmitted faster" overstated the difference among groups. At 1 dpc, infectious virus was detected in 3 of 4 NL09 contact pigs, 2 of 4 1027-1 contact pigs, and all 4 1030-3 contact pigs. By 2 dpc, all contact pigs in all three virus groups were positive. We therefore removed the phrase "transmitted faster" and now report the observed detection frequencies and shedding kinetics directly.

In the revised Results, we state that all three viruses transmitted efficiently by direct contact, while 1030-3 produced the highest cumulative nasal shedding burden in contact pigs over 0–10 dpc. This burden was significantly greater than that observed in the NL09 group and numerically greater than that in the 1027-1 group. These revisions avoid overinterpreting the early detection difference while preserving the supported finding of a greater cumulative shedding burden for 1030-3 relative to NL09.

The group co-housing design is now explicitly described in the Materials and methods (Lines 631–637) and Figure 5 legend (Lines 252–266). The revised transmission frequencies and cumulative-shedding interpretation are presented in the Results (Lines 268–277).

Reviewer #2:
