# Supplementary material for: Pathogenicity and zoonotic potential of a novel pdm09-lineage H1N2 swine influenza A virus spatiotemporally linked to transboundary pig trade
Source: PLoS Pathog. 2026 Nov 3;22(11):e1014779. doi: 10.1371/journal.ppat.1014779 (PMC13743032; doi:10.1371/journal.ppat.1014779)
Supplement: Peer review history

# Peer review: Decision Letter 0

**DOI:** 10.1371/journal.ppat.1014779.r001

**Authors:** Thomas Hoenen

29 Jul 2026

Pathogenicity and zoonotic potential of a novel pdm09-lineage H1N2 swine influenza A virus spatiotemporally linked to transboundary pig trade

PLOS Pathogens

Dear Dr. Kim,

Thank you for submitting your manuscript to PLOS Pathogens. After careful consideration, we feel that it has merit but does not fully meet PLOS Pathogens's publication criteria as it currently stands. Therefore, we invite you to submit a revised version of the manuscript that addresses the points raised during the review process.

Please submit your revised manuscript by Sep 27 2026 11:59PM. If you will need more time than this to complete your revisions, please reply to this message or contact the journal office at plospathogens@plos.org. When you're ready to submit your revision, log on to https://www.editorialmanager.com/ppathogens/ and select the 'Submissions Needing Revision' folder to locate your manuscript file.

Please include the following items when submitting your revised manuscript:

* A letter that responds to each point raised by the editor and reviewer(s). You should upload this letter as a separate file labeled 'Response to Reviewers'. This file does not need to include responses to any formatting updates and technical items listed in the 'Journal Requirements' section below.

* A marked-up copy of your manuscript that highlights changes made to the original version. You should upload this as a separate file labeled 'Revised Manuscript with Track Changes'.

* An unmarked version of your revised paper without tracked changes. You should upload this as a separate file labeled 'Manuscript'.

We look forward to receiving your revised manuscript.

Kind regards,

Thomas Hoenen

Section Editor

PLOS Pathogens

Thomas Hoenen

Section Editor

Editor-in-Chief

PLOS Pathogens

orcid.org/0000-0003-2946-9497

Editor-in-Chief

PLOS Pathogens

orcid.org/0000-0002-7699-2064

**Additional Editor Comments (if provided):**

Reviews are positive, but both reviewers raise some points that should be adressed by text changes. Additionally, reviewer #2 is recommending experimental work, and I have issued a major revision decision to give you the time for such additional work. Should you consider this not necessary, please provide a detailed justification for this in your response to reviewers, and explain which changes to the text of the manuscript you have done to address their concerns.

**Journal Requirements:**

If the reviewer comments include a recommendation to cite specific previously published works, please review and evaluate these publications to determine whether they are relevant and should be cited. There is no requirement to cite these works unless the editor has indicated otherwise.

1) We do not publish any copyright or trademark symbols that usually accompany proprietary names, eg ©,  ®, or TM  (e.g. next to drug or reagent names). Therefore please remove all instances of trademark/copyright symbols throughout the text, including:

- ® on page: 23

- TM on page: 21.

2) Please upload all main figures as separate Figure files in .tif or .eps format. For more information about how to convert and format your figure files please see our guidelines:

https://journals.plos.org/plospathogens/s/figures

3) Please amend your detailed Financial Disclosure statement. This is published with the article. It must therefore be completed in full sentences and contain the exact wording you wish to be published.

i) State the initials, alongside each funding source, of each author to receive each grant. For example: "This work was supported by the National Institutes of Health (####### to AM; ###### to CJ) and the National Science Foundation (###### to AM)."

ii) State what role the funders took in the study. If the funders had no role in your study, please state: "The funders had no role in study design, data collection and analysis, decision to publish, or preparation of the manuscript."

iii) If any authors received a salary from any of your funders, please state which authors and which funders..

If you did not receive any funding for this study, please simply state: u201cThe authors received no specific funding for this work.u201d

4) Please send a completed 'Competing Interests' statement, including any COIs declared by your co-authors. If you have no competing interests to declare, please state "The authors have declared that no competing interests exist". Otherwise please declare all competing interests beginning with the statement "I have read the journal's policy and the authors of this manuscript have the following competing interests:"

**Reviewers' Comments:**

Reviewer's Responses to Questions

**Part I - Summary**

Please use this section to discuss strengths/weaknesses of study, novelty/significance, general execution and scholarship.

Reviewer #1: In the manuscript by Kim and colleagues, the authors identify a novel swine H1N2 influenza virus strain and link it to an introduction from imported European pigs, which reassorted with a local South Korean strain. The authors then go on to characterize this novel swine H1N2 strain in vitro and in vivo, as compared to a reference strain, H1N1pdm09. Guinea pig studies indicate that this novel swine H1N2 is unable to transmit via the airborne route, however, in pigs this novel swine H1N2 strain replicated better than the H1N1pdm09 control and transmitted efficiently to contact pigs. Overall, the manuscript provides interesting data that is well discussed.

Reviewer #2: The manuscript by Kim and coworkers describes the identification and characterization of a novel H1N2 swine influenza A virus (swIAV), A/swine/Korea/1030-3/202, detected through active surveillance efforts in South Korea. Phylogenetic analyses showed that the virus possesses 7 gene segments closely related to a Danish zoonotic pandemic-origin H1N1 (pdm09) virus, while containing a neuraminidase gene segment derived from endemic Korean swIAVs. The authors further charactered the virus in in vitro and in vivo studies in mice, guinea pigs and swine.

Overall, the study addresses an important topic because continued evolution of pdm09-derived viruses in pigs remains a significant zoonotic concern.

The manuscript is well-written, and the experiments are technically sound. The combination of phylogenetics, animal infection and transmission studies, receptor binding, and serology provide a descent, but not complete characterization of the virus.

However, many of the conclusions extend beyond what the presented data demonstrate, and this some of the claims should be revised.

**********

**Part II – Major Issues: Key Experiments Required for Acceptance**

Please use this section to detail the key new experiments or modifications of existing experiments that should be absolutely required to validate study conclusions.

Generally, there should be no more than 3 such required experiments or major modifications for a "Major Revision" recommendation. If more than 3 experiments are necessary to validate the study conclusions, then you are encouraged to recommend "Reject".

Reviewer #1: none

Reviewer #2: 1. The central conclusion is that the virus entered Korea through imported pigs from Denmark.

The evidence consists of: (a) phylogenetic clustering, (b) Bayesian phylogeography, (c) pig import data and (d) tMRCA estimates. These data certainly support a European origin. However, they do not demonstrate the transmission route. Do the authors have more specific information about the history of live animal imports to the farm the virus was isolated from? What about alternative introduction routes, such as (a) undetected circulation in other Asian countries that could act as intermediate countries? Could the authors elaborate about such possibilities in the discussion?

2. The authors clearly demonstrate that this is a novel reassortant virus. Between 2023 and 2026 have viruses with this gene constellation become established in swine in South Korea? Was this a one-time event or has there been sustained transmission of such viruses and further reassortments?

3. Comparison only against NL09 is somewhat problematic. The reviewer understands that conducting new in vitro and in vivo studies are expensive, but the comparison should have included swIAVs endemic in South Korea, not just a pandemic prototype virus, such as NL09. I would like the authors to elaborate about this in the discussion, and potentially tone down some of their conclusions.

4. In vitro characterization in Calu-3 is useful, but again, with the lack of additional control viruses the conclusions drawn might be somewhat overinterpreted, and should be approached more cautiously. The same applies to guinea pig transmission data.

5. Swine experiments demonstrate increased fitness but not increased in virulence, which is somewhat expected. Again, here the lack of appropriate controls (endemic swIAVs) is problematic. Have the authors conducted similar studies with endemic swIAVs? If so adding this information would enforce the conclusions of this manuscript.

6. The cytokine and flow cytometry analyses are probably the weakest experimental section. Several findings are merely trends. Without longitudinal sampling or functional assays, the biological significance remains speculative.

7. Only 13 human sera were tested, which makes this part of the analysis underpowered, and thus the conclusions debatable. I would have preferred to see hemagglutination inhibition (HAI) assays as well, since HAI is considered the golden standard for influenza serology and corelate of protection. I would also conduct serological testing using not only a broader panel of human sera, but also ferret and swine sera.

8. Pandemic risk discussion is exaggerated. Receptor binding to α 2.6 SA receptors and replication in Calu-3s are common features of most, if not all, swine influenza viruses. Again, the lack of appropriate control viruses leads to exaggerated conclusions. Additional studies, such as transmission in ferrets, replication in primary respiratory cells or human airway organoids, antigenic characterization in a broader panel of human sera, would be useful. For these reasons, the statement that the virus has "high zoonotic potential" is stronger than warranted.

**********

**Part III – Minor Issues: Editorial and Data Presentation Modifications**

Please use this section for editorial suggestions as well as relatively minor modifications of existing data that would enhance clarity.

Reviewer #1: The result sections are written in different styles. The last 2 sections about the serology have summary statements at the end of each section, which helps the reader. This should be applied across all the results. Similarly, some of the materials and methods sections have sufficient detail but others are written very superficially, please make them more uniform in level of detail.

Some of the figures have a poor resolution and need to be improved.

Figure 2E would be more appropriate as a graphical abstract instead of a main figure.

For figure 7A do the authors have access to the birth years for each of the individuals? That would be nice to include in the supplemental table.

Can the authors clarify the pig transmission experiment? Were donor and contact pigs housed 1:1 or as a group? Clearly, pigs are shedding greater amounts of 1030-3 but the statement that the virus transmitted faster seems a bit of a stretch since the authors are comparing 100% transmission to 75% transmission on day 1. Please explain or revise.

Reviewer #2: (No Response)

**********

PLOS authors have the option to publish the peer review history of their article (what does this mean?). If published, this will include your full peer review and any attached files.

If you choose “no”, your identity will remain anonymous but your review may still be made public.

**Do you want your identity to be public for this peer review?**  For information about this choice, including consent withdrawal, please see our Privacy Policy.

Reviewer #1: No

Reviewer #2: No

[NOTE: If reviewer comments were submitted as an attachment file, they will be attached to this email and accessible via the submission site. Please log into your account, locate the manuscript record, and check for the action link "View Attachments". If this link does not appear, there are no attachment files.]

**Figure resubmission:**

**Reproducibility:**

?>

# Author comment: Author response to Decision Letter 1

**DOI:** 10.1371/journal.ppat.1014779.r002

26 Aug 2026

Attachment Submitted filename: Response_to_Reviewers.pdf

# Peer review: Decision Letter 1

**DOI:** 10.1371/journal.ppat.1014779.r003

**Authors:** Thomas Hoenen

23 Oct 2026

Dear Dr. Kim,

We are pleased to inform that your manuscript, "Pathogenicity and zoonotic potential of a novel pdm09-lineage H1N2 swine influenza A virus spatiotemporally linked to transboundary pig trade", has been editorially accepted for publication at PLOS Pathogens.

Before your manuscript can be formally accepted and sent to production, you will need to complete our formatting changes, which you will receive by email within a week. Please note that your manuscript will not be scheduled for publication until you have made the required changes.

IMPORTANT NOTES

(1) Copyediting and Proofreading: The corresponding author will receive a typeset proof for review, to ensure errors have not been introduced during production. Please review the PDF proof of your manuscript carefully, as this is the last chance to correct any errors. Please note that major changes, or those which affect the scientific understanding of the work, will likely cause delays to the publication date of your manuscript.

(2) Appropriate Figure Files: Please remove all name and figure # text from your figure files. Please also take this time to check that your figures are of high resolution, which will improve the readbility of your figures and help expedite your manuscript's publication. Please note that figures must have been originally created at 300dpi or higher. Do not manually increase the resolution of your files. For instructions on how to properly obtain high quality images, please review our Figure Guidelines, with examples at: http://journals.plos.org/plospathogens/s/figures.

We encourage you to use PACE (http://pace.apexcovantage.com/), a figure-checking tool which helps authors ensure all figures meet PLOS requirements so that figures can publish in the highest quality. PACE is a digital diagnostic and conversion tool for figure files. It will provide information about any failed check(s) and, if able, will automatically convert the figure into an acceptable file that passes quality checks.

To use PACE, you must first register as a user. Then, login and navigate to the UPLOAD tab, where you will find detailed instructions on how to use the tool.

(3) Striking Image: Please upload a striking still image to accompany your article if one is available (you can include a new image or an existing one from within your manuscript). Should your paper be accepted, this image will be considered for our monthly issue image and may also appear on our website to feature your article. Please upload this as a separate file, selecting "striking image" as the file type upon upload. Please also include a separate "Other" file with a caption, including credits and any potential copyright information. Please do not include the caption in the main article file. If your image is from someone other than yourself, please ensure that the artist has read and agreed to the terms and conditions of the Creative Commons Attribution License at http://journals.plos.org/plospathogens/s/content-license. Please note that PLOS cannot publish copyrighted images.

(4) Press Release or Related Media: If you or your institution will be preparing press materials for this manuscript, please inform our press team in advance at plospathogens@plos.org as soon as possible. We ask that you contact us within one week to plan ahead of our fast Production schedule. If you need to know your paper's publication date for related media purposes, you must coordinate with our press team, and your manuscript will remain under a strict press embargo until the publication date and time.

(5) Update your Profile Information: Now that your manuscript has been provisionally accepted, please log into Editorial Manager and update your profile, if needed. Go to http://www.editorialmanager.com/ppathogens, log in, and click on the "Update My Information" link at the top of the page. Please update your user information to ensure an efficient production and billing process.

(6) LaTeX users only: Our staff will ask you to upload a TEX file in addition to the PDF before the paper can be sent to typesetting, so please carefully review our Latex Guidelines http://journals.plos.org/plospathogens/s/latex in the meantime.

Thank you again for supporting Open Access publishing; we are looking forward to publishing your work in PLOS Pathogens.

Best regards,

Thomas Hoenen

Section Editor

PLOS Pathogens

Thomas Hoenen

Section Editor

PLOS Pathogens

Dominique Soldati-Favre

Editor-in-Chief

PLOS Pathogens

***********************************************************

Reviewer's Responses to Questions

**Part I - Summary**

Please use this section to discuss strengths/weaknesses of study, novelty/significance, general execution and scholarship.<br/><br/>

Reviewer #1: The authors have made significant improvements to their original manuscript. The inclusion of another swine strain (1027-1) for the in vitro experiments and some animal studies have allowed more accurate conclusions to be drawn.

--------------------

**Part II – Major Issues: Key Experiments Required for Acceptance**

Please use this section to detail the key new experiments or modifications of existing experiments that should be absolutely required to validate study conclusions.<br/><br/>Generally, there should be no more than 3 such required experiments or major modifications for a "Major Revision" recommendation. If more than 3 experiments are necessary to validate the study conclusions, then you are encouraged to recommend "Reject".<br/><br/>

Reviewer #1: none

--------------------

**Part III – Minor Issues: Editorial and Data Presentation Modifications**

Please use this section for editorial suggestions as well as relatively minor modifications of existing data that would enhance clarity.<br/><br/>

Reviewer #1: none

--------------------

PLOS authors have the option to publish the peer review history of their article (what does this mean?). If published, this will include your full peer review and any attached files.

If you choose “no”, your identity will remain anonymous but your review may still be made public.

**Do you want your identity to be public for this peer review?**  For information about this choice, including consent withdrawal, please see our Privacy Policy.

Reviewer #1: No

# Editor report: Acceptance letter

**DOI:** 10.1371/journal.ppat.1014779.r004

**Authors:** Thomas Hoenen

Dear Dr. Kim,

We are delighted to inform you that your manuscript, "Pathogenicity and zoonotic potential of a novel pdm09-lineage H1N2 swine influenza A virus spatiotemporally linked to transboundary pig trade," has been formally accepted for publication in PLOS Pathogens.

We have now passed your article onto the PLOS Production Department who will complete the rest of the pre-publication process. All authors will receive a confirmation email upon publication.

Thank you again for supporting open-access publishing; we are looking forward to publishing your work in PLOS Pathogens.

Best regards,

Dominique Soldati-Favre

Editor-in-Chief

PLOS Pathogens
